# Nanomaterial-assisted CRISPR gene-engineering – A hallmark for triple-negative breast cancer therapeutics advancement

**DOI:** 10.1016/j.mtbio.2022.100450

**Published:** 2022-10-04

**Authors:** Jabeen Farheen, Narayan S. Hosmane, Ruibo Zhao, Qingwei Zhao, M. Zubair Iqbal, Xiangdong Kong

**Affiliations:** a Institute of Smart Biomedical Materials, School of Materials Science and Engineering, Zhejiang Sci-Tech University, Hangzhou, 310018, PR China; b Zhejiang-Mauritius Joint Research Centre for Biomaterials and Tissue Engineering, Zhejiang Sci-Tech University, Hangzhou, 310018, PR China; c Department of Chemistry & Biochemistry, Northern Illinois University, DeKalb, IL, 60115, USA; d Department of Materials, Imperial College London, London, SW7 2AZ, UK; e Research Center for Clinical Pharmacy & Key Laboratory for Drug Evaluation and Clinical Research of Zhejiang Province, The First Affiliated Hospital, Zhejiang University School of Medicine, Hangzhou, 310003, PR China

**Keywords:** CRISPR/Cas9, Genetic mechanism, Gene therapy, Nanocarriers, Triple-negative breast cancer

## Abstract

Triple-negative breast cancer (TNBC) is the most violent class of tumor and accounts for 20–24% of total breast carcinoma, in which frequently rare mutation occurs in high frequency. The poor prognosis, recurrence, and metastasis in the brain, heart, liver and lungs decline the lifespan of patients by about 21 months, emphasizing the need for advanced treatment. Recently, the adaptive immunity mechanism of archaea and bacteria, called clustered regularly interspaced short palindromic repeats (CRISPR) combined with nanotechnology, has been utilized as a potent gene manipulating tool with an extensive clinical application in cancer genomics due to its easeful usage and cost-effectiveness. However, CRISPR/Cas are arguably the efficient technology that can be made efficient *via* organic material-assisted approaches. Despite the efficacy of the CRISPR/Cas@nano complex, problems regarding successful delivery, biodegradability, and toxicity remain to render its medical implications. Therefore, this review is different in focus from past reviews by (i) detailing all possible genetic mechanisms of TNBC occurrence; (ii) available treatments and gene therapies for TNBC; (iii) overview of the delivery system and utilization of CRISPR-nano complex in TNBC, and (iv) recent advances and related toxicity of CRISPR-nano complex towards clinical trials for TNBC.

## Graphical abstract

**Figure:**
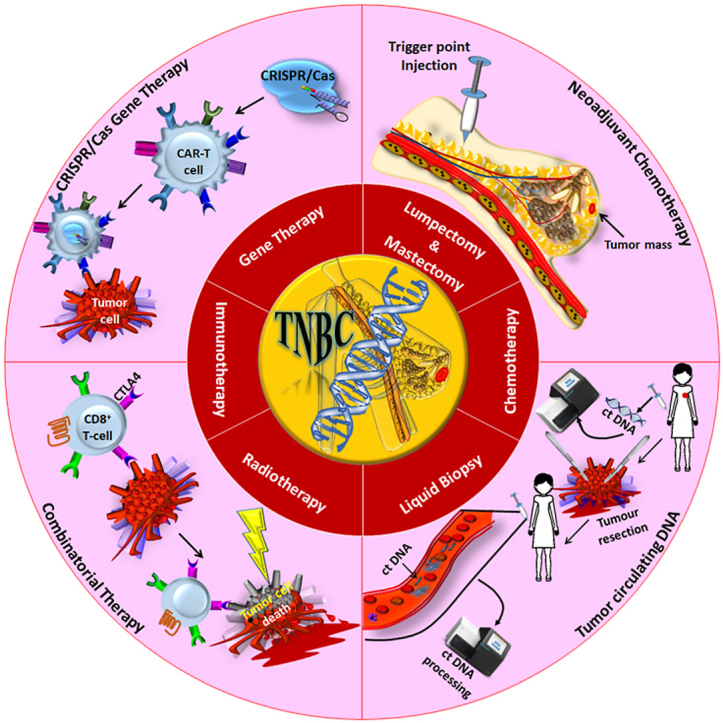

## 1 Introduction

Breast-related carcinoma (BC) is one of the potential sources of tumour-dependent death in females accounting for an expected 28% of new tumour cases [[1], [2], [3]]. It is an extremely diverse disease, and several signalling biomolecules and cascades facilitate its instigation and evolution [4,5]. According to genetic expression investigation, multiple subtypes of BC have been recognized based on the allelic expression profile and deficiency of the estrogen receptor (ER), progesterone receptor (PR), and human epidermal growth factor receptor 2 (HER2) [6]. Although ER-positive disease accounts for approximately 70–75%, triple-negative breast carcinoma (TNBC) appraises 24% of all newly BC conveyed cases [7,8], and 33% of patients embark relapse [9]^.^ However, 30% of BC deaths occur because of TNBC destructive pathology and metastasis [10,11]. It has the most rapid and aggressive metastasis, and progression compared to other forms of BC [3], and generally occurs in premenopausal women, who hardly survive longer than two years [12,13]. Additionally, TNBC has been conveyed to be more usual in either germline-based (20–25%), or somatic (5–10%) deficit in BRCA DNA repair associated (*BRCA*) gene [14], which shelters *TP53* mutation that deactivates p53 protein [3]. Nearly 48–66% of *BRCA1* deficit carriers nurture TNBC, a much higher incidence ratio than non-*BRCA1* deficit individuals [3,14]. Nevertheless, limited numbers of treatment alternatives are accessible due to the absence of expression receptors and lack of efficacious targeted medicine. In contrast, orthodox cytotoxic chemotherapies like Anthracyclines, Taxanes, Cytoxan, Carboplatin, and 5-Fluorouracil provide only limited survival benefits and induced ototoxicity, cardiotoxicity, neurotoxicity, and loss of fertility while, immunotherapy drugs such as Paclitaxel, Cobimetinib, Atezolizumab, and nab-Paclitaxel persuaded severe abdominal pain, vaginal bleeding, muscle cramps, paralysis, leukopenia, anemia, cardiotoxicity, and etc. [[9], [15], [16], [17], [18], [19], [20], [21]]. Besides, it is tremendously time-consuming and expensive to advance a new medicine for effective TNBC clinical treatment [22]. Thus, much research has been dedicated to the comprehension of the fundamental mechanisms leading to inducing tolerance or resistance. Currently, surplus therapies have been established to preclude acquired resistance, such as antibody-directed treatments [23], CAR-T cell-based advance immunotherapy [24] and targeted therapy or gene therapy like CRISPR/Cas system [1].

The bacterial and archaea's adaptive immunity system ​− ​clustered regularly interspaced short palindromic repeats (CRISPR)/allied Cas9 endonuclease, is the most promising and advanced gene-engineering technology due to its greater precision for targeting the mutated genome by the renowned Watson and Crick model of base pairing [25] that correct inaccuracy in the host genome [1], target the precise sequences [26], help to normalize tumour cell epigenome and high-throughput genome screening [27,28], contribute to detect proto-oncogenes [29,30], recognize drug resistance mechanism [31], establish tumour models in various cancers [32,33], and immunotherapy [34,35] as well as switch on [36] or off specific alleles [32,37,38] within the nucleus of the cell rapidly, economically, relatively easefully, and accurately than conventional gene-editing approaches [[39], [40], [41], [42]]. Mechanistically, the Cas-system relies on RNA-DNA recognition and adhesion for sequence-dependent DNA cleavage. It can be easefully programmed to induce double-strand breaks (DSBs) at any targeted site with the minimum cost. For example, CRISPR includes the short or single guide RNA (sgRNA) and endonuclease Cas9 protein to cleave DNA, which is the hybrid of *trans*-activating crRNA (tracrRNA) and CRISPR RNA (crRNA), respectively, to guide the cleavage at desired locations [43]. The active Cas9 and sgRNA complex identifies and nick the DNA sequence that is complementary to the ≈20 base pair sequences of sgRNA adjacent to the protospacer-adjacent motif (PAM) [21,33]. Currently, CRISPR/Cas9 expresses the great prospective to be pragmatic in gene therapy regimens for genetic disorders [44]. To accomplish the applied solicitations, the prerequisite is the effectual transfer of CRISPR/Cas9 into the cellular nucleus at specific target sites where the gene-engineering proceeds, in the form of the sgRNA with Cas9 mRNA, Cas9/sgRNA ribonucleoprotein (RNP), or plasmid coding Cas9 and sgRNA [28,40].

Importantly, direct penetration of CRISPR/Cas cargo by passing through several membranes, intracellular, and blood barriers along with functionalized sgRNA and Cas9 endonucleases are the critical challenge. Therefore, an effective delivery approach is the key to success in CRISPR/Cas-based gene manipulation [41]. However, safe and direct CRISPR/Cas cargo delivery at the targeted site [41], can be achieved *via* physical methods such as electroporation, microinjection, hydrodynamic, and physical stimulus method [41,45], and virus-mediated delivery [46] method. Both methods of the CRISPR payload delivery are highly effective in the wet-laboratory, nonetheless they can cause impairment due to the destruction of cell membranes and seditious reaction encouraged by immunogenicity, higher off-target impact, limited capacity of packaging, and high mass production cost [47,48]. Additionally, it may be shoddier that the ectopic chromosomal incorporation facilitated thru virus that can disturb the regulatory sequence of oncogenes or tumour suppressor genes leading to either secondary melanoma or more violent metastasis. In comparison, the nonviral nanovehicle arbitrated delivery of the CRISPR cargo is nontoxic, has ease of mass production and chemical alteration, cost-effective, large packaging capability, lesser immunogenicity issues, higher biocompatibility and protection against physiological environment-based degradation [40,49]. In this context, nanomaterials such as metal oxide, silicon, silica, plasmonic, lipids, and protein-based nanocarriers are becoming imperative clinical tools, yielding therapeutic precision and analytical functionality that would not be accomplished through large-scale methods [50]. It deals with various techniques for diagnosing, imaging, controlling, and delivering several types of payloads to the desired targeted site [39]. It supports the distribution of CRISPR/Cas at high efficiency and lesser toxicity that would overwhelm physiological obstacles [47]. The encapsulation via nano-based cargo precludes the susceptible CRISPR complex from deterioration intervened by proteases and nucleases in biological fluids matrix [51]. Since nucleic acid has a negative charge, besides the Cas9 nuclease's immense size (≈160 ​kDa), due to this nanocarriers are designed with positive charges to enhance the packaging capacity and effective communication with cells [[52], [53], [54]], or festooned with cell-penetrating peptides (CPPs) [30,55] to ease the plasma membrane penetration. However, its successful clinical application is a tremendous task due to low *in viv*o efficacy of nanocarriers [41].

This review first describes the molecular mechanism, possible genetic involvement, signalling pathways of TNBC occurrence, and the pivotal role of proteome and transcriptome in TNBC. Then, discuss the milestones of all ongoing treatments/therapies of TNBC, which were overcome via CRISPR/Cas gene engineering. We also discuss diverse CRISPR/Cas systems and nano-derived vehicles, including novel delivery, gene expression, and recent advances together with their implication in clinical trials. Nonetheless, the aim is to determine an inclusive summary of CRISPR/nano-based clinical trials, and breakthrough preclinical models of TNBC while considering future exertion. Although the review focuses on cancer, as many of the same principles apply to a host of other diseases, it may prove helpful to readers in a broad range of disciplines.

## 2 Triple-negative breast cancer

The triple-negative breast cancer (TNBC) is the most severe and highly violent type of breast carcinoma where none of the receptors (ER and PR) and HER2 protein are involved and frequently mutated in younger patients [56,57]. It shows aggressive progression, high mitotic index, metastasis rate/probability, histological level, and relapse [13]. It has heterogeneous immunohisto-molecular bases that are divided into many sub-classes such as mesenchymal, basal-like immune-suppressed (ER-/PR-/HER2-/IMlow), basal-like immune activated (ER-/PR-/HER2-/IM+), mesenchymal stem-like, luminal/androgen receptor (HER2-/PR-/AR+), metaplastic (0.2–5%), and immunomodulatory [13,58,59]. Regardless of this valuable classification, we have restricted knowledge about its pathogenesis due to the intense nature of heterogeneity [7], the mutation in various genes, transcription factors, proteome, and signalling pathways that make it utmost complex in nature for effective treatment.

### 2.1 Genetic mechanism of TNBC

**Fig. 1:**
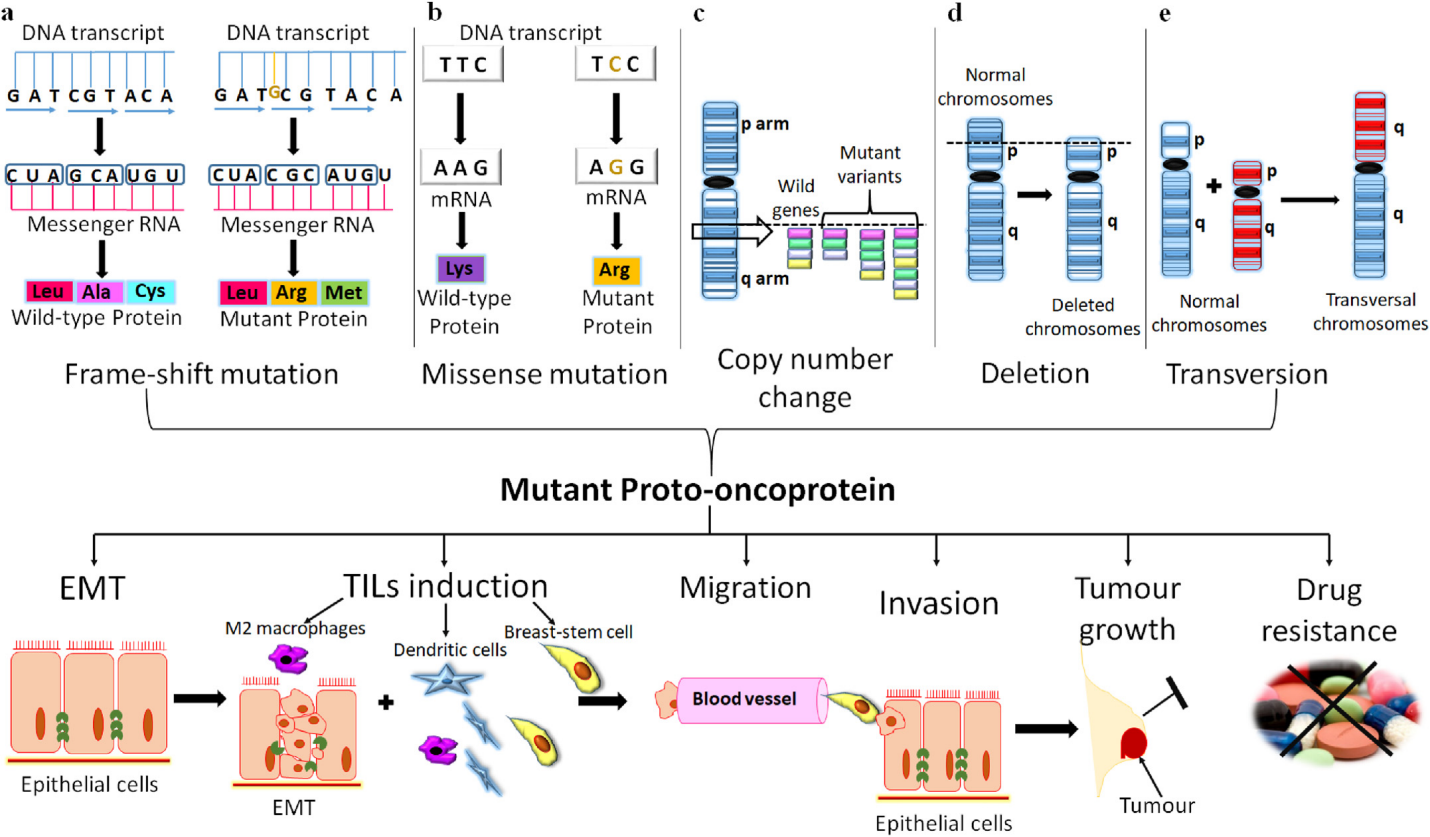
The genetic mechanism of triple-negative breast carcinoma in humans. (a) One nucleotide change has occurred in the DNA transcript frame resulting in alteration in amino acid as seen in wild-type DNA transcript GAT, CGT, and ACA code for leucine (Leu), alanine (Ala), and cysteine (Cys), respectively. Conversely, in mutant protein, insertion of “G” in the DNA transcript changed reading frame such as GAT, **G**CG, and TAC now code for leucine (Leu), **arginine** (Arg), and **methionine** (Met). (b) The faulty transcription alters a single nucleotide in the mRNA transcript causing amino acid changes like “**A**” (adenine) changes into “**G**” (guanine) aided arginine in place of lysine in the newly formed protein. (c) The changes in copy number usually increase the dose of single or multiple genes that frequently synthesize overly expressed mutant protein. (d) The *de novo* mutation either deletes the entire nucleotide sequences from the chromosome, or a single nucleotide with subsequent loss of the genetic information produces non-functional proteins or hyperactive protein. (e) In transversion, one arm (q) breaks and joins with either a homologous chromosome or a non-homologous chromosome arm (q). Transversion between homologous chromosomes duplicates the dose of genes that aberrant code proteins, which switch on other proteins and induce tumorigenesis.

The TNBC occurs due to the copy number change [60], polymorphism, missense mutation [3], transversions, frame-shift or non-sense mutation, and deletion [61] in the coding, enhancer, or promoter region of proto-oncogenes that code a mutated proto-oncoproteins which exhibited reduced tumour protective function [62,63]. However, basic knowledge of gene's product, namely “protein” structure and conformation, is essential to understand the complex genetic mechanism of TNBC. Proteins are made-up of amino acids chained by peptide bonds to each other in a linear fashion, while the carbon atom is located in the central part bound at four sites; two with amino acids, one with aminoacyl group, and the other with carboxyl group. All proteins have two significant domains; C-terminal or carboxyl-terminal (COOH) and N-terminal or amino-terminal (NH_2_). Usually, the C-terminal of the protein has a site for the DNA-binding, once mutation occurs in the C-terminal of protein loses its integrity, stability, recruitment of other factors, and chromatin binding [64,65] that can be studied via VIPUR (Fig. 1). The VIPUR scores successfully analyse any mutation in the conformation of protein structure and function. Its 0.8–10 score shows non-functionality/poor folding or mutation in protein, while lower VIPUR scores around 0.1–0.5 indicate more inclination toward wild-type protein structure [66,67]. Moreover, methylated CG repeats or CpG island presented on the proto-oncogene become frequently mutated and highly conserved over the evolutionary time scales [68]. Generally, in CG residues, cytosine (C) is deaminated into thymine (T) and guanine (G) into adenine (A). For instance, the *TP53* gene is about 74.4% responsible for TNBC in females globally [[69], [70], [71]]. Its gene product “p53” protein showed 90% missense mutation from CpG sites that span 190 diverse codons in the *TP53* gene exon that code the DNA-binding region of the p53 protein [68]. As a result, p53 losses its function, no or poor DNA binding, and restricted transcription [69,72,73] caused enhancement in tumour-infiltrating lymphocytes in the stroma [73], epithelial-mesenchymal transition, high rate of tumorigenesis, cellular invasion/migration, drug resistance, rapid cell division in TNBC cell lines [74,75], and large-sized tumour growth in females [76]. Similarly, the R330 frame-shift mutation in the coding region at the C-terminus of the *GATA3* gene leads to variations in epithelial to mesenchymal tissues causing overexpression and abnormal regulation of breast cell growth and progression. At the genetic level, the mutation in a single allele of the *GATA3* gene induces about 25% of its genomic redistribution and accumulation in TNBC [77].

### 2.2 Biomolecular signalling and pathways involved in TNBC

**Fig. 2:**
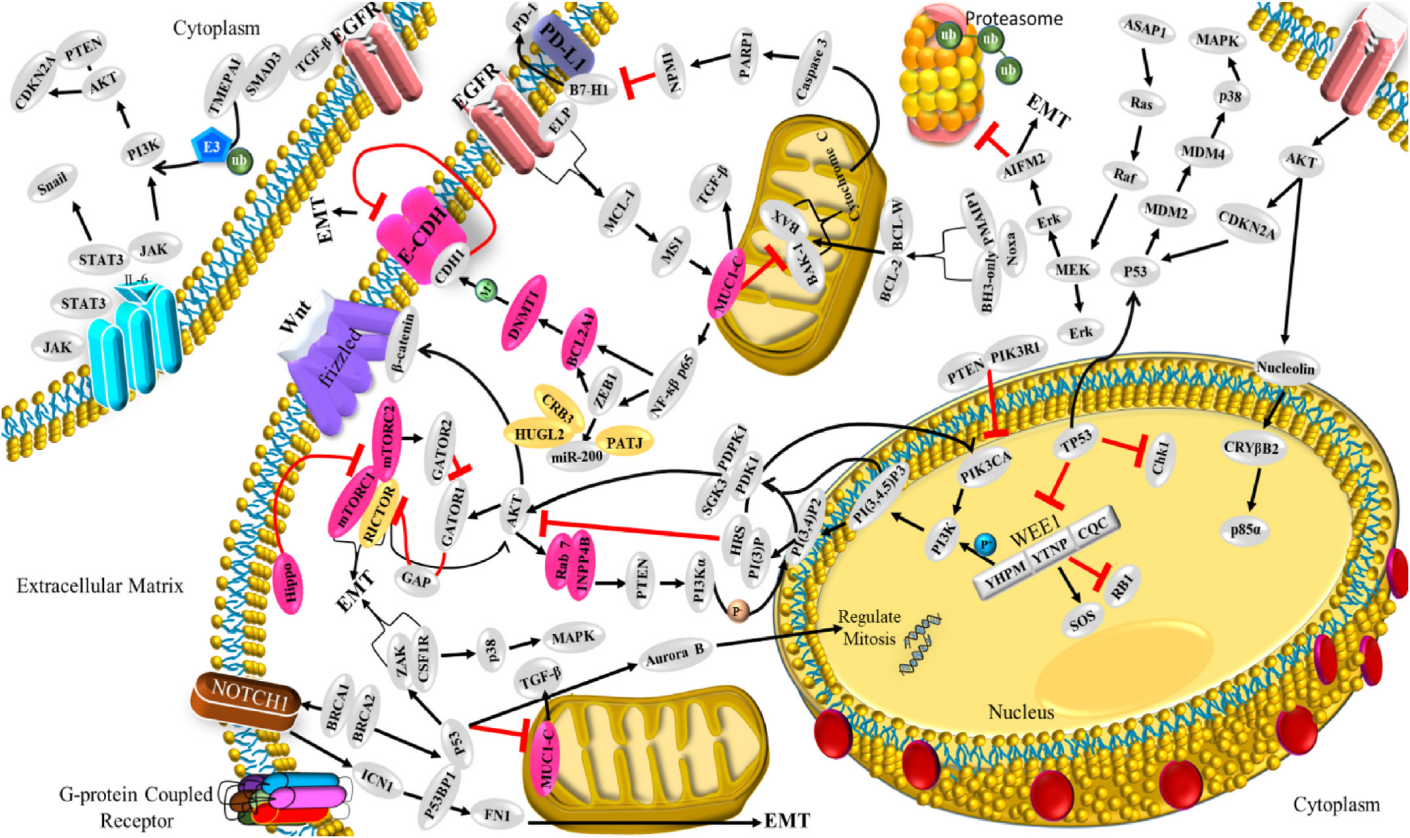
Summary of molecular signalling and cascades involve in triple-negative breast cancer tumor growth and progression. The red line shows the blockage or inhibition, the black line show induction, pink colour indicates up-regulation, yellow colour indicates down-regulation, M^+^ sign indicates methylation, P^+^ encapsulated in the blue circle indicates phosphorylation, P- encapsulated in the deep-orange circle shows dephosphorylation, ub stands for ubiquitin, E3; E3 ubiquitin ligase, EMT; epithelial to mesenchymal transmutation. (For interpretation of the references to colour in this figure legend, the reader is referred to the Web version of this article.)

Several genetic studies have been conducted on proto-oncogenes and their products to identify the genomic alteration during TNBC carcinogenesis [58]. Some recent studies revealed intense genetic involvement in TNBC, making it a more intimidating challenge to discover effective treatment [78]. Many more point mutation arises in multiple proto-oncogenes, such as 74% of somatic mutation occur in tumour suppressor protein 53 (TP53) gene, 5.6% in retinoblastoma 1 (RB1) gene, 5.6% in phosphatase and tensin homolog *(*PTEN) gene, and other in various proteome and singling pathways such as the mammalian target of rapamycin (*mTOR*), Hippo pathway, Sestri3/GATOR2/WDR59, B7-CD28, phosphatidylinositol3 kinase/protein kinase B (PI3K/AKT), nuclear factor kappa-light-chain-enhancer of activated B (NF-κβ), and Janus kinase/signal transducer and activator of transcription (JAK/STAT) singling cascade (Table 1, Fig. 2). Other than mentioned, the genome-wide approach identified 37 out of 130 genes dependencies during aggressive TNBC prognosis, and almost 13 other addiction genes become co-upregulated that are supposed to involve in DNA repair mechanism (*FANCL, PRKDC, CHEK1*, *DTL*, *RHNO1*, and *UBE2T*), mitotic cycle or transcriptomic factor inducing genes (*FOXM1, LIN9*, and *MYBL2*), spindle assembly checking (*BUB1, TTK, NUF2, RAD21,* and *BUB1B*), mitotic entry (*MASTL*), and bipolar division of centrosome-related (*KIFC1*) functions [74].

**Table 1:** Potential driver biomolecules regulated signalling cascades during triple-negative breast cancer prognosis and metastasis.

Driver genes	Trigger genes/proteomes	Signalling cascade	Related-function
Tumour cell proliferation			
***MCL-1***	*MUC1-C*	MCL-1→ MS1→ MUC1-C→ BCL2A1	Cell cycle regulation (S/G2, G2/M), EMT, and anti-cancer drug resistance [32]
***CDK***	EZH2	Cdc25A phosphatase→ CDK/cyclin E→ SOX9/EZH2 → FOXC1/Snail/Vimentin→ E-cadherin→ c-Myc/ATR kinase	Cell cycle regulation, oxidative stress-mediated Ras induction, promotes S-phase entry, replication fork stalling, nucleotide pool depletion, and G1-S phase DNA damage checkpoint [[79], [80], [81], [82]]
***CXCR4***	*CXCL12*	CXCR4→ CXCL12→ G-protein Gα-1→ downstream pathway	Violent conversion of the G1/S cycle [83]
***STAT3***	*JAK2*	IL-6→ JAK→ STAT3→Snail	Cell growth, cell cytoskeleton regulation, cell division, apoptosis, and suppression of other cell essential factors [[84], [85], [86]]
***NONO***	MSN	NONO→ MSN→ PKC→ CREB→ cAMP	Nuclear localization and phosphorylation of MSN [87,88]
***ADSL***	*C-Myc*	ADSL→ EglN2→ C-Myc	Suppress miR22HG, tumorigenesis [89]
***TEM8***	*ROCK1*	TEM8→ Rho C→ ROCK1→ SMAD5	Tumor-initiating cell propagation, vasculogenic mimicry, and neovasculogenesis [90]
***BRCA1***	*NOTCH1*	BRCA1→ NOTCH1→ ICN1→ FN1→ EMT	DNA damage repair, G2/M cell-cycle checkpoints, centrosome duplication, ubiquitination, and transcriptional regulation [91,92]
***BET* like BRD2/3/4 and BRDT**	*MYC* and *BCL2L1*	BET/MED1→ MYC/BCL2L1→ATR kinase	Transcriptional control, cell cycle, cellular differentiation, acetylation of histone tail, and transcriptional coactivator [93,94]
***TP53***	p53, MDM2 and MDM4	TP53→ p53/MDM2/MDM4→ p38 MAPK	Repair DNA, cell cycle control, and rapid early tumorigenesis control via *MIR30A* activation, ZEB2 inhibition [3,91,95]
Metastasis			
***CXCR7***	*CXCL12*	CXCR7→ CXCL12→ tumor cells→ VEGF	Tumor angiogenesis, adhesion of tumor cells with fibrin and endothelial cells, promote metastasis in lung, liver, lymphoma, and bone marrow [83]
***ICAM1***	LFA1/Mac1	ICAM1→ ICAM1-LFA1/ICAM1-Mac1→CDK6	Promote leukocyte adhesion to endothelium via intercellular interaction, leukocyte *trans*-endothelial migration, tumor cluster formation, circulating tumor cell development, and metastasis initiation [96]
***KTN1***	NF-ĸβ/p65	KTN1/Kinesin→ NF-ĸβ/Jak-Stats→ CXCL8 or IL-8	Focal adhesion development of cell lamella, organelle motility and cell shape, and migration *in vitro and in vivo* [97]
***AKT***	*FOXC1*	AKT/Wnt → H3K27ac/enhancer1/SSE245/HF-1α→ FOXC1→ E-cadherin→ MYC	Regulator protein synthesis, transcriptional reprograming of metastasis, spheroid, clonogenic growth, and relapse [4,98]
***ASAP1***	AIFM2, IL1B, MAP3K11 & TRAF1	RAS→ RAF →MEK →ERK	Upstream apoptosis regulation, G1/S phase transition, protein, and nucleotide metabolism, cell proliferation, promote invasion, and metastasis *in vitro* and *in vivo* [60]
Tumour microenvironment			
***FUT8***	*B7H3* or CD276	FUT8→B7H3/TGF-β → CD28	Regulate immune response, T-cell adaptive immunity, allogeneic T-cell activation, effector cytokine synthesis, cytotoxic T- lymphocyte reaction, and angiogenesis [13]
***CRYβB2***	Nucleolin	P85α→ CRYβB2→ Nucleolin→ AKT/EGFR→ CDKN2A→ p53, p21,p16	unfolded protein response, oxidative phosphorylation, DNA repair, decrease the expression of apoptosis genes, promotes de-differentiation, mesenchymal transmutation, cancer-associated fibroblasts, and enlargement of nucleoli [99]
***PIK3CA***	*PI3K*	PIK3CA→ PI3K→ AKT→ GATOR2→ mTORC1→ RICTOR	Intracellular signalling pathways such as PI3K-AKT-mTOR, involve in the synthesis of p110a protein, mammary tumor growth promotion, add oxygen and phosphorous cluster, and transport of cellular material [29,59]
***NSDHL***	TGF-β	NSDHL→ TGFβ→ TGFβR2/P-SMAD3→ LDLR/Cholesterol	Catalyses NAD^+^-dependent oxidative decarboxylation, and cholesterol metabolism [100,101]
***TGF-β***	TMEPAI or PMEPA1 or STAG1	TGF-β/Smad/EGFR/Wnt→ TMEPAI→ E3 ubiquitin ligase→ PI3K/AKT → ↓PTEN	Drug resistance via instigating stemness, apoptosis, and EMT [101,102]
***LOX***	*ITGA5*	Hypoxia→ HIF-1α→ ↓miRNA-142–3p→ LOX→ ↑ITGA5/FN1→ pFAK(Y397) & p-Src (Y416)	Chemotherapy drug resistance, collagen crosslinking, fibronectin assembly, and apoptosis [11,103]
***NPM1* or B23**	*PD-L1*	NPM1→ PD-L1→ IL-2→ CD45/CD8^+^/T cells	Ribosome biogenesis, centromere duplication, chromatin remodelling, apoptosis, embryogenesis, and nucleic acid repair mechanism [[104], [105], [106]]

*ADSL*, adenylosuccinate lyase; *AIFM2*, apoptosis including factor mitochondrion-associated 2; *AKT*, serine/threonine kinase; *ASAP1*, ADP-ribosylation factor (Arf) GTPase-activation protein 1; *ATR*, serine/threonine kinase; *B7H3* or *CD276*, biotin or vitamin B7 homolog 3; *BCL2A1*, BCL-2 related protein A1; *BCL2L1*, BCL2 like 1; *BET*, bromodomain and extra-terminal domain like BRD2/3/4 and BRDT; *BRD2/3/4*, bromodomain containing 2/3/4; *BRDT*, bromodomain testis; *BRCA1*, breast cancer A-1; cAMP, cyclic adenosine 3,5-monophosphate; CD28, cluster of differentiation 28; CD45, cluster of differentiation 45 or protein tyrosine phosphatase receptor type C; CD8, cytotoxic T-cell surface transmembrane glycoprotein 8; *Cdc25A*, M-phase inducer phosphatase 1; *CDK*, cyclin-dependent kinase; *CDK6*, cyclin-dependent kinase 6; C-Myc, cellular myelocytomatosis; *CREB*, cyclic adenosine 3,5-monophosphate response binding protein; *CRYβB2*, β-crystallin B2; *CXCL12*, C-X-C motif chemokine ligand 12 or stromal cell-derived factor or chemokine ligand 12 or interleukin 12 (IL-12); *CXCL8*, C-X-C motif chemokine ligand 8; *CXCR4*, cytokine-cytokine receptor 4; *CXCR7*, cytokine-cytokine receptor 7; DNA, deoxyribonucleic acid; *EGFR*, epidermal growth factor receptor; *EglN2*, prolyl hydroxylase EGLN2; EMT, epithelial to mesenchymal transmutation; *EZH2*, enhancer of zeste homolog 2; *FN1*, Fibronectin 1; *FOXC1*, Forkhead box C1; *FUT8*, α-1,6-fucosyltransferase; G2/M, phases of cell division; *GATOR2*, GTPase-activating target of rapamycin 2; G-protein Gα-1, G-protein coupled receptor Gα subunit; H3K27ac, histone 3 lysine 27 acetylation; *HIF*-*1α*, heterodimeric transcription factor 1 alpha; *ICAM1*, intercellular adhesion molecule 1; *ICN1*, interchange cable network 1; *IL1B*, interleukin 1 beta; *IL-2*, interleukin 2; *IL-6*, interleukin 6; *IL-8*, interleukin 8; *ITGA5*, integrin alpha 5; *JAK2*, janus kinase 2; *KTN1*, kinectin 1; *LDLR*, low density lipoprotein receptor; LFA1/Mac1, lymphocyte function-associated antigen 1/macrophane-1 antigen; *LOX*, lysyl oxidase; *MAP3K11*, mitogen-activated protein 3 kinase 11; *MCL*-*1*, myeloid cell leukaemia 1; *MDM2*, murine double minute 2; *MDM4*, murine double minute 2; *MED1*, mediator 1; *MS1*, male-sterility gene 1; *MSN*, moesin; *mTORC1*, mammalian target of rapamycin complex 1; *MUC1*-*C*, mucin 1-cell surface associated; *MYC*, myelocytomatosis protein; *NF-ĸB*, nuclear factor kappa-light-chain-enhancer of activated B; NF-ĸβ/p65, nuclear factor-kappa-β p65 subunit; *NONO*, non-POU domain-containing octamer-binding nucleoprotein; *NOTCH1*, NOTCH receptor 1; *NPM1* or B23, nucleophosmin 1; *NSDHL*, NAD (P) H steroid dehydrogenase-like-protein; p16, protein 16 or INK4A; p21, protein 21; p53, protein 53; p85α, protein 85 alpha; *PD-L1*, programmed cell death ligand 1; pFAK (Y397), phosphor- FAK (tyrosine 397); *PI3K*, phosphatidylinositol 3-kinase; *PIK3CA*, phosphatidylinositol-4,5-bisphosphate 3-kinase catalytic subunit alpha; PKC, protein kinase C; *PMEPA1*, prostate transmembrane protein androgen induced-1; *PTEN*, phosphatase and tensin homolog; Rho C, ras homolog family member C; *RICTOR*, rapamycin-insensitive companion of mTOR; *ROCK1*, rho associated coiled-coil containing protein kinase 1; S/G2, synthesis and G2 phase of cell cycle; *STAG1*, solid tumor-associated gene-1; P-*SMAD3*, phosphor-SMAD family member 3; p-Src (Y416), phosphor-Src (tyrosine 416); *SMAD5*, SMAD family member 5; *SOX9*, SRY-box transcription factor 9; *STAT3*, signal transducer and activator of transcription 3; *TEM8*, tumor endothelial marker 8; *TGF-β*, tumor growth factor-β; *TGFβR2*, transforming growth factor beta receptor-2; *TMEPAI*, transmembrane prostate androgen-induced protein; *TP53*, tumor suppressor protein 53; *TRAF1*, TNF receptor-associated factor 1; *VEGF*, vascular endothelial growth factor; Wnt, wingless integration gene.

Despite addiction genes, driver genes, almost 5798 proteomes [58], and their 3035 specific super-enhancer regions [4] contribute massively to TNBC, for example, moesin (MSN) of the ezrin-radixin moesin (ERM) family serves as cell signal transducer and connective protein among inner cellular membrane, cytoskeleton, and nucleus. The nuclear localization of MSN with protein kinase C (PKC) and phosphorylation activation of threonine at its 558 sites are aided by NONO nucleoprotein. Further, phosphorylated MSN, phosphorylate cyclic adenosine 3,5-monophosphate response binding protein (CREB) that trigger cyclic adenosine 3,5-monophosphate (cAMP), which induce *in vivo* TNBC proliferation and invasion and *in vitro* tumour growth [88]. Some other peptide presented anti-tumor activity and acted as a tumour suppressor; for instance, more CD8 and less CD4^+^ T-cell lymphocytes secreted exosomal programmed cell death 1 (PD-1) checkpoint receptor interrelate either exosomal programmed death-ligand 1 (PD-L1 or B7–H1 or CD274) or cell surface to convince PD-L1 internalization through clathrin-dependent endocytosis that halts clustering of PD-L1: PD-1 by activating *PARP1* (poly (ADP-ribose) polymerase 1) and rescue T cell inhibitory immune checkpoint function in TNBC tumour cells [105,106]. Additionally, the *PARP1* downregulates *PD-L1* transcription through binding at the C-terminal DNA binding domain of nucleophosmin (NPM1 or B23). Therefore, NPM1 enables the binding promoter region of PD-L1, resulting in the inactivation of *PD-L1* [104].

Apart from PD, proteasome ubiquitin pathways play a vivacious role in TNBC management. For instance, BH3-only (Noxa or PMAIP1) has principal significance in neutralizing anti-apoptosis members [107]. The BH3-only bind and neutralize B-cell lymphoma 2 (BCL-2), and BCL-W to regulate Bcl-2-associated X protein (BAX), and Bcl-2-antagonist/killer 1 (BAK1) proteins [108]. Upon activation stimuli, BAK1/BAX homo-oligomerize underneath the mitochondrial outer membrane makes it permeabilise which release intermembrane space protein stimulating caspase-3 and programs cell death [109]. Conversely, myeloid cell leukaemia-1 (*MCL-1*) is an antagonist member of the *BCL-2* apoptotic family that hamper the anti and pro-apoptotic ubiquitin proteomes functional stability by binding to BH3-only to seize BAK1/BAX induction (Fig. 2). The *MCL-1* synthesis is upregulated by epidermal growth factor receptor (*EGFR*), and elongator complex (*ELP*) through the *de novo* signalling pathway [110]. However, *in vivo* aberration/upregulation of *MCL-1* in TNBC carcinoma tissues positively links with extraordinary tumor proliferation and poor patient persistence. Besides, mutated *MCL-1* guards tumour cells from therapy-incited decease and drug resistance [109].

Meticulously, the MCL-1 protein persuaded MS1 peptide that stimulates heterodimeric oncoprotein mucin 1-C (*MUC1*-C), overexpressed 90% in TNBC cells. The cytoplasmic transmembrane C-terminal CQC motif of *MUC1* exhibits oncogenic activity by interrelating diverse effectors and kinases as transforming growth factor (*TGF*)-*β*-activated kinase, while its extracellular N-terminal (MUC1-N) possess conserved glycosylated tandem repeats for its belonging which bind with MUC1-C at the cell exterior and activate BCL-2 related protein A1 (BCL2A1) and Zinc finger E-box binding homeobox 1 (ZEB1) protein via NF-ĸβ p65 signalling cascade. Additionally, ZEB encodes a transcriptional repressor that overwhelms miRNA-200c, downregulates InaD-like protein (*PATJ*), human Lgl polarity *(HUGL2)*, and *CRB3* polarity units, and upregulates BCL2A1 which influence greater epithelial to mesenchymal transmutation in breast cells [111]. Furthermore, MUC1-C provokes forfeiture of epithelial cell polarity and transcription of *DNA methyltransferase* 1 (*DNMT1*) and *3b (DNMT3b)* addiction genes. The *Methyltransferase 1* induces methylation of cadherin 1 *(CDH1)* promoter, which barricades E-cadherin (*E-CDH*), a pivotal proteome that forms adherens junction in healthy cells. During TNBC, MUC1 consign to the mitochondrial outer envelope where it activates oxidative/DNA derestricted stress responses by binding BH3 domain of BAX protein to hinder dimerization of BAK1/BAX and BAX-dependent cytochrome C secretion, thereby inhibiting apoptotic/cell deceased pathway (Fig. 2) [107].

The MUC1-C could crosstalk with receptor tyrosine kinases as WEE1 that regulate G2/M/S phase checkpoint, DNA damage responses, and alter retinoblastoma *(RB)* gene function *via* its three active oncogenic cytoplasmic motifs such as YHPM, YTNP, and CQC motifs. The YHPM motif contains a specific binding sequence for phosphoinositide 3-kinase (PI3K), while PI3K phosphorylation activates serine/threonine kinase (AKT) protein that phosphorylates and deactivates glycogen synthase kinase *(GSK)* 3β and initiate WNT effector β-catenin pathway [112]. Concurrently, tyrosine at the YTNP site of MUC1-C phosphorylated and initiated MUC1-C to SOS linking via interacting growth factor receptor-bound protein 2 (GRB2). Genomic crosstalk of MUC1-C induces RAS → MEK → ERK signalling pathway that propagates signals for TNBC cells mutation, irregular cell division, and proliferation. Additionally, the *PIK3CA*/PI3K signalling pathway is around 40% aberrated in TNBC, where the extracellular matrix stimulation and phosphoinositide 3-kinase (PI3K) generate phosphatidylinositol 3,4,5-trisphosphate PI(3,4,5)P_3_ at the site of the plasma membrane that systematically transformed into phosphatidylinositol 3,4-bisphosphate (PI(3,4)P_2_). The PI(3,4,5)P_3_ and PI(3,4)P_2_ activate diverse kinases, such as 3-phosphoinositide dependent protein kinase-1 (PDK1 or PDPK1) and serum and glucocorticoid kinase 3 (SGK3), that recruit AKT thereby upregulation of Inositol polyphosphate 4-phosphatase type II (*INPP4B*) consort with Rab7 in late endosome/lysosome compartment and dephosphorylate PI(3,4)P_2_ into phosphatidylinositol 3-phosphate (PI(3)P)-effector (HRS) by triggering class-I PI3Kα via PTEN to block EGFR expression, AKT/PI3K signalling, and augment lysosomal GSK3β degradation that inhibits TNBC proliferation and relapse [112]. Similarly, *mTORC1* and *mTORC2* upregulation through *PI3K* prompt *RICTOR* and *GATOR2* that induced violent tumorigenesis in mammary gland, while its pharmacological inhibition by Hippo pathway could be idyllic elucidation (Fig. 2). The hippo signalling pathway is composed of tetra-core, large tumour suppressor 1/2 (LATS1/LATS2), and mammalian Ste20-like kinases 1/2 (MST1/MST2), activated by lack of nutrients, extracellular matrix stiffness, and greater cell density. The FRMD6 and neurofibromatosis 2 (NF2) proteins act over a regulatory sequence of Hippo by phosphorylating tetra-core complex, whereas STING-associated vasculopathy of infantile-onset (SAVI) protein contribute to systemizing MST1 and MST2 [113]. Upon activation, hippo phosphorylates Yes association protein (YAP)/TAZ (also known as WW domain-containing transcription regulator 1) complex at serine on 127 positions of YAP and subjected to subsequent uibiqutinization to prevent further dimerization of YAP/mTOR multiple oncogenic pathways *in vivo* patient-derived xenografts [59]. Thus, the genomic abnormality in PI3K/AKT/mTOR pathway is one of the major issues commonly found in TNBC together with various sub-class of breast carcinoma, causative to tumour progression and tolerance against prevailing treatments [29]. In recent times, targeting the AKT/mTOR signalling pathway is a practical approach for the therapy of TNBC, but its feature *de novo* signalling and action mechanism remain to be elucidated.

The TNBC is the most critical kind of breast melanoma to treat because of its impassiveness against the latest clinical therapies, poor prognosis, high rate of metastasis, and type conversion, such as aberrant *BRCA1* mammary carcinoma is primarily ERα positive but, as they grow, turn into negative or more basal-like TNBC. Furthermore, inherited *BRCA1* carriers have 87% cumulative risk of developing ovarian/breast tumours throughout their lifespan [91]. Additionally, *BRCA1* defected breast express *TP53* mutation in TNBC patients. Mechanistically, germline deficiency of *BRCA1* or *BRCA2* induces multiple tumour proliferation genes such as *TP53* or *TP53BP1* and *NOTCH1* that further activate signalling cascade, ultimately provoking epithelial to mesenchymal transmutation (EMT), and TNBC cell proliferation. For example, the most frequent p53 missense mutation in the 175 codon substitutes histidine with arginine, which creates a steric deterrent that decreases zinc binding affinity around 100–1000 folds, thus causing misfolding of p53 protein [3]. The free-state misfolding of p53 inactivates complementary cell cycle checkpoint via *Chk1* and *WEE1* and regulates EMT, further initiating TNBC cell division [3,114]. Subsequently, p53 interacts with aurora B via the TGF-β pathway while crosslinking with leucine-zipper and sterile-α-motif kinase (ZAK) and CSF1R to trigger EM transmutation in breast tissues. Aurora B has prime importance in terms of mitosis regulation, whereas ZAK kinase regulates the p38 MAPK pathway [95].

Hypothetically, extracellular matrix (EMC), and integrins are the critical players in chemo-immuno resistance during tumorigenesis [11,115,116]. The integrins are heterodimer receptors of the cellular membrane, supposed to rheostat cytoskeletal association, cellular linkage, relocation, and signal transduction through launching focal linkage multiplexes that aid mechanical associations to transfer signals between the intracellular compartment and ECM of the interrelating cells [116]. Thus, the ECM possesses an extremely dynamic erection that is frequently refashioned by cells through improved reassembly, synthesis, chemical adjustment, and degradation [115]. Generally, tumours cells generate an excess amount of ECM like fibronectin 1 (*FN1*), fibroblasts, and collagen. For example, overexpression of ECM enzymes, lysyl oxidase (*LOX*) and matrix metalloproteases, enhance renovation of lysine deposits in collagen prolyl and elastin into extremely responsive aldehydes resulting in cross-linking and equilibrium of ECM's proteins (type I collagen and elastin) that induce TNBC cells motility, invasion and cell adhesion [11].

In addition, transcriptome reprogramming is also one of the perilous features of TNBC, where mutant driver oncogenes or tumour suppressor proteome expression depends on its enhancer or promoter region to initiate tumorigenesis, development, and metastasis [4]. In addition, changes in the TNBC gene expression profile can be due to *cis*-element alteration in noncoding regions where transcriptional factors or other regulatory elements exit [117]. Enhancers are non-transcribe regions of the human genome that possess *cis*-regulatory elements, thereby promoting transcription of the target gene [118]. Generally, it is characterized by acetylation renowned as histone modifications and coactivators association. Super-enhancers are a diverse class of drivers that express a specific set of products that describe cellular individuality [117]. Recently, super-enhancers exhibited a critical character in the overexpression of TNBC driver genes. For example, bromo and extra-terminal domain (*BET*) proteins and their variants such as *BRD2*, *BRD3*, *BRD4*, and *BRDT* are evolving as therapeutic markers for TNBC. The BRD4 and p300 bind with its proximal super-enhancer region enriched in H3K27ac and regulate cell-specifying oncogenic transcriptional plans. However, the direct substitution of *BET* inhibitors with thienotriazolodiazepine (JQ1) enhances acetyl-lysine binding to dislocate BET proteins from chromatin, consequential selective transcriptional reactions in addition to high anti-proliferative efficiency. The discernment of BET obstruction commences from the localization of BETs to super-enhancers that impede anti-tumour impacts in preclinical and clinical tribunals [94]. Epigenomic profiling has been conducted in several solid carcinomas to classify subclass of specific super-enhancers. However, inclusive knowledge related to functional importance in TNBC carcinogenesis of super-enhancers has not been fully established yet [4,118].

### 2.3 Transcriptomes regulation and therapeutics in TNBC

Non-coding RNAs (ncRNAs) are mainly sectioned into short non-coding RNAs (sncRNAs) and long non-coding RNAs (lncRNAs), mostly transcriptome, and are widely occurring in the human genome [119]. The short ncRNAs comprise 20–200 nucleotides in lengths such as micro RNAs (miRNA), small interfering RNA (siRNA), small nuclear RNA (snRNA), small nucleolar RNA (snoRNA), piwi-interacting RNA (piRNA), tRNA-derived small ncRNA (tDR), and also tRNA-derived stress-induced RNA (tiRNA). Whereas, lncRNAs include more than 200 nucleotides in length, such as circular RNAs (circRNA), long intergenic ncRNA (lincRNA), natural antisense transcript (NAT), telomerase RNA component (TERC), and transcribed ultraconserved region (T-UCR) [[120], [121], [122], [123]]]. The protein-coding RNAs are generally 3%, while the rest of the genome is occupied by dark matter that can frequently code ncRNAs [124,125]. In recent times, incipient evidence recommended that the ncRNAs play a pivotal role in developing TNBC, presented briefly in Table 2.

**Table 2:** Role of transcriptomes in the regulation of triple-negative breast cancer proliferation and metastasis.

Transcriptome	Function	Target gene
Short noncoding RNA		
1. Micro RNA		
miR-let-7a-5p	Sustaining proliferative signalling	Upregulate *RRM2* [126]
miR-let-7b-5p	Induce tumorigenesis	Upregulate *CCNA2* [126]
miR-let-7e-5p	Induce tumorigenesis	Downregulate lncRNA-IGF1 [126]
miR-10b-3p	Induce tumorigenesis, activate invasion, and metastasis	Upregulate *CCNA2* [126]
miR-18b-5p	Induce tumorigenesis, promote migration, invasion, and increase cisplatin resistance	Downregulate lncRNA-IGF1 and lncRNA-ESR1 [126]
miR-19a-3p	Induce tumour cell immortal replication	Downregulate lncRNA-ESR1 [126]
miR-22–3p	Suppress tumour cell proliferation, colony formation, migration, and invasion	Downregulate *eEF2K* [127]
miR-29b2/29c	Suppress tumorigenesis, migration and metastasis, EMT, and mammary gland development	Regulate *TGIF*, *CREB5*, and *Akt3* [[128], [129], [130], [131]]
miR-30a	Inhibit EMT, drug resistance, and tumour cell migration	Downregulate *ZEB2*, *IGF2*, and *IRS1* [132,133]
miR-34a	Suppress EMT, invasion, proliferation, migration, breast cancer stem cells, increase dasatinib sensitivity, and induce M1 macrophages	Downregulate *NOTCH* synthesis, and upregulate IL-6R antibody [[134], [135], [136]]
miR-98–5p	Induce tumorigenesis	Downregulate lncRNA-IGF1 [126]
miR-106b-25	Induction of tumour-initiating cells (TIC), EMT	Downregulate *NEDD4L*, upregulate *NOTCH1*, and activate *TGF-β* signalling [137]
miR-124	Suppress tumour and rescue evading immune destruction	Downregulate *ZEB2* [138]
miR-128–3p	Induce proliferation, regulate glucose metabolism, and post-transcriptional regulation of genes,	Downregulate *IRS1* [133,139]
miR-130b-3p	Suppresses tumorigenesis and increases doxorubicin sensitivity	Downregulate lncRNA-IGF1 [126]
miR-142–3p	Chemosensitization and reduce metastasis	Downregulate *FN1*, *ITGα5*, and *LOX* [11]
miR-146a-5p	Induced invasion and migration of tumour cells	Upregulate lncRNA HOTAIR [140]
miR-199a	Induce tumour cell proliferation, stemness, and EMT	Regulate Akt/mTOR signalling pathway [141,142]
miR-205–3p	Suppress metastasis, migration, invasion, stem cell-like property, modulate cell-cell adhesion, and cell cycle progression	Downregulate *ITGα5*, target *E2F1*, and LAMC1 [143]
miR-214	Induce tumour cell proliferation, stemness, and EMT	Unknown [141]
miR-222–3p	Evading growth suppression and luminal form of TNBC progression	Downregulate lncRNA-ESR1 [126]
miR-325–3p	Induce tumour progression	Upregulate mutant p53 expression [144]
miR-410–3p	Induce tumour progression	Upregulate *CCNB1* [126]
miR-637	Suppress tumour cell migration, and lymph node metastasis in TNBC	Downregulate Akt1, β-catenin and cyclin D1 [145]
miR-3178	Suppress tumour cell proliferation, invasion, migration, and metastasis	Downregulate Notch1, and *ETV1* [146]
2. Micro RNA Sponge		
circR-PSMA1	Tumorigenesis, migration, metastasis, and immunoresistance	Sponged miR-637, upregulate Akt1 [145]
lncR-HNF1-AS1	Induce tumorigenesis but decrease apoptosis	Sponged miR-32–5p, upregulate RNF38 [147]
lncR-WEE2-AS1	Induce tumorigenesis	Sponged miR-520f-3p, upregulate *Sp1* [148]
lncR-PITPNA-AS1	Induce tumour cell viability, proliferation, migration, and invasion	Sponged miR-520d-5p, downregulate DDX54 protein, Upregulate *SIK2* [149]
lncR-SNHG11	Induce tumour cell proliferation, migration, and decrease apoptosis	Sponged miR-2355–5p, downregulate *CBX5* [150]
lncR-PART1	Induce proliferation, migration, mammosphere formation, and stem cell-like property	Sponged miR-937–5p, target myosin-Va, *ZHX2*, and regulate miR-190a-3p, miR-22–5p, miR-30b-3p, and miR-6870–5p [151]
lncR-HAGLR	Induce proliferation, cell viability, migration, and invasion	Sponged miR-335–3p, and upregulate *WNT2* [152]
Long noncoding RNA		
lncR-MIR22HG	Impair TNBC proliferation and invasiveness	Downregulate *c-Myc* [89]
lncR-MaTAR25 or LINC01271	Destruction of the actin cytoskeleton, augmentation of microvilli, focal adhesion, mammary tumour cell proliferation, migration, and invasion	Upregulate *Tns1* [153]
lncR-049808	Induce tumour proliferation, migration, and invasion	Upregulate miR-101, and *FUNDC1* [154]
lncR-PDCD4-AS1	Suppress tumorigenesis, migration, invasion, and increase apoptosis	Downregulate miR-10b-5p, and upregulate IQGAP2 expression [155]
lncR-PAPAS	Induce tumour cell growth, migration, and invasion	Downregulate miR-34a, and upregulate lnc-OC1 [156]
lncR-RMRP	Induce tumour cell growth, migration, and invasion but decrease apoptosis	Upregulate miR-766–5p [157]
lncR-005620	Induce doxorubicin or epirubicin resistance	Target *ITGβ1* [158]
lncR-GAS5	Suppresses tumorigenesis, activate tumour suppressor genes, control signalling pathways such as PI3K/AKT/mTOR, Wnt/β-catenin and NF-ĸβ signalling, and multi drug sensitivity	Downregulate mir-21, mir-222, mir-221–3p, mir-196a-5p, mir-378a-5pE2F1, *EZH2*, and *YAP*; upregulate *PTEN*, *PDCD4*, *DKK2*, *FOXO1*, and *SUFU* [159,160]
lncR-TUSC7	Suppresses tumour growth, increase carboplatin, and paclitaxel sensitivity	Upregulate miR-1224–3p, and genes of cell cycle pathways for example TGF-ss, and *BUB3* [161]
lncR-AFAP1-AS1	Induce tumour progression, migration, and invasion	Downregulate miR-2110, and upregulate *Sp1* transcription factor [123]
lncR-TROJAN or AK124454	Promotes TNBC progression and invasion	Degradation of ZMYND8 repressor [162]
lncR-NUDT3-AS4 or AL354740.1–204	Induce aggressive TNBC pathology	Enhance Akt1/mTOR expression [142]
lncR-NEAT1	Enhance radioresistance, proliferation, cancer stem cells, stemness genes like *OCT4*, *BMI1*, and *SOX2*	Induced *NQO1*, and downregulate miR-98–5p [126,163]
lncR-MAL2	Induce tumorigenesis	Upregulate miR-let-7b-5p, and mir-410–3p [126]
lncR-MALAT1	Chemoresistance, angiogenesis, and oxidative phosphorylation canonical	Upregulate lncR-NEAT1 [164]

*BUB3*, budding uninhibited by benzimidazoles 3; *CBX5*, chromobox 5; *eEF2K*, eukaryotic elongation factor 2 kinase; EMT, epithelial-mesenchymal transmutation; *FN1*, fibronectin 1; *IGF*, insulin-like growth factor-1; IL-6R, interlukin-6 receptor; *IRS1*, insulin receptor substrate-1; *ITGA5*, integrin alpha 5; *ITGA5*, integrin α5; lncR-*PART1*, long noncoding RNA prostate androgen regulated transcript 1; *LOX*, lysyl oxidase; *NQO1*, NAD(P)H: quinone oxidoreductase 1; RNF38, ring finger protein 38; *Sp1*, specific protein 1; *TGF-β*, targeting transforming growth factor beta; *Tns1*, Tensin1; *ZHX2*, zinc fingers and homeoboxes protein 2.

The fundamental factor behind the demise of TNBC patients is neurons-based metastasis and EMT. The EMT is devoted to the commencement of tumour metastasis in TNBC that cause impairment in cellular adhesion junctions. However, EMT has retained a highly complex genetic mechanism elicited *via* discrepant intracellular and extracellular transcriptomes and proteomes signalling that foment carcinoma cells to decamp their prime location [165]. Subsequent eruption into the oblivious organ, mesenchymal TNBC cells transform into epithelial cells to relinquish macro metastatic foci. Usually, the ncRNAs have the chief regulation in terms of *in-vivo* genetic expression, cell proliferation, protein synthesis, transcript targeting, and activation of receptors [119]. However, abnormal behaviour of various classes of ncRNAs has been perceived in TNBC that are discussed in detail [120].

#### 2.3.1 Short noncoding RNAs modalities

##### 2.3.1.1 Micro RNA

Micro RNAs are composed of 20–24 nucleotides that are involved in gene regulation. Recently, they have been used in the diagnosis and advanced gene therapy-based therapeutic potential of TNBC [126]. The miRNA also exhibits anticancer responses without generating reactive confrontation [166]. It tends to express innumerable countenance configurations among TNBC patients and other classes of breast carcinoma [119]. Additionally, miRNAs intimate carcinoma development and screen the prognosis in TNBC patients. Exclusively miRNA has the ability to suppress tumours *via* regulating EM transmutation in TNBC [141]. For example, wild-type p53 directed transactivation of miRNA of 205 family (miR-205). The miR-205 inhibits EM transmutation, relocation, incursion, and propagation by down-regulating Zinc finger E-box binding homeobox 1 (*ZEB1*) gene, integrin α5 (*ITGA5*), *E2F1*, *LAMC1*, and ITGA5/Src/Vav2/Rac1 signalling pathway where integrin α5 is the widely occurred heterodimeric transmembrane receptor for extracellular proteome of the cell that plays a pivotal role in cell to cell adhesion and TNBC metastasis [143], while *E2F1* govern cell cycle and *LAMC1* systematize cell adhesion and metastasis. Similarly, miRNA-199, miRNA-214, and miRNA-3178 inhibit EMT alteration [141], TNBC cell proliferation, invasion, and metastasis by direct downregulation of the *NOTCH1* gene [146] that is supposed to be a key driver in terms of tumour cell initiation signalling. The tumour initiation cells (TIC) have a potent ability to reseed carcinoma in distinct places in TNBC patients [137].

In addition to positive feedback, numerous miRNAs elicit negative regulation. For instance, the miRNA-106b-25 group mediates breast carcinoma initiation by inducing Notch1 protein and unambiguous negative feedback inhibition of NEDD4L, the E3 ubiquitin ligase [137]. This induction/inhibition initiates *TGF-β* signalling and EMT transmutation in TNBC [141]. Likewise, miRNA-374a-5p is perceived to be highly upregulated in TNBC patients by downregulating the arrestin beta 1 (*ARRB1*) gene, while *ARRB1* upregulation significantly overcomes TNBC worse prognosis [167]. Additionally, the polycistronic oncogenic miRNA-17/92 and miRNA-106b/25 interrelate with various signalling cascade and tumorigenic effectors and inhibit the translation of essential proteomes in TNBC metastasis [141].

Recently, diverse targeting of miRNA has been identified in TNBC poor prognosis and metastasis, for instance, the noncoding single nucleotide polymorphism rs78378222 in the sequence of AATACA, instead of wild-type AATAAA in *TP53,* generates a targeting site for miRNA-382–5p or miR-382 that in turn provide the site for miRNA-325–3p (miR-325) which is greatly overexpressed in the mammary glands. The unusual behaviour of both miRNA cause upregulation of p53 in breast tumours and sealed neuro-carcinoma proliferation in the brain sites [144]. Also, miRNA-17–5p rheostat TNBC metastasis through suppressing *ETV1* oncogene [168], E2F transcription factor 6 (*E2F6*), and DNA methyltransferase 1 (*DNMT1*) expression while overexpressing *BRCA1* in TNBC, thus proved to be a differential role of miRNA in TNBC suffering [169]. *E2F6* and *DNMT1* serve as transcriptional precursors and regulators of cell proliferation and its fate [130,169].

##### 2.3.1.2 Short interfering RNA

Ribonucleic acid interference is a natural phenomenon mainly achieved by siRNA and short hairpin RNA (shRNA), which can ideally complement a specific target gene or miRNA for silencing [5,170]. However, the siRNA can be synthetic and natural, whereas the endogenous double-stranded ribonucleic acid (dsRNA) is typically renowned by a molecular scissor named as dicer, which sliced dsRNA into 20–25 nucleotide pairs in length. These short interfering RNA having two bases overhang at their 3’ site [171]. Recently, the underlying mechanism of RNAi has been unveiled where some chromatin-binding RDM15 protein-directed polymerase-V dependent activation accumulation of siRNA at RNAi target site and RNA-directed deoxyribonucleic acid methylation occurred *via de novo* methylation pathway. The special hydrogen bonding and aromatic cage at the C-terminus of the Tudor domain of RDMI5 precisely identify the monomethyllysine of active histone H3K4me1 peptide and initiate RNAi in living beings [172]. Therefore, the siRNA is a presumptive knockdown gadget to inhibit the genetic expression profile of any gene and explore distinct molecular cascades and genetic/metabolic routes at the posttranslational level [173]. It proposes an innovative therapeutic approach to deal with several disorders or diseases, including TNBC and various other melanomas at the biomolecular level.

Several siRNA depended target therapeutic strategies have been employed to cure TNBC, such as *TGF-β* expression level modulated with siRNA-based interference that exhibited an efficient decline in metastatic condition since *TGF-β* involves in cell fate determination, differentiation, apoptosis, and has the ability to interrelate with cell cytoskeleton, integrin's, and epithelial cadherin's based relocation during TNBC metastasis [5]. Similarly, the vascular endothelial growth factor (*VEGF*) family, such as *VEGF-A* to *VEGF-D* and placenta growth factor (*PEG*) expression, have been successfully meticulated by *VEGF* siRNA (5’-GCUACUGCCAUCCAAUCGAtt-3’) that were highly expressed in TNBC cells, and significantly correlated with worse metastasis, thus enhancing pro-angiogenic impact over endothelial cells, and facade as autocrine for VEGF receptor and survival [174].

Some shRNA [175] and siRNA [10] overturn the biosynthesis, bioenergetics, and downregulate aerobic glycolysis which is the primary necessity of TNBC carcinoma cells. Notably, *SLC2A1* encoded glucose transporter 1 (*GLUT1*) influences Rb tumor suppressor 1 protein, which is generally upregulated and incites the progression of basal-like TNBC, the crucial rate restraining factor in terms of glucose uptake and metabolism dependencies [176]. Mechanistically, the pro-apoptotic death effector domain-containing (DEDD) peptide is found to be 60% overexpressed in the TNBC cell's nucleus and cytoplasm. This DEDD peptide crosstalk with 71 ​kDa heat-shock 8 chaperons that accelerate the manifestation of cyclin D1 while mortifying RB1 protein to empower G1/Synthesis phase dysregulation aggravates sensitivity against *CDK4/6* and *EGFR* treatments in TNBC [177]. In this context, the siRNA-based whole-genome screen provides potential elucidation [178]. However, the polar and macromolecular landscape of siRNA deters its cellular admittance to employ its impact [120].

#### 2.3.2 Long noncoding RNAs modalities

The therapeutic targeting of ncRNAs signifies a striking tactic for the therapy of TNBC, as well as many other melanoma and diseases [166]. During the last decade, extensive exertion has been through concerning the clinical application of ncRNA-based therapeutics, employing mostly RNA interference liable techniques, with numerous attaining food drug and administration (FDA) and European medicines agency (EMA) endorsement [120]. However, practical consequences have thus far been indecisive, with some reports noted persuasive impacts while others confirmed limited efficacy or cytotoxicity [123]. Different entities, for instance, lncRNA-based therapeutics are achieving inquisitiveness [153]. The lncRNAs about 200 nucleotides in length are polyadenylated RNAs transcribed by RNA polymerase-II [163], play a part during epigenetic trigger gene expression, steric interruption of secondary structure realization, miRNA silencing, regulation of cell cycle, post-transcriptional inhibition, protein interaction, and signal transduction cascades [179].

More than 9500 transcripts of lncRNAs dysfunction mainly triggered proliferation, epithelial to mesenchymal transmutation, apoptosis, and other pathological progressions *via* the diverse mechanism in TNBC patients [180]. Also, the comprehensive transcriptomic expression inspection from 1097 breast carcinoma patients, categorized about 1510 lncRNAs differential expression among TNBC. However, 672 lncRNAs were found functionally abnormal between non-TNBC and TNBC individuals [181]. Technically, in the human genome, anomalies are related to the lncRNA actin fibers associated protein 1 antisense RNA 1 (lncRNA-AFAP1-AS1 or AFAP1-AS1) positioned at the anti-sense chain of *AFAP1* gene on chromosome 4, closely associated with breast tumour proliferation and metastasis. However, 25–50% knockout of (ENSG00000272620) *AFAP1-AS1* is supposed to be adequate to diminish propagation and colony establishment of MDA-MB-468 and MDA-MB-231 ​cells *in vitro*. Meanwhile, *AFAP1-AS1* dependent silencing leads to the inhibition of BT-549 and MCF-10A cell development and relocation of simultaneous degradation of *Sp1* transcription factor and septin-2 (*SEPT2*) *via* upregulation of miR-2110 and miR-497–5p [123,182]. Additionally, it induces EM transmutation by the Wnt/β-catenin signalling pathway during TNBC relapse [183,184]. Similarly, overexpression of lncRNA NEAT1 induce by hypoxia enzyme NAD(P)H: quinone oxidoreductase 1 (*NQO1*), enhances rapid division in MDA-MB-231 and Hs578t cells by downregulating miRNA-193b-3p and upregulating cyclin D1, and stemness genes: *OCT4*, *BMI1*, and *Sox2* [163]. Also, the genome-wide transcriptomic data from TNBC confirmed the potential prognostic existence of primate lncRNA (TROJAN) that degrade zinc finger MYND-type 8 (*ZMYND8*) *via* ubiquitin-proteasome pathway while repelling zinc finger protein 592 (ZNF592) [162].

Some other TNBC biomarkers comprehend exosomal circPSMA1 provoke cells proliferation, metastasis, and relocation in tumor-infiltrating immune cells *in vitro* and *in vivo* through miRNA-637/Pl3K-Akt1/-catenin (*cyclin D1*) pathway [126]. Mechanistically, circPSMA1 fascinated miRNA-637 and augmented TNBC cellular metastasis through direct victimized *Akt1*, renowned as a crucial immune-related gene and targeted downstream *β-catenin* and *cyclin D1* [145]. Similarly, lncRNA AL354740.1–204 (NUDT3-AS4) binds with sequence and elevates the synthesis of the miRNA-99s family comprised of miR-100, miR-99a, and miR-99b. The inordinate accumulation of miRNA-99s complement with Akt1/mTOR mRNA and is directed to degradation, decreasing the expression of Akt1/mTOR protein of pathway [142].

Additionally to the transcriptional directives, lncRNAs, have other governing characters [179]. For instance, the long noncoding mammary tumour-associated RNA25 (MaTAR25) downregulated Tensin1 (*Tns1*) protein in 4T1 cells by interacting with purine-rich element binding protein B (PURB) and heightened tumor cell progression. Inhibition of Tns1 protein exhibits reorganization of actin filaments, weakening of focal adhesion association signalling between extracellular matrix and cytoskeleton, and microvilli [153]. Correspondingly, lncRNA DILA1 can hinder the phosphorylation of cyclin D1 by intermingling Thr286 to stop cyclin D1 deprivation while elevating breast metastasis [185]. However, comprehensive characterization of the action mechanism of most of the lncRNAs for molecular occurrence and precise function in TNBC still needs to be explored.

## 3 Ongoing treatments for triple-negative breast cancer

**Fig. 3:**
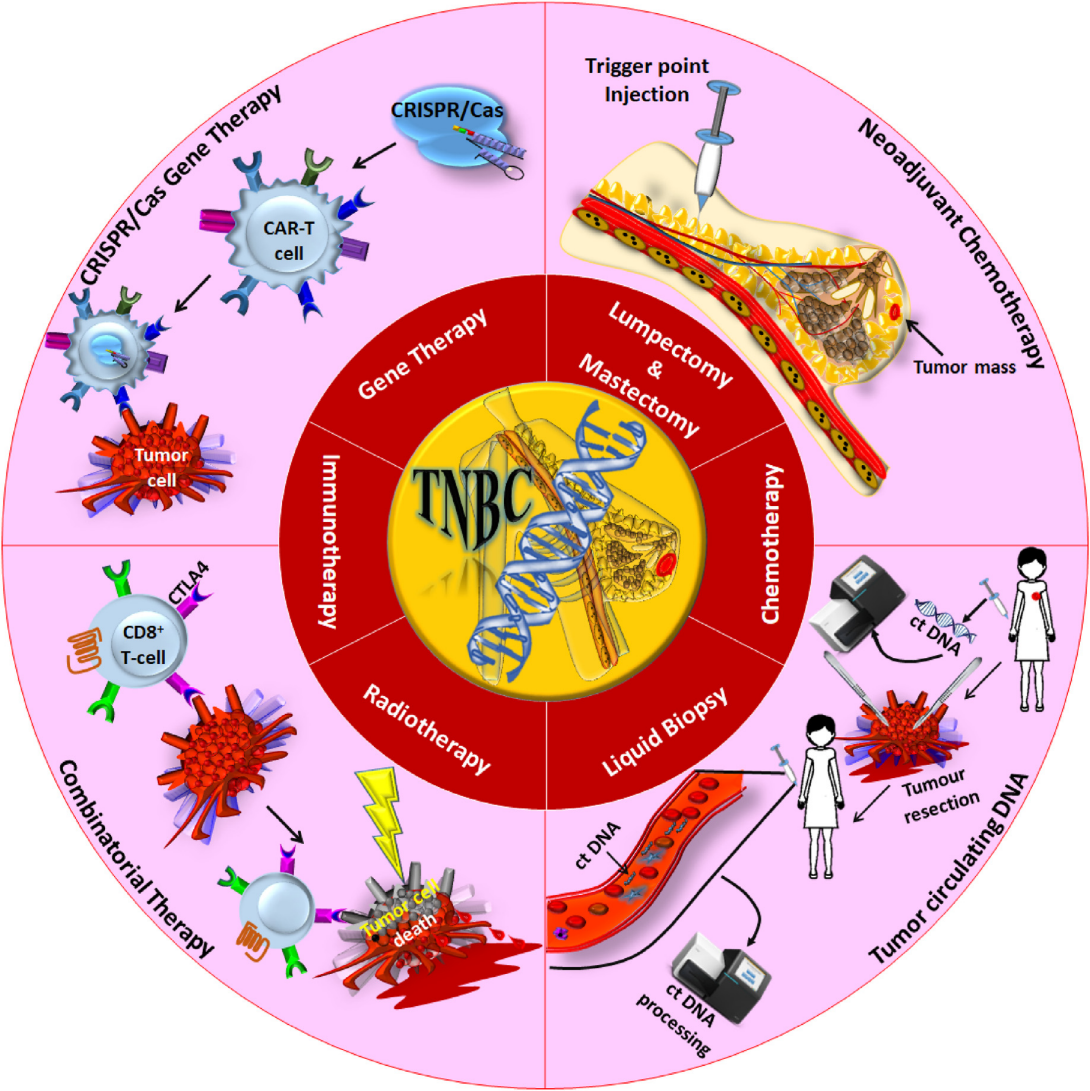
Overview of triple-negative breast cancer traditional and advanced therapeutic applications. Over time, six traditional techniques were widely utilized to overcome breast carcinoma or TNBC (inner ring), while the outer ring indicated their modified mechanics with recent advancements. As discussed in the review, lumpectomy, mastectomy, and chemotherapy have been utilized as first-line treatment, while others are used as advanced treatments to relocate and treat relapse and metastasis in clinical translation.

Multiple approaches are applied to treat TNBC, such as conventional (adjuvant and neoadjuvant chemotherapy), surgery, endocrine therapy, and radiation therapy. While, advanced strategies include active and passive targeting by gene manipulation techniques (CRISPR, siRNAs, miRNAs, and lncRNAs), and nanocarriers-based therapies, which prevent the spread of the tumour and eliminate the chances of recurrence of TNBC, are discussed in the sub-sections with detail and illustrated in Fig. 3.

### 3.1 Lumpectomy and mastectomy

Presently, there are no precise strategies for the orthodox treatment of TNBC due to the heterogeneity of this disease depicting varied compulsive structures. However, the initial treatment of TNBC generally depends on the surgery that is divided into two types; one is lumpectomy and the other is mastectomy [186]. The lumpectomy is mainly conducted to preserve the breast, especially when TNBC is at its infancy [187], while mastectomy approaches involve the removal of the entire breast to save the patient's life. It is commonly done when the disease recognizes at the last stage or after chemotherapy. Nonetheless, both surgeries induce 25–60% post-pain syndrome (PPS) in women due to the damage of T4 and T5 peripheral nerves during surgery, ensuing neuroma development along with hypersensitivity [188]. Generally, intercostal T4 and T5 nerve sensory branches exhibited major chronic neuropathic pain aetiology as they supply blood from the chest wall to the breast. Further, during surgery, an acute injury in the sensory part of cutaneous nerves leads to neuroma development and axonal cell membrane successive growth that progresses pacemaker-like commotion causing incessant devastating neuropathic pain syndrome accompanied by poor life quality [189]. It could be obstinate, stimulus sensational agony, and trigger intensified pressure at the position of the neuroma. However, PPS is hardly treated clinically or documented by surgeons. Although, the majority of TNBC patients do not obtain effective treatment even after diagnosis due to a lack of targeted medication. While, some standard medications of breast carcinoma provide ease to relieve pain for a certain period of time, such as non-steroidal anti-inflammatory drugs, corticosteroids, opioid analgesics, interleukin-1β, interleukin-6, interleukin-10, benzodiazepines, gabapentin, antidepressants, pectoral nerve block-I, and serratus anterior plane block [188,190], usually express forged consequences in TNBC.

Some advanced trigger point injections (TPI) are somehow getting interest in suppressing chronic pain. The functional principle of TPI is to inject 2 ​mL of 50 part 4 ​mg/mL dexamethasone and 50 part of 0.5% bupivacaine at the site of neuroma that alleviates 91.2% of relatively long-term PPS [189]. Also, perineural infiltration of anaesthesia in combination with dexamethasone or dexamethasone and bupivacaine at the spot of the supposed neuroma showed dormant outcomes [191]. Thus, anaesthetic treatment only affords brief-time relief, nevertheless it supports the diagnosis. However, ongoing strategy induces vilest chronic neuromas by augmenting the pacemaker activity, which consequences in either short-term or rapid PPS relief enforcing effective therapy for TNBC cure and patient ease.

### 3.2 Chemotherapy

Before and after lumpectomy or mastectomy procedure, some undetected deposits in the breast tissues or lymph nodes may lead to relapse in TNBC. However, chemotherapy is one of the most widely practiced conventional first-line short-term therapy to cure post-consequences of TNBC surgery that was introduced in clinics almost half of decades [192]. It has diverse administration modes, such as oral chemotherapy, intramuscular, subcutaneous, intravenous, intrathecal chemotherapy, and types like single agent adjuvant and neoadjuvant chemotherapy (NAC) [193]. The single or multiple agent adjuvant chemotherapy is based on two factors, one being predicted sensitivity concerning a specific methodology and its allied benefits and another being the risk of relapse. Further, implementing any method depends upon the TNBC patient's ailment [194]. The NAC has endorsed surgery formerly to study the impact of medicine in TNBC patients and lessen the magnitude of lumpectomy or mastectomy if patients respond to the chemo-therapeutic dose [193]. Principally, chemotherapeutic agents kill not only tumour cells but also healthy cells. Most accessible treatments may provide anticipated antitumour impact at higher quantities of the alkylating agent, plant alkaloids, antimetabolites, and antitumour antibiotic [195], which is highly hazardous to healthy cells. Further, these cytotoxic chemo-drugs only increased 33% overall survival rate with adverse anemia in TNBC patients [196]. Other after-effects of chemotherapy agents include sickness, fatigue, rash, diarrhea, leukopenia, alopecia, thrombocytopenia, abdominal discomfort, vomiting, peripheral sensory neuropathy, hepatic failure, auto immuno hepatitis, constipation, pneumonia, and fluctuations in weight [15,195,197].

Concisely, among adjuvant, preparative, and neoadjuvant chemotherapies, the NAC is more effective with an advanced ratio of pathologic complete response (pCR), averts tumor relapse in TNBC-bearing patients as compared with non-TNBC patients [193,198]. However, the earlier analysis indicated a higher rate of reversion and distant metastasis during the first 3 years after neoadjuvant chemotherapy [9]. Recently, platinum-based chemotherapies are becoming a new option for solid carcinomas. On the other hand, a high ratio of platinum-based salts exhibits more BRCA-like status in TNBC patients. For example, platinum-based adjuvant chemotherapy (AC) and NAC trials of about 15 studies prove a higher toxic impact that develops worst outcomes after recurrences. Additionally, it induces severe neuropathy and thrombocytopenia in all grade patients, followed by neutropenia. Also, it has only enhanced disease-free survival (DFS) but not overall survival (OS) response (HR ​= ​0.98, *p* ​= ​0.87; CI ​= ​0.75–1.27, 95%). Therefore, it was suggested that this chemotherapy could not be a better option as a first-line treatment for TNBC patients [196]. Similarly, in another randomized trial (NCT00861705, CALGB 40603) with stage II and III TNBC patients (*n* ​= ​443), the used drug such as Carboplatin and Bevacizumab along with Paclitaxel, followed by Cyclophosphamide and Doxorubicin in NAC setting did not improve long-term OS. However, Cyclophosphamide and Paclitaxel are considered standard therapy for TNBC [199]. Thus, the new therapy is an unmet need to increase the life span of TNBC patients.

### 3.3 Liquid biopsy or circulating tumour DNA

The TNBC manifests a higher incidence of relapse and visceral metastasis during the first 36 months after chemotherapy and shortened life span. However, a substantial number of TNBC interventions receive NAC and an unambiguous dichotomy exists in consequence-centered retort to NAC. Almost 13of TNBC interventions will accomplish 94% pCR with a promising disease-free OS for 3 years. In contrariety, 23of TNBC interventions exhibit lingering ailment after NAC and are at an extreme hazard of 68% metastasis during 3 years of prediction. Thus, advancements have been subjected to locate the deposition of tumour substantial in the circulatory system of TNBC patients, who are supposed to be disease-free either lumpectomy or mastectomy after NAC. An incipient scheme for non-invading tumour discernment is the examination of circulating tumour DNA (ctDNA) or liquid biopsy and circulating tumour cells (CTCs) to reveal the status of treatment efficacy and tumour prognosis in patients [200]. In a liquid biopsy, the ctDNA is unfettered in the bloodstream to sense CTCs that are usually released in the blood at the site of metastatic lesions or primary tumour cells undergoing necrosis and apoptosis [201]. For most, the fraction of ctDNA matched to the whole cell-free nucleic acid (cfDNA) that would be in a minute quantity of <1%. Lately, thoughtful droplet-based digital polymerase chain reaction (ddPCR), and next-generation sequencing techniques are used to analyse such a minute quantity of ctDNA. However, this blood-based prognostic biomarker in its infancy needs a large prospective study to validate its fruitfulness after NAC in high-risk TNBC intervention population [202].

### 3.4 Immunotherapy

The T-cell is one of the white blood cells that have a central role in cancer immunotherapy. It is a growing field of advanced therapy in which cytotoxic T-cells (CTc) directly act on the microenvironment of tumour that causes minimum after-effects on normal cells. The tumor-specific adaptive immune response controls tumor growth by stabilizing the long-term memory of adaptive T-cells to achieve a possible lasting cure. Mechanistically, activation of CTc initiates anticancer immuno retort by exhibiting expression for immune checkpoint inhibitors such as PD-1 and cytotoxic T-lymphocytes antigen 4 (CTLA-4) [192]. Importantly, PD-1 is a cell surface protein expressed on tumour-infiltrating lymphocytes (TILs) that has been widely studied in TNBC immuno treatments [192,193]. Moreover, The non-self-cells secretion upon genomic mutation actively identified *via* PD-1 and CTLA-4 leads to tumour cell elimination that ends after overcoming through immune escape process initiated by tumour cells either PD-L1/L2 or downregulating MHC-I/II [18,203]. In recent times, immunotherapy-based drugs, more likely antibodies, proved to be a promising antidote than chemotherapeutic induction alone due to immuno-responsive nature of TNBC [193].

In this context, exogenous cytokines-based vaccines (that enhance the rate of tumour-specific T-cells), adoptive T-cells, adaptive cells, several immune checkpoint inhibitors, chimeric antigen receptors (CAR) T-cells and co-stimulatory receptors have been greatly explored to overcome immune-suppressive response under tumour microenvironment and decrease TNBC burden in women [35,104,106,[203], [204], [205]]. However, there are various limitations in which unpredictability of method outcome is the biggest. For instance, only a few subsets of tumour patients exhibit immune responses, while others relapse after initial retort. Further, different kinds of tumour respond variably, maybe due to the presence of their own intrinsic resistance/tolerance and immunosurveillance mechanism [206]. In addition to the method, it is extremely expensive. For example, advanced immunotherapy costs about 1 million US $/visit, which creates an unbearable burden on average for mediocre earner patients, and psychology also weakens the immune system. Other limitations regarding immunotherapy are tumour heterogeneity, tumour microenvironment (TME), paucity of adequate neoantigens, insufficient production and malfunction of tumour-specialized CD8 T-cells, and epigenetic alteration resulting inefficient activation of tumour-specialized immune retort [204,206,207].

Apart from this, several pre-clinical and clinical trials are assessing to establish chemo-immuno therapy regimes. For example, NAC coupled with immunotherapy evaluated the efficacy and safety of combinatorial treatment and Durvalumab administration in I-III stage *PD-L1* positive TNBC patients (*n* ​= ​59). Additionally, Pembrolizumab (Keytruda, MK-3475), Atezolizumab, or Durvalumab are first-line treatments in *PD-L1* positive TNBC patients. The Durvalumab was provided with nab-Paclitaxel followed by dose-dense Cyclophosphamide and Doxorubicin (ddAC) where 44% pCR was reported from Phase-II together with 31% adverse events such as anemia and neutropenia. In addition, Guillain-Barre syndrome (1.9%), hyperglycaemia (3.9%), colitis (3.9%) and death (1.9%) were also observed [208]. Likewise, the COLET Phase-II trial evaluated Cobimetinib in combination with Paclitaxel, or placebo combined with Paclitaxel in cohort-I, Cobimetinib along with Paclitaxel and Atezolizumab in cohort-II, and Cobimetinib together with Atezolizumab and nab-Paclitaxel in cohort-III, did not demonstrate an advantage in relapse free survival (RFS) as first-line therapy for metastatic TNBC (mTNBC) irrespective of MAPK/MEK and PD-L1 [20]. Further studies are ongoing to comprehend the unmet need for chemo-immunotherapy drugs better.

### 3.5 Radiotherapy

Radiation or radiotherapy has exhibited fewer after-effects than chemotherapy in breast tumour vulnerable populations [38]. It is practiced to execute residual tumours residing either in the breast or lymph node to device relapse of breast carcinoma [209]. It is mainly sectioned into external radiation beam that is traditional or whole breast radiotherapy with highly focused x-ray beam for 2–3 ​min as many as five days in a week [113] with a total dose of about 4500–5000 ​cGy (cGy) for 5–7 weeks followed by 1 week of booster dose of 1000–2000 ​cGy [210]. However, treatment doses may vary from patient to patient and the type of radiotherapy. Other kinds of radiotherapy include internal breast radiation or brachytherapy [113]. It is an advanced technique to deliver radioactive liquid compounds via injection at the site of affected tumour tissues, which is supposed to activate the expression of CD4^+^ and CD8^+^ T cells in the presence of neoantigens triggered by somatic *de novo* mutation. Specifically, neoantigens-dependent CD8^+^ T cells easefully targeted irradiated tumour tissues, while CD4^+^ T cells involved in manipulating Th1 cytokines and neoepitopes spread. In advance, genes expression rely upon radiation intensity which upregulated death receptors FAS/CD95, DRS and MHC-II molecules on the plasma membrane of the carcinoma cells [205,211]. On the contrary, it is believed that those patients who have failed to stimulate T cells-based anti-tumour activity, showed shorter life spin during pathogenesis. Further, some antagonist-genes/proteome like myeloid-specific immune checkpoint CD47 protein bind to its SIRPα ligand that is located on the macrophage's surface, thereby phosphorylating the cytoplasmic tail of CD47 ligand to mediate the anti-phagocytic signals to limiting macrophages ability and inducing radio-resistance in the tumour-bearing breast [38].

Although recent advancements that favoured mastectomy combined with adjuvant radiotherapy [205] or immunotherapy along with radiation [212], yield somehow favourable outcomes for internal lymph node melanoma in TNBC. Further, the late hypofractionated accelerated radiotherapy with the dose of 50–52 ​Gy, which was assumed to be relatively safe for breast carcinoma treatment, resulted in 2.2% symptomatic breast oedema, 1.9% asymptomatic fibrosis, 2.2% contralateral breast, 3.7% arm lymphedema, and 1.6% other melanoma in 290 investigated patients during amifostine abridged primary radiotherapy dermatitis [213]. However, radiation therapy could only recover the prognosis risk in the low-risk TNBC population (P ​= ​0.00056), and revealed non-significant betterment in medium and high-risk patients with short existence duration and mortality within 5 years from diagnosis due to the acquirement of radiotherapy tolerance [214]. Moreover, radiotherapy impairs healthy cells surrounding by tumour cells, induces a rare tumour named angiosarcoma, heaviness and swelling in the breast, numerical/copy number alteration and structural chromosomal aberrations, and infertility [210]. Therefore, applying more advanced technologies, such as CRISPR/Cas gene-editing tool, is necessary to overcome the relapse and metastatic progression in TNBC patients.

### 3.6 Phototherapy

Phototherapy, such as photodynamic therapy (PDT) and photothermal therapy (PTT), is the modernization in the conventional tumor therapeutic techniques that attracted significant attention for TNBC clinical treatments and are being explored extensively in preclinical models that are discussed in the following sections.

#### 3.6.1 Photodynamic therapy (PDT)

PDT is an alternative therapeutic methodology that utilizes a photosensitizer (PS), a suitable excitation light source, and oxygen molecules for treatment [215]. It was begun in the mid-1900s, and Dougherty and his co-workers developed its modernized expression in1975. Much research has demonstrated that PDT has significant potential due to its non-invasive modality and site-specific-targeted treatment [8]. Basically, a specific wavelength is energized PS that shifts its vitality to the molecular oxygen, consequently producing responsive oxygen species (ROS), for example, hydrogen peroxide (H_2_O_2_), singlet oxygen (^1^O_2_), hydroxyl free radical (OH•) and superoxide ions (O_2_^¯^^•^) can oxidize macromolecules and prompting tumor cell removal. However, conventional PS has a limited therapeutic effect due to this reason the other excitation illumination sources have been explored, such as chemiluminescence (chemiluminescent emitter transfer light energy to the PS), Cerenkov radiation (charged particles produced electromagnetic radiation when passed through dielectric medium, resulting triggering local polarization), and X-rays (transposition of X-rays by energy transducer to optical fluoresces for PDT and radiotherapy) [215].

In recent years, PDT has been proven efficient in various types of breast-related carcinoma. For example, Ag decorated TiO_2_ nanorod (200 ​μg ​mL¯^1^) was used to generate excessive intracellular ROS under 30 ​min of 5.6 ​mW ​cm¯^2^ UV irradiation, resulting in activation of apoptosis and 90% breast cancer cell killing [216]. Further, PDT does not produce any ionizing radiations and systemic toxicity, which can alter the biological medium. Therefore, PDT has very special characteristics when contrasted with conservative diagnostic techniques because of its repeatability deprived of lesser injuriousness, reduced long-term morbidity, minimal invasiveness, and better life quality of the patients [215]. In spite of the extensively growing applications, PDT still has some complications in clinical adoption because of solid tumour hypoxia conditions. The greater consumption of oxygen molecules creates a hypoxia environment that further enhances tumour cell propagation, metastasis, and invasion, thereby rendering the impact of PDT [217]. Some other restrictions on the effectiveness of PDT include deficiency of an ideal PS, selecting a specific wavelength for eﬀective treatment, formulating PS, and monitoring the treatment response [218]. To enhance PDT effect, it is crucial to synthesize excellent PS, improve their solubility in aqueous mediums, and respond to the near-infrared region (NIR) range for deep tissue penetration [219]. Importantly, materials that can absorb light energy in the NIR region exhibited promising potential in PTT and related imagining [220,221].

#### 3.6.2 Photothermal therapy (PTT)

PTT is an emerging theranostics therapy that reveals advanced therapeutic precision, screens therapeutic impact, diminishes the harm to non-cancerous cells and standardises the tumor therapy in time. PTT mainly utilized photothermal agents, those are triggered through near-infrared lasers that can penetrate about 1–2 ​cm in tissues [222]. After penetration, it generates hyperthermia, which induces thermal ablation or immunogenic death of the cancer cells. However, PTT-based imagining involves laser-emitted light radiation absorbed by the living tissues and transformed into heat. Meanwhile, biological tissues can create supersonic rays by thermal vibration, and acoustic signals induced via illumination produce photoacoustic signal [8]. The photoacoustic signal created by tissues can change optical absorption to visual pictures.

PTT can reduce distant metastasis in breast tumour in a combinatorial trials such as radiotherapy and chemotherapy. In addition, the PTT agents are generally metal-based nanoparticles, carbon nanotubes, and graphene oxide. It stands out due to its minimal invasiveness, great specificity, and particular spatial-temporal perception. Moreover, diverse mechanisms of action are linked with PDT and PTT therapy, such effective treatments can be utilized as single or combinatorial trials to produce a synergistic impact against TNBC and therefore obtain better efficacy in irradiating tumour cells [215]. However, PTT has several disadvantages that preclude its clinical translation. For example, high-temperature PPT induces massive thermal diffusion by inhibiting heat-shock molecular chaperons/proteins, which cause more destruction in normal tissues compared to tumour mass, ultimately declining the PTT antitumour effectiveness. In contrast, low-temperature PPT (43–45 ​°C) suffers due to a decrease in tumour cell killing impact [223]. Other limitations include poor targeting capability of photothermal materials that can be improved by conjugating specialized targeting ligands or proteins with nanomaterials [224]. Nonetheless, more proteins or ligands-loaded nanomaterials may have a lesser dose of therapy drugs, which shows a weakened antitumour effect resulting in recurrence and metastasis or secondary melanoma development [223].

### 3.7 Sonodynamic therapy (SDT)

Sonodynamic therapy is chiefly based on the PDT and has revolutionized as advance cancer modality since it induces systemic immuno responses via ultrasonic radiation and chemical agents. Unlike PDT, SDT has deep penetration in the tumour tissues through sonosensitizers (ultra-sound stimulated sensitizer) and create ample cytotoxicity by triggering ROS to destroy breast tumour cells, therefore can be utilized as a cancer vaccine to treat TNBC [219]. Further, investigations have proved that SDT showed intense penetration in living cells compared to PDT. Mechanistically, non-thermal ultra-sound waves penetrate the targeted tissues, activating sonosensitizers that induce ROS-based cytotoxicity within the targeted sites without causing undesirable injury in the untargeted tissues. However, the most significant difficulty is the development of suitable parameters for SDT-based therapeutic efficacy. The unmet need for SD therapy is the development of steady and flexible sonosensitizers with excellent quality and safety. Moreover, to date, no effective criterion has been identified that provides ease in its application in clinical studies [218,219].

Additionally, recent studies have shown some effectiveness of SDT in tumour cell killing, such as a multifunctional system based on manganese-protoporphyrin (FA-MnPs) encapsulated in the folate-liposomes. The FA-MnPs nanosonosensitizer not only penetrated about 8 ​cm into the targeted tissues but also triggered immune responses in the TNBC mice model. Further, FA-MnPs nanosonosensitizer suppressed the growth of the superficial tumour, deep lesion, and re-polarized *M2* (immunosuppressive) macrophages into *M1* (antitumour) macrophages. Also, it induced T-lymphocytes, natural killer-cells, and dendritic cells to inhibit the growth of the tumour and initiate immunogenic cell death of TNBC cells [219]. Apart from inevitable success, SDT still exhibited limitations in terms of tumour shrinkage and tumor growth retardation that decrease its effectiveness in cancer studies. Additionally, current databases have revealed that no long-term clinical study will evaluate the impact of sonosensitizers and ultrasonic waves on normal human breast, TNBC, and prolonged exposure with SDT after-effects. Also, there is no standard setting for temperature management, sonosensitizers screening, tumour positioning, and ROS generation in TNBC and it is effective for some solid carcinomas [225]. Moreover, the common SDT sonosensitizers are organic lipophilic materials with poor circulation, short-lived, low loading capacity and accumulated asymmetrically in the tumour sites [218]. Thus, advance CRISPR/Cas-based technology is the most suitable and consistent solution for current issues.

### 3.8 CRISPR/Cas (CRISPR)-Based advances in TNBC gene therapy

Currently, genetic engineering-based technologies have been anticipated for the treatment or therapy of TNBC that is able to target any molecule in the pretentious part, as well as knockout and silent gene aberration/alternations [14,226]. However, DNA binding domain-dependent orthodox techniques, specifically zinc finger nuclease (ZFNs) and transcriptional activator-like effector nuclease (TALENs) gained exceptional importance in genetically engineered breast tissue organoids, cellular carcinoma models, and gene therapy investigations [14,227]. However, due to their intricacy and inefficient approaches, an extensive application of this therapy has been constrained [43]. On the other hand, RNAi has reflected a prodigious establishment for gene expression in tumour therapy. Despite its transitory silencing impact, it must be incessantly administrated to achieve a sufficient knockout level [31]. Presently, innovative archaea-derived adaptive immunity mechanisms earn significant amendments that are mechanistically based on double-stranded RNA endonuclease, called CRISPR-associated protein-9 (Cas9) [228]. This system has been explored to treat aggressive tumour pathology, and advanced gene therapy due to its easeful application, straightforwardness, time-saving, and high target efficacy with efficiency and accuracy [39,43,226].

#### 3.8.1 CRISPR-based genome editing and screening in TNBC

The genome-wide screening is a powerful method, proficient in spotting proteome or genes where spontaneous mutations drive tumour origination, and evolution is easefully tackled *via* CRISPR-Cas9 system that offers new modulates for targeting noncoding and coding genes [120]. It helps to investigate how overexpression or loss of function/complete gene silencing affects resistance/tolerance, immunosuppression, proliferation, invasion and metastasis in TNBC (Table 3). For instance, *TMEPAI* [102], *MCL-1*, EGFR [110], *BAG3* [37], *NSDHL* [100], *FOXC1* [4]*, CXCR4* and *CXCR7* [83] expression were seldom by CRISPR knockout gene-editing tool, while *PTEN* activation in TNBC *via* dead (d)-Cas9 merged with VP64-p65-Rta thereby repressing downstream AKT/mTOR/MAPK oncogenic signalling cascades [36]. Correspondingly, tRNA-based multiplex CRISPR/dCas9 aids in distinguishing diverse kinds of breast melanoma as HER2+, luminal A, luminal B and TNBC in OKMS1 cell line, where HER2+ proved as a possible lineage of luminal A, thereby shearing similar tumour stemness and treatment [229]. Additionally, the factual role of mTOR/Hippo pathway has been revealed by pharmacological inhibition of YAP oncoprotein and TORC1/2 in patient-derived xenografts *via* genome-wide CRISPR screening [59].

**Table 3:** A current research studies with CRISPR-based gene editing in TNBC population for chemotherapy and immunotherapy.

Tool	Targeted molecule	Target function	Study type	After CRISPR gene engineering
Gene manipulation				
**CRISPR/pSpCas9(BB)-2A-puro v2.0 plasmid**	*BAG3*	Negatively regulate ciliogenesis and induce therapy resistance	*In vitro*	*BAG3* silencing significantly induced cilia formation and disassembly, which promoted cell differentiation and decreased invasion, expression of *ZEB1* and *SNAI1* (induce EMT), *AURKA* (cilium disassemble) and *CDK1* (phosphorylate *PLK1* to disassemble cilia) and cell migration [37]
**CRISPR/Cas13a polylysine black phosphorus nanosheets NPs**	*MCL-1*	Enhance breast cancer cell survival,	*In vitro* & *in vivo*	Knockdown the expression of *MCL*-1 mRNA, decreased 58.64% *EGFP* expression and 55.96% *CXCR4* expression, which induced apoptosis (70.54%) and decreased cell proliferation (55.49%) of MDA-MB-231 *in vitro* and suppress 65.16% tumour growth *in vivo* [32]
**CRISPR/dCas9 plasmid and PEI-PBA-DMMA NPs**	miR-524	Suppress tumour by downregulating *SMAD2*, *HES1* and *TEAD1*	*In vitro* & *in vivo*	Activate and enhance 3.38 folds Pri-miR-524 expression level in MDA-MB-231, which retarded cell proliferation (64%) by reducing expression of oncoproteins (Smad2, Hes1 and Tead1), tumour development and induced apoptosis in tumour cells in subcutaneous xenograft model [230]
**LentiCRISPR/Cas9 -blast**	*NSDHL*	Promote metastasis via upregulating *TGF*-*β* and cholesterol biosynthesis pathways and suppressing TGFβR2 degradation	*In vitro* & *in vivo*	Inhibition in Nsdhl metabolism suppresses tumour, lung metastasis and invasion and enhances endosomal TGFβR2 protein degradation in MDA-MB-231LM2 and MDA-MB-231BO cells lines while overexpression of *NSDHL* increased migration and induction of *TGF*-*β* signalling in MDA-MB-231 ​cells. However, knockdown of *NSDHL* did not rescue metastasis and expression of TGFβR2 protein in SK-BR-3, BT474 and ZR-75-30 ​cells and nude mice [100]
**pLenti6.3 Cas9 vector**	*ITGA9*	Cell adhesion, lymphangiogenesis, and enhancement of β-catenin expression which promote tumour development and metastasis	*In vitro* & *in vivo*	Downregulation of *ITGA9* enhanced the reduction of β-catenin, which reduces cell proliferation of MDA-MD-231-LM2 and SUM-159, MDA-MB-453, BT-549 and BT474 ​cells. The knockout of *ITGA9* KO in orthotopic xenograft mouse model indicated a reduction in cancer stem cell-like characteristics, angiogenesis, tumour development and lung metastasis [103]
**CRISPR/pSpCas9 plasmid-GFP**	*CD81*	Receptor-dependent, intracellular and adhesion-mediated signalling, proliferate B-cells, enhance expression of membrane-bound CD19 in B-lymphocytes, promote EMT and regulate metastasis via activating TGF-β receptor	*In vitro*	Cell viability decreased by about 50% in MDA-MB-231 [231]
**LentiCRISPR/Cas9v2**	*CXCR4* & *CXCR7*	Interact with *CXCL12* and induce proliferation, metastasis, translocation, invasion in TNBC	*In vitro*	Single knockout of either *CXCR7* or *CXCR4* induced reduction in cell proliferation (26% in *CXCR7* & 32% in *CXCR4*), colony formation (53% in *CXCR7* & 56% in *CXCR4*), translocation (62% in *CXCR7* & 70% in *CXCR4*), invasion (60% in *CXCR7* & 68% in *CXCR4*) and prolonged the duration of G0/G1 (26% in *CXCR7* & 32% in *CXCR4*) thus provoke more number of cells in G2/M and S phase and delay G1/S phase alteration in MDA-MB-231 ​cell while co-blockage produced more reduction in tumour cell functions such as 50% inhibition in cell proliferation, 90% in migration, 72% in colony formation, 90% in invasion and 50% in G0/G1 cell cycle distribution [83]
**CRISPR/Cas9 plasmid PEG-modified cationic lipid NPs**	*PLK-1*	Tumour cell proliferation	*In vitro* & *in vivo*	Decrease cell proliferation in MCF-7 ​cells *in vitro* and 67% tumour growth *in vivo* [232]
**CRISPR/Cas9 plasmid anionic deformable nanolipogel**	*LNC2*	Induce cell proliferation and migration by facilitating EMT	*In vitro* & *in vivo*	Significantly reduce cell migration (60%), length (40%), height (10%) and mesenchymal transmutation, thereby inhibiting of filopodia development in MDA-MB-231 and MDA-MB-436 ​cells. *In vivo* blockage of *LCN2* inhibit 77% tumour growth and 69% tumour mass [233]
**LentiCRISPR plasmid**	*RNF208*	Suppress metastasis and cell proliferation in TNBC	*In vitro* & *in vivo*	Overexpression of *RNF208* protein enhanced degradation of vimentin protein, thereby suppressing lung metastasis and tumour development in MDA-MB-231 and Hs578T cells and NOD/SCID mice [234]
**CRISPR/Cas9 plasmid aptamer-functionalized PEG-PEI lipopolymer**	*VEGFA*	Induce lung metastasis, angiogenesis, facilities endothelial cell activation, form new vessels and prompt tumour cell in TME	*In vitro*	Significantly inhibit cell proliferation by downregulating *VEGFA*, metastasis by inhibiting Ezrin, invasion by reducing *MMP9* and enhancing apoptosis by blocking Survivin in MDA-MB-231 [235]
Epigenetics				
**CRISPR/Cas9 HDR plasmid**	*SNAIL*	Transcriptional regulator, enhance metastatic potential, control early embryogenesis, EMT phenotype, regulate more than 100 genes for polarization differentiation	*In vitro*	Deletion of *SNAIL* caused abnormal regulation of several hundred genes involved in chromatin organization, tumorigenesis and in epithelial transmutation in MDA-MB-231 and Hs578T cell and also declined Hs578T cell migration [236]
**pLenti6.3 dCas9- KRAB vector**	Screened 2500 superenhancer including *BAMBI*	Tumour progression, poor prognosis and metastasis	TNBC tumour sample	122 enhancers were conserved, 206 super-enhancers and 1646 enhancers were presented in normal samples, while 2643 super-enhancers and 23,946 enhancers were tumour-related and not found in a normal sample. Moreover, proximal enhancer BAMBI correlated with tumour-dependencies in TNBC [237]
**pLKO.1CRISPR/SpCas9 vector**	*ANLN, FOXC1* & *MET*	Dysregulate genes and induce tumorigenesis	*In vitro*	CRISPR screening identified 6284 no-union super-enhancers, 9996 union super-enhancers in which 7333 were TNBC-specific super-enhancers from BT549, HCC1937, MDA-MB-436, MDA-MB-231, SUM149, SUM159, and MDA-MB-468 TNBC cell lines. TNBC-specific super-enhancers targeted 9 transcriptional factors and 1785 genes, among them *FOXC1*, *MET*, *MYC* and *ANLN* regulated spheroids development, clonogenicity and invasion of TNBC tumour uncovering super-enhancer heterogeneity in TNBC and identified several oncogenes relationship between temporal regulatory retort and transcription factor binding [4]
**LentiCRISPR/Cas9v2**	*TROJAN*	Induce proliferation, invasion and poor prognosis in TNBC patients by mortifying ZMYND8 factor (metastasis suppressing protein)	*In vitro* & *in vivo*	Significant suppression of TROJAN increased apoptosis, reduced proliferation and metastasis in MDA-MB-231 and BT549 ​cells and in xenograft NOD/SCID mice TNBC model [162]
Drug discovery				
**LentiCRISPR/Cas9v2**	*BBD*	Drug resistance/acquired resistance against inhibitors	*In vitro* & *in vivo*	JQ1 treatment after *BBD* knocked-out, effectively blocked *BRD2*, *BRD4* expression in SUM159, SUM149, MDA-MB-231 and MDA-MB-436 ​cells. Drug synergy matrix and deletion of several genes such as *BRD9*, *BRD7*, *ARID1A MED1*, *MED19*, *MED24*, and *PBRM1* enhance JQ1 sensitivity while deletion of kinase singling genes like *TGFβR1*, *TEAD1*, *PIK3CA*, *TGFβR2* enhance JQ1 treatment retort. Deletion of *PLK1* and *AURKA* kinase inhibitor (BUB3), and Vincristine and Paclitaxel demonstrated strong synergy with JQ1 *in vitro* and *in vivo*. However, the toxicity of BCL-2, BCL-XL inhibitors and JQ1 limit the therapies in clinical trials [94]
**pLentiCRISPR/dCas9-SAM plasmid**	*OCT4*, *KLF*, *MYC & SOX2*	Induce pluripotent stem cells, and overexpression of these genes increase proliferation, invasion and tumour stemness and alters *NF-ĸβ* and *MAPK* signalling pathways	*In vitro*	After engineering of 4 genes in the MCF-7 ​cell, it showed similar susceptibility against Tamoxifen as SK-BR-3 (HER2+) cells shown after 72 ​h, conversely cell proliferation and migration patterns were similar in engineered-MCF-7 ​cells and MDA-MB-231, indicated that TNBC share identical tumour stemness with HER2 breast tumour but require different therapeutic approaches for effective prevention [229]
**LentiCRISPR/Cas9v2**	*mTOR1*/2 & *YAP*	Promote tumorigenesis in drug resistance via activating several tumour inducing genes	*In vitro* & *in vivo*	Improved Verteporfin and Torin1 retort and reduced tumour progression, induced apoptosis (73.96%) and decreased tumour cell survival in SUM159PT, SUM149PT, SUM1315MO2 and MDA-MB-231 and shrink tumour mass and blocked tumour development in patient-derived xenograft NSG mice, proved fruitfulness of gene engineering in combinatorial drug selection and development [59]
**LentiCRISPR/Cas9v2**	Loss of function screening of 2240 genes	Enhance genetic vulnerabilities in TNBC	*In vitro*	Loss of function screening sensitized Everolimus (mTOR inhibitor) and Fluorouracil (chemo-drug) retort. Downregulation of *FASN* and *EGFR* further enhanced drugs retort in mesenchymal and epithelial phenotypes, respectively in D492 and D492 ​M cells [29]
Chemotherapy				
**CRISPR/Cas9KO and HDR plasmids**	*DSTYK*	Chemoresistance against doxorubicin plus docetaxel in TNBC	*In vitro* & *in vivo*	Apoptosis of chemoresistance cells after silencing and dual drug (Docetaxel and Doxorubicin) induction in SUM102PT and MDA-MB-468 ​cells and NOD/SCID mice [238]
**CRISPR/Cas9 plasmid**	*LIFR*	block EC359 and HDACi expression	*In vitro* & *in vivo*	Knockdown of *LIFR* and its inhibitor (EC359) treatment enhanced the potential of *HDAC* inhibitor that induced apoptosis and reduced cell survival and growth of TNBC cell-derived xenografts, patient-derived explants and xenografts *in vivo* [239]
**All-in-one Cas9-T2A-EGFP plasmid**	*PARP1*	Single-stranded DNA break/repair	*In vitro*	Disruption in exon 7 of *PARP1* increased MDA-MB-231 and MDA-MB-436 response for Docetaxel, Doxorubicin and Gemcitabine treatment in 2D-model while, silencing of *PARP1* did not produce fruitful impact in 3D tumour-on-a-chip model, suggesting the dose-dependent retort under TME [240]
**CRISPR/pCas9_GFP plasmid**	*TMEPAI*	Negatively regulate *TGF*-*β* and *Smad* signalling thereby converting *TGF*-*β* into tumorigenic form and inducing epithelial to mesenchymal transmutation	*In vitro*	Increase BT549 and MDA-MB-231 TNBC cell response for chemotherapy drugs such as Doxorubicin and Paclitaxel but lesser retort for Cisplatin and Bicalutamide [102,241]
**LentiCas9 mCherry and sgRNA-GFP**	*MCL-1* & *BAK*	Regulate mitochondrial respiration, cell survival in the normal and neoplastic cell, and induce cell proliferation and metastasis as a result of poor patient survival and chemotherapy resistance	*In vitro* & *in vivo*	Upregulation of *MCL*-*1* and downregulation of *BAK*, induced resistance in SK-BR-3 ​cells against S63845 treatment *(MCL-1* inhibitor) for cell death. BAK upregulation caused resistance against apoptosis while single knockout of *BAK* has not reversed resistance to *MCL-1* inhibitor. Further, Taxane Docetaxel treatment with inhibitor, reduced tumour growth and increased OS in MDA-MB-231 and MDA-MB-468 and patient-derived xenograft model *in vivo* [242]
**SpCas9-RNP electroporation**	*MALAT1*	Angiogenesis, phosphorylation, promote proliferation, invasion, and chemo-drug resistance	*In vitro*	Deletion of *MALAT1* promoter region enhanced sensitivity in BT549 and MDA-MB-231 for Doxorubicin and Paclitaxel neoadjuvant chemotherapy [164]
Immunotherapy				
**CRISPR/Cas12a albumin-bound mesoporous liposomes**	*RICTOR*	Activate or alter *M1* macrophages into *M2* macrophages phenotype that induce tumorigenesis under the influence of TME	*In vitro* & *in vivo*	decrease immunosuppression, block *M2* differentiation, increase 85% expression of *M1* anti-cancer phenotype of macrophages and post-Paclitaxel chemotherapy effect, also elevate the expression about 100 folds of pro-inflammatory genes such as *MCP-1, MIP-1α, MIP-1β, RANTES* and *IL*-*1β* and reduce the expression of anti-inflammatory genes like *IL-10, IL-4* and *M-CSF* genes that induce *M2* macrophages differentiation [243]
**LentiCas9 mCherry, LentiCRISPR/Cas9 v2 puro & blast**	*Cop1*	Mediate chemokine stimulation and macrophages infiltration, enhance immune checkpoint blockage retort by targeting *Trib2*-dependend C/ebpδ protein degradation	*In vitro* & *in vivo*	Deletion of *Cop1* augment anti-tumour immunity, decline secretion of macrophages-related chemokines and macrophages infiltration in TNBC mice model, and enhance C/ebpδ protein, which suppresses genes of chemoattractant for macrophages stabilization thereby improving immunotherapy efficacy in TNBC under TME [244]
**CRISPR/SpCas9 plasmid aPBAE cationic copolymer**	*Cdk5*	Regulate or enhance expression of PD-1/PD-L1 pathway, participate in apoptosis, angiogenesis and senescence	*In vitro* & *in vivo*	70% knockout of *Cdk5* showed strong T cell-mediated immuno retort in TME that automatically downregulate the expression of *PD-L1* with *IFN-γ* by about 25% and 15% without *IFN-γ* in 4T1 cells, which increased CD8^+^ T cells (49.65%) and decreased T regulatory (Tregs) cells, inhibition of CDK5 decrease the expression of p53 (37.8%) and PD-L1 (27.7%) proteins and reduce 89.62% tumour growth and 75.7% tumour weight in 4T1 mice model [26]
**LentiCRISPR/Cas9 -Blast**	*LGALS2*	Induce tumour macrophages, polarization and proliferation of *M2* macrophages by CSF1/CSF1R receptor axis, thereby promoting immunosuppression and TME	*In vitro* & *in vivo*	Blockage promote tumour suppression, reverse immunosuppression, enhance antitumour immunity, decrease tumour cell proliferation (40%) in 4T1 cells while its knock-in indicated that Lgals2 accumulate around CD11b^+^ cells and effect TME by downregulating CD45^+^CD3^+^ cells (T cells), CD45^+^CD19^+^ (B cells), CD45^+^CD3ˉCD49b^+^ (NK cells), CTLs (cytotoxic T lymphocytes) and CD45^+^CD3^+^CD8^+^ cells. Also, there is a lower expression level of IFN-γ and granzyme B but a greater level of *PD-1*, *TIM-3* and CD45^+^CD11b^+^*in vivo*. However, anti-LGALS2 antibody treatment showed partial reversion of immunosuppression in the TME [245]
**LentiCRISPR/Cas9v2**	*STING*	Produce proinflammatory cytokines	*In vitro* & *in vivo*	Blockage decrease *PARP* and proinflammatory signalling that eliminates Olaparib-prompted CD8^+^ T cell infiltration, which changed TME of *BRCA-1* and *TP53* deficient MDA-MB-436 ​cells and mice model. Also, it enhanced 241 days of survival in mice via activating cGAS/STING signalling pathway [246]
**LentiCRISPR/Cas9v2**	*UBR5 & PD-L1*	Immunosuppression, activate E3 ligase, provokes protein-protein interaction, regulates ubiquitin, cell cycle, translational control and DNA damage retort	*In vitro* & *in vivo*	*UBR5* silencing and *IFN-γ* induction in MDA-MB-231 and BT549 ​cells caused 32–54% *PD-L1* mRNA stimulation and 49–66% proteins stimulation, decreasing *IRF1* and *STAT1* levels in 4T1 cells. Selective downregulation of *UBR5* reduced tumour growth within 30 days in 4T1 mice, while dual knockout (*PD-L1* & *UBR5*) showed more reduction in tumour growth and recurrences until 122 days in mice [247]

*ANLN*, anillin actin binding protein gene; aPBAE, AMP-modified poly(β-amino ester) polymer; *ARID1A*, AT-rich interaction domain 1A; *AURKA*, aurora kinase A; *BAG3*, BAG cochaperone 3 or BCL2-associated athanogene 3; *BAK*, Bcl-2-antagonist/killer 1; BAMBI, BMP and activin membrane bound inhibitor; *BBD,* BET Bromo- and extra-terminal domain; *BCL-2*, B-cell lymphoma 2; *BCL-XL*, BCL-2 and BCL-2L1; *BRCA* 1/2, breast cancer gene 1/2; *BRD2*, bromodomain containing 2; *BRD4*, bromodomain containing 4; *BRD7*, bromodomain containing 7; *BRD9*, bromodomain containing 9; BUB3, budding uninhibited by benzimidazoles 3; *CD81*, cluster of differentiation 81; *CDK1*, cyclin-dependent kinase 1; *CDK5*, Cyclin-dependent kinase 5; *cGAS*, cyclic GMP-AMP synthase; *Cop1*, constitutive photomorphogenic 1; CSF1, colony stimulating factor 1; CSF1R, colony stimulating factor 1 receptor; *CXCL12*, C-X-C motif chemokine ligand 12 or stromal cell-derived factor or chemokine ligand 12 or interleukin 12 (IL-12); *CXCR4*, cytokine-cytokine receptor 4; *CXCR7*, cytokine-cytokine receptor 7; *DSTYK*, dual serine/threonine and tyrosine protein kinase; EC359, Leukaemia inhibitory factor receptor inhibitor; *EGFR*, epidermal growth factor receptor; EMT, epithelial to mesenchymal transmutation; *FASN*, fatty acid synthase; *FOXC1*, Forkhead box C1; H3K27 Ac, acetylation of histone H3 at lysine 27; HDAC, histone deacetylase; HDACi, histone deacetylase inhibitors; *HES1*, Hes family BHLH transcription factor 1; *IFN-γ*, interferon gamma; *IL-10*, interleukin 10; *IL*-*1β*, interleukin 1 beta; *IL-4*, interleukin 4; *ITGA9*, integrin alpha 9; *KLF*, kruppel like factor; *LCN2*, lipocalin 2; *LGALS2*, lectin galactoside-binding soluble 2; *LIFR α*, Leukaemia inhibitory factor receptor; *LIFR*, Leukaemia inhibitory factor receptor; *MALAT1*, metastasis associated lung adenocarcinoma transcript 1; MAPK, mitogen-activated protein kinase; *MCL-1*, myeloid cell leukaemia 1; *MCP-1*, monocyte chemoattractant protein 1; *M-CSF*, macrophage colony stimulating factor; *MED1*, mediator complex subunit 1; *MED19*, mediator complex subunit 19; *MED24*, mediator complex subunit 24; *MET*, receptor tyrosine kinase proto-oncogene; *MIP-1α*, macrophage inflammatory protein-1 alpha; *MIP-1β*, macrophage inflammatory protein-1 beta; *MMP9*, matrix metallopeptidase 9; *mTOR*, mammalian target of rapamycin; *MYC*, myelocytomatosis protein; *NF-ĸβ*, nuclear factor kappa-light-chain-enhancer of activated B *NSDHL*, NAD (P) H steroid dehydrogenase-like protein gene; *OCT4*, organic cation/carnitine transporter 4; *PARP*, poly (ADP-ribose) polymerase; *PARP1*, poly (ADP-ribose) polymerase 1; *PBRM1*, polybromo 1; *PD*-*1*, programmed cell death-1; *PD-L1*, programmed cell death ligand-1; PEI-PBA-DMMA, cationic core made by dCas9 plasmid and polyethyleneimine-phenylboronic acid and shell is synthesized 2,3-dimethylmaleic anhydride-modified poly(ethylene glycol)-b-polylysine; *PIK3CA*, phosphatidylinositol-4,5-bisphosphate 3-kinase catalytic subunit alpha; *PLK-1*, polo-like kinase 1; RANTES, is a chemokine (C–C motif) ligand 5 encoded by CCL5 gene in human; *RICTOR*, rapamycin-insensitive companion of mTOR; *RNF208*, RING finger 208 protein; SAM, synergistic activation mediator plasmid; *SMAD2*, phosphor-SMAD family member 2; *SNAI1*, Snail transcriptional repressor 1; *STING*, Stimulator of interferon genes; *TEAD1*, transcription enhancer domain 1 or TEA domain transcription factor 1; *TGF*-*β*, transforming growth factor-β; *TGFβR1*, transforming growth factor-β receptor 1; *TGFβR2*, transforming growth factor-β receptor 2; *TIM-3*, T cell immunoglobulin and mucin domain-containing protein 3; TME, tumour microenvironment; *TMEPAI*, transmembrane prostate androgen-induced protein gene; *TP53*, tumour protein 53; *Trib2*, tribbles pseudokinase 2; TROJAN, the AK124454 sequence named and function as lncRNA; *UBR5*, ubiquitin protein ligase E3 component-N-recognin 5; *VEGFA*, vascular endothelial growth factor A; YAP, Yes association protein; *ZEB1*, Zinc finger E-box binding homeobox 1; ZMYND8, zinc finger MYND-type 8.

Some other regulatory elements are successfully knocked out by utilizing CRISPR gene technology. For example, BBDIs (Bromodomain inhibitors) targeted inhibition of *BET* as a candidate therapeutic aid for TNBC relapse, while intrinsic and acquired tolerance against BBDIs frontier clinical potential. However, silencing with CRISPR and JQ1 small-molecule inhibitors revealed *BET* signalling pathway of activation, *SRC*, *AXL*, *YAP* signalling, systemic regulation and chemo-resistance in TNBC cell lines SUM149, and SUM159 [94]. Similarly, Snail1 is a zing finger protein, abundantly synthesized and enhanced EMT transition *via* interconnecting 185 genes through *cis*-regulatory channel and *CXCR-LASP1* axis in the SUM-159, T47D, MCF7, BT549, MDA-MB-436, MDA-MB-231, MDA-MB-468, and BT20 ​cells model. The CRISPR-based dual 10 base pair excision in the first and second exon of transcriptional factor *Snail1*, uncovered its partial involvement in EMT phenotype in TNBC [248]. Further, CRISPR immensely exploited in the loss-of-function screen of 2240 genes [29], 2500 novel super-enhancer, including *TGF-β* pseudo receptor dependencies on BAMBI [237], and 104,592 ​cell's ATAC profiling [249] to detect specific phenotypic susceptibilities in TNBC.

#### 3.8.2 CRISPR/Cas aid in transcriptomic analysis

Previously utilized lncRNA, miRNA, shRNA, and siRNA-dependent interference or silencing is imperfect, and the residual mRNAs may still perform a functional part, thereby preventing identification of the target site that entails mRNA complete deactivation [120,166,250]. This issue can be solved *via* CRISPR/Cas9 or CRISPR/dCas9 [120]. For instance, synergistic drug matrix, drug target efficacy, and mischaracterization can now be better analysed *via* CRISPR/siRNA-based silencing, which explains that OTS964, a small antagonist is the potential inhibitor of *CDK11* in TNBC, and multiple carcinoma addiction over *CDK11* [251]. Similarly, CRISPR/Cas9 directed deletion of the distal promoter region of lncRNA *C1orf132* or *MIR29B2CHG*, residing between *CD34* and *CD46* protein-coding region, repressed the expression of the miR-29b2 and miR-29c, enhanced chemoresistance and triggered the worst prognosis in TNBC cells. However, upregulation of *MIR29B2CHG via* CRISPR/Cas9 suppresses progression, EMT, cell migration, and mammary gland growth pathways in TNBC [128] that were substantiated as an idealistic tool for TNBC transcriptome editing, silencing and understanding the complex mechanism of disease regulation. For example, a wider transcriptomic knockout screening via CRISPR/Cas 9 of human endogenous retroviruses indicated that the overexpression of lncRNA TORJAN played an important role in tumour propagation, invasion and poor prognosis in TNBC patients (FUSCC cohort 1–2). Further antisense targeting therapy of TORJAN has significantly decreased the tumour cell proliferation *in* xenograft *in vivo* model [162]. This study proves the effectiveness of CRISPR-based gene editing in TNBC model.

#### 3.8.3 CRISPR aids in TNBC biomarker and drug discovery

The CRISPR-dependent genome-wide array screening is a potent strategy to detect and classify biomarkers for early TNBC prognosis and diagnosis. For instance, an estrogen-inducible E3 ligase called RING finger protein 208 (*RNF208*), overexpression is engineered by CRISPR/Cas9, induced proteasomal degradation of Vimentin protein resulting suppression in TNBC cell proliferation. CRISPR/Cas9-based activation/overexpression of RNF208, interact with a serine residue (Ser39) of phosphorylated Vimentin protein and polyubiquitinated its lysine residue (Lys97) of the head domain of Vimentin protein leading to proteasomal degradation, which facilitates suppression of lung metastasis and invasion in TNBC [234]. The negative feedback of RNF208 protein or inhibitors serves as a prognostic biomarker in TNBC drug development. However, drug development for TNBC is a multistep and challenging process in which a series of genetic hurdles such as drug target structure correlation, tissue exposure and disease selectivity, drug target conformation and authentication, clinical dose, drug efficacy [252], prolong testing of about 12 years and huge expense that may exceed $ 1 billion are involved. Therefore, CRISPR/Cas system proved to be suitable for large-scale screening medicine targets. It mainly helped to generate multiple insertion/mutations, knockout, knock-in screening, and activate and suppress desired targets. For example, CRISPR/Cas-based genome-wide screening in patient-derived xenograft model established new combinatorial Verteporfin-Torin1 TNBC therapy. The pharmacological blockage of YAP/TEAD binding by Verteporfin (YAP inhibitor), and mTORC1/2 inhibition by Torin1 (ATP-competitive specialized inhibitor) indicated poor TNBC cell proliferation. Mechanically, 50 ​nM of Torin1 downregulates mTORC1/2, which provokes macropinocytosis and Verteporfin uptake, while 0.6 ​μM Verteporfin mediates YAP reduction leads to programmed cell death or apoptosis in TNBC cells. This study revealed the robustness of CRISPR/Cas system where CRISPR blocked dual druggable targets, which produced a better antitumour effect in the TNBC *in vivo* model. Also, it can be effectively compared with monotherapy approaches for TNBC [59].

#### 3.8.4 CRISPR/Cas-based therapeutic in chemotherapy

Apart from drug development, CRISPR augments the effectiveness of chemotherapy as TNBC heterogeneity, including breast cancer stem cells evolution and tumour microenvironments (TME), which are among the protruding aspects accountable for the catastrophe of chemotherapy and chemo-drug resistance [42,253]. For example, MALAT1 lncRNA knockdown enhances Paclitaxel and Doxorubicin sensitivity in TNBC *via* altering several lncRNA (LINC-PINT, NEAT1, and USP3-AS1), and suppressing *STAT1*, *NUPR1*, *SREBF1*, *RELA*, interferon regulatory factor 1 (*IRF1*), *ERAP1*, aminopeptidase regulator, histone-associated proteins such as H3C12, H1-5, and H2AC4, insulin-like growth factor-binding protein 1 (*IGFBP1*), granulin precursor (GRN), angiogenin, and oxidative phosphorylation reputable pathway [164]. Similarly, *PARP1*-generated deficiency in MDA-MB-436 and MDA-MB-231 ​cell lines prominently decrease the Doxorubicin, Gemcitabine and Docetaxel dose necessities to realize therapeutic effectiveness in even 3D tumour-on-a-chip model [240]. Thus, the intensely expressed transmembrane prostate androgen-induced protein (*TMEPAI*), induced by TGF-β/Smad signalling, propel *TGF-β* to perform a tumorigenic role in TNBC patients [254]. More importantly, complete silencing of exon 4 of *TMEPAI* by sgRNA declined resistance against Paclitaxel, Doxorubicin, but negligible tolerance for Bicalutamide and Cisplatin, indicating partial potency of *TMEPAI* knockout for chemotherapeutic usage [102]. However, BCL-2 family antagonist BH3 mimetics like S63845 is now widely applied as a potent tool in chemo-immuno combo-therapy [242]. Moreover, after repeated cycles of chemotherapy, drug resistance or chemoresistance is a common response generated by tumour cells, which is the most critical problem. Therefore, most mono and combo-therapies have failed to cure TNBC. However, CRISR/Cas9 gene therapy has offered an advanced therapeutic approach to overwhelm chemoresistance in tumour cells. For example, complete *BAG3* silencing *via* CRISPR/Cas9 downregulated the expression of *ZEB1* and *SNAI1* that induce EMT in TNBC, reduce TNBC cell migration and penetration in brain tissues as well as overwhelmed therapy resistance [37]. In another study, CRISPR/Cas9-dependent knockdown of serine/threonine and tyrosine protein kinase (*DSTYK*) induces cell death of chemoresistance cells upon 10 ​μM Doxorubicin plus 100 ​nM Docetaxel combinatorial application *in vitro* and orthotopic mouse model [238]. These findings provide the central role of CRISPR gene technology in developing advanced and quick methods to tackle complex chemoresistance mechanisms in TNBC chemotherapy (Table 3).

#### 3.8.5 CRISPR/Cas-based therapeutic in immunotherapy

The complex and more immuno-based heterogeneous nature of TNBC makes its profiling complicated due to the involvement of various signalling molecules, intra and extra cellular matric, specialized T-cells along with diverse kind of mutations, and genomic instability. In this context, several exogenous inductions of *PD-L1* by PI3K/AKT/mTOR, JAK/STAT1/IRF1, NF-κB/JAK/STAT3, ECM, *IFN-γ*, EGFR, interleukins (ILs), *TNF-α, NPM1* or B23, vascular endothelial growth factor receptor-2 (*VEGFR2*), and *PARP1* were used to enhance responses during TNBC relapse [104]. However, the meticulous transcriptional control of *PD-L1* in TNBC remains contentious [192]. Furthermore, several ongoing clinical or pre-clinical trials with combinatorial, targeted, ligand-specific, NAC or chemo-immunotherapies are being assessed to classify foreseeable biomarkers that will advance treatment consequences among TNBC interventions. Though the prototype is ever-changing, much consideration is being subjected to improving CRISPR-based treatments for TNBC.

The maximum TNBC patients do not retort to anti-PD-1/PD-L1 immunotherapy [13], which may be due to the immuno exclusion process, and several agents and immune checkpoint markers or inhibitors have been developed to address this issue [13,255]. For instance, CD47 protein jam-packed blocking elevates immune ripostes [38]. Though, immune devastation is one of the imperative factors in TNBC tumorigenesis progression [256], carcinoma cells deter immune effector cells through excretion of extrinsic aspects by disturbing the (TME) [207]. Recently, an innovative effort has been made to understand the immuno exclusion induced by ECM. In this study, the discoidin domain receptor 1 (*Ddr1*) gene was entirely silenced by All-in-One Lentivector CRISPR/Cas9 system in E0771, M-Wnt and AT-3 mouse mammary tissues, which revealed the potent involvement of untethered-extra cellular domain (ECD) of DDR1 protein. The discoidin domain receptors (DDRs) belong to the non-integrin collagen receptors family that have tyrosine kinase activity, are expressed limitedly in adulthood, and are widely studied in tumorigenesis. The DDR1-ECD interacts with collagen, disturbs fibre alignment, induces immuno exclusion, deter immunity, and uses collagen fibre in tumour defence. Additionally, its membrane-bound intracellular kinase domain assists DDR1-ECD against antitumour T-cells [255]. This CRISPR-based finding gives new insight into why anti-PD-1/PD-L1 immunotherapy is only effective with fewer TNBC patients. Therefore, a new antibody against DDR1 needs to be developed to reduce relapse and metastasis and enhance the overall life span of the TNBC population.

Further, the carcinoma-precise polyclonal memory CD4^+^, and CD8^+^T cells are supposed to be a prime target during immune checkpoint inhibitors documentation [204]. However, the CRISPR/Cas9-interceded gene manipulation has endeavoured to systemic discourse challenges apropos immune mechanism flopping from innumerable prospects [257]. The T cell-dependent immunotherapy is credited to the application of *ex vivo* exploited T lymphocytes directed to abolish tumours with tumour-associated antigen (TAA) or CAR-T cells, and T cell receptors (TCR)-engineered T lymphocytes as its foremost approaches [207,258]. Interestingly, accumulation of TCRs is directly proportional with better tumour rheostat in patients, as it declines with the aging including former immunological exposures like chronic infection [204]. For CAR-T cell therapy, the T cells possibly originated from an allogeneic (cells derived from another person) or autologous (cells taken from the same person) donor. In contrast, autologous T cells utilization is hypothetically inefficient process and as well as mostly depends on the quantity and quality of autologous T cells harvested from the subjected patient. On the other hand, allogeneic grafted T cells challenged substantial barriers due to the TCR on donor's T lymphocytes, and endogenous MHC class I complex that posse alloreactivity and graft-versus-host disease (GVHD) [207]. Therefore, CRISPR/Cas9 gene-editing technology inserts the CAR gene and confiscates the TCR effectively [257]. For example, EGFR is one of the highly expressed receptors on TNBC cells, whose expression successfully engineered via third-generation CAR-T targeting EGFR in TNBC both *in vivo* xenograft mouse model and *in vitro*, revealed limited cytotoxicity, retard tumour cell growth, enhanced *IFN-γ*, activate *PARP*, and Fas-associated death domain (FADD), and caspase signalling [259]. Likewise, CRISPR-engineered CAR-T cells with scFv of grafted monoclonal TAB004 antibody coupled to CD3 and CD28 for mutant glycosylated tumour of *MUC1* (MUC28z), recognized 95% in TNBC cases, significantly victimized cytotoxicity over an array of human TNBC cells, upon identification of mutant *MUC1*, *MUC28z* CAR-T cells elevate synthesis of *IFN-γ*, granzyme B, Th1 and other cyto- and chemokines [24]. Therefore, a single dose of CRISPR/Cas9 engineered CAR-T cells significantly reduces tumour growth in NOD-Prkdc^scid^ IL2rg^null^ (NSG) mice xenograft model [35].

#### 3.8.6 CRISPR aids in clinical studies

CRISPR yield significant importance in pre-clinical tumour science research, and because of this, it transformed into clinical studies. To date, almost about 18 clinical trials have been registered in the National Library of Medicine (https://www.clinicaltrials.gov), including non-small cell lung cancer (NCT02793856), EBV positive advanced stage malignancies (NCT03044743), human papillomavirus-related malignant neoplasm (NCT03057912), oesophageal (NCT03081715), prostate (NCT03525652), solid tumour (NCT03747965; NCT03545815), renal cell (NCT04438083), gastro-intestinal (NCT04426669), CD_19_+leukaemia and lymphoma (NCT03166878), hepatocellular carcinoma (NCT04417764), central nervous system tumour (NCT03332030), multiple myeloma (NCT04244656; NCT03399448), relapsed or refractory leukaemia and lymphoma (NCT03398967), relapsed or refractory B-cell melanoma (NCT04035434), T-cell malignancies (NCT03690011), and B acute lymphoblastic leukaemia (NCT04557436). Most of the CRISPR clinical trial is based on PD-1 dependent immunotherapy, while other were conducted to assess the efficacy and safety issues regarding CRISPR [41]. Besides, CRISPR-engineered autologous CAR-T cell's clinical studies are limited to lung cancer or non-small-cell lung cancer [260,261]. Previously, cells were collected, engineered, propagated, and then induced, resulting in low efficacy and frequent death of the individual due to prolonged treatment delay. However, advanced CRISPR-based allogeneic T-cell therapy is more powerful, cost-effective, and rapid than autologous CAR-T cell therapy. Hitherto, the first allogenic CRISPR-engineered CD_19_ edited T-cells (CTX_110_) were injected into the 143 ​B-cell malignant patients followed by fludarabine and cyclophosphamide standard dose, which initially showed nephrotoxicity in one patient (NCT04035434). In addition, autologous CRISPR-dependent T-cell clinical trials showed fewer off-target and on-target impacts, such as alteration in the non-coding region of genes (1.69%) [262]. Importantly, off-target-induced undesirable mutations are deceased after some time in the patients. Moreover, patients with the higher expression levels of engineered T cell exhibit fewer after-effects like fatigue, rash and fever [262]. This concluded that it might be safe and effective to administer CRISPR-engineered CAR-T cells to the TNBC patients to overcome poor prognosis and increase DFS and OS.

## 4 CRISPR/cas components

The CRISPR-Cas gene-editing technology is principally based on the immuno-adaptive mechanism of prokaryotes against an invasion of foreign DNA/RNA [43]. Until now, CRISPR-Cas gene editing system is broadly divided into two major classes and further sub-divided into six types that again branch into 33 sub-types. The CRISPR/Cas system (Class-I and Class-II) is mainly based on site-specific Cas endonuclease and CRISPR-RNA [39]. The Class-1 consists of multiple effector protein complexes, while Class-2 CRISPR-Cas systems have a single effector protein complex. Further, Class-1 consists of Type I (CRISPR-Cas3), III (CRISPR-Cas10), and IV (CRISPR-Cas6), while Class-2 comprises of Type II (CRISPR-Cas9), V (CRISPR-Cas12 and CRISPR-Cas14), and VI (CRISPR-Cas13) as given in Table 4 [25,43,[263], [264], [265]].

**Table 4:** Classification of CRISPR/Cas with respect to interference effectors components.

Class	Type	Sub-Type	Features	Effector	Nuclease Domain	Target
Class-I	Type-I	A, B, C, D, E, F & G	Multi-subunit effector CASCADE	Cas3	HD	dsDNA [34,43]
	Type-III	A ​= ​Csm	Multi-subunit Csm & Cmr_5_ effector complex	Csm3 Cmr4	Autocatalytic	ssRNA [34,43]
		B=Cmr				
		C				
			Multi-subunit Csm & Csx1 effector complex	Csm6 Csx1	HEPN	ssRNA [34]
		D	Multi-subunit Csm2-Csm5 effector complex	Cas10	HD	ssRNA [34,266]
	Type-IV	A, B, C, D, E, A1, A2 & A3	Multi-subunit effector complex crRNA-like sgRNA	Cas8/Csf1	RecD helicase/HD	ssRNA [34,43,267]
Class-II	Type-II	A, B & C	Single multi-domain effector, single sg RNA	Cas9	RuvC & HNH	dsDNA [26,57,235,268]
	Type-V	A ​= ​Cpf1/Cas12a	Single effector, single crRNA	Cas12	RuvC-like x2	dsDNA [27,53,269]
		B=C2c1/Cas12b				
		C=C2c3/Cas12c				
		D ​= ​Cas Y/Cas12d				
		E ​= ​Cas X/Cas12e				
		A ​= ​Cas14a (6v)	Single effector, single sgRNA	Cas14	RuvC	ssDNA [270]
		B=Cas14b (16v)				
		C=Cas14c (2v)				
	Type-VI	A ​= ​Cas13a/C2c2	Single effector, single crRNA	Cas13	HEPN x 2	ssRNA [32,271]
		B=Cas13b				
		C=Cas13c				
		D ​= ​Cas13d				

V, variants; ds, double-stranded; ss, single-stranded

### 4.1 Mechanism of Class-I CRISPRs

Class-I, Type-I complex is identified in *Escherichia coli*, consists of eleven sub-components of five Cas endonucleases such as Cse-1_1_, Cse-2_2_, Cas5_1_, Cas6e_1_ and Cas7_6_ that have 405 ​kDa weight and called CRISPR-associated complex for antiviral defense (CASCADE) where Cas3 and its effectors cleaved CRISPR RNA precursor and retain these products. These cleaved RNAs serve as single small guide RNA, which sufficiently interfere with foreign DNA. Further, Class-I, Type-III CRISPRs widely occur in archaea species such as Cas10 that combine themselves in a cascade-like multi effector complex and then identify and subsequently kill the invaded foreigner RNA [266]. More detail of Class-I CRISPR's action mechanism has been studied elsewhere [43].

### 4.2 Mechanism of Class-II CRISPRs

The most commonly used and explored system in biomedical engineering is Class-II due to its simplicity and ease of targeting efficiency. This system is based on a single effector to detect, bind and target the desired sequence, discussed concisely in sub-sections.

#### 4.2.1 CRISPR/Cas9

**Fig. 4:**
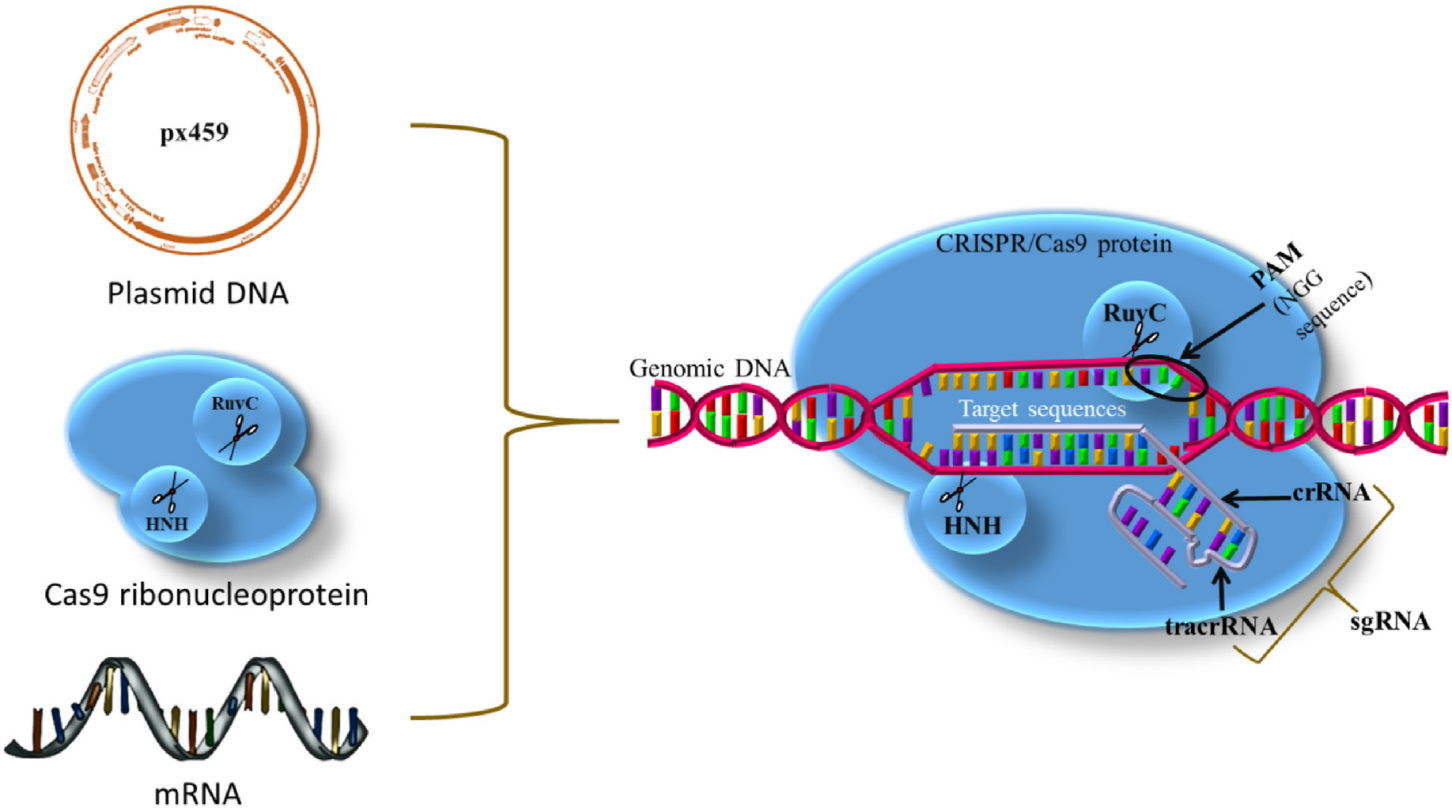
Representative triple-negative breast cancer targeted gene engineering via various systems of clustered regularly interspaced short palindromic repeat (CRISPR). Plasmid, Cas9 ribonucleoprotein, and mRNA-based gene engineering have edited the genome adjacent to the protospacer motif (PAM). The Cas9 ribonucleoprotein or RNP possessed two nuclease motif, RuvC, and HNH that generate a blunt-end double-strand break at the desired site for gene engineering.

CRISPR-Cas9 system generally consists of Cas9 DNA endonuclease enzyme, and single guided-RNA (sgRNA) [264]. The Cas9 protein is sectioned into two lobes, the alpha lobe, and endonuclease activity lobe. This endonuclease lobe is further divided into two domains, named the HNH domain and RuvC domain. The HNH domain cuts the specific targeted sequences of the DNA strand, while RuvC cleaves the non-targeted DNA strand, but it is deactivated due to D10A mutation (Fig. 4) [43]. The sgRNA has great importance in the CRISPR-Cas9-based targeting system efficacy, specificity, and precision [240]. This sgRNA is composed of two diverse RNAs one RNA work as a guider named guide RNA (gRNA) or CRISPR RNA (crRNA), and the other woke for targeting named as *trans*-activating CRISPR RNA (tracrRNA). The crRNA in the sgRNA can recognize a PAM (proto-spacer adjacent motif) sequence (5’-NGG) that usually consists of about 3–5 nucleotides and then match the remaining 20 nucleotide sequences with the targeted host genome [39]. Further, the starting 10–12 nucleotides sequence at 3’ end of the sg RNA that is near to the PAM (proto-spacer adjacent motif) knows as seed sequence that binds at the targeted site in the host for genome editing purpose. Upon complete recognization, tracrRNA pair with crRNA and recruit Cas9 protein to create double-stranded breaks (DSBs) at any PAM-comprising targeted site within the identify sequences of the targeted host genome. Created DSBs can be either repair by homology-directed repair (HDR) or non-homologous end-joining mechanism (NHEJ). Importantly, NHEJ produce frameshift mutation *via* spontaneous deletion or insertion, thereby promoting permanent gene silencing as compared to HDR.

#### 4.2.2 CRISPR/Cas12

CRISPR/Cas12 (Cpf1) is another robust system that falls in Type-V of Class-II and was discovered in *Francisella* and *Prevotella* in 2015 (Table 4). Unlike CRISPR/Cas9 sgRNA, this system contains only crRNA and Cas12 endonuclease, which generates sticky ends, cleaves hairpin structure of pre-crRNA and produces transitional crRNA that further undergoes processing to become mature crRNAs. Cas12 has more advantages than Cas9 protein due to the small size Cas12 (1.3 ​kb), guide RNA (42–44 nucleotides) and thymine rich PAM sequences [272] that can address several limitations of CRISPR/Cas9 delivery issues [27,272]. It is a RNA-directed DNA nuclease that offers immunity in bacterium and significantly integrates into the mammalian cell editing system [269,272].

#### 4.2.3 CRISPR/Cas14

CRISPR/Cas14 is solitarily detected in uncultivated symbiotic archaea species and another robust and the simplest CRISPR in CRISPR/Cas family (Table 4). Cas14 gene has 24 variants grouped in Cas14a, Cas14b and Cas14c. It codes exceptionally small size (400–700 amino acids) RNA-guided endonucleases with a molecular weight about 40–70 ​kDa, half of all existing Cas proteins. It has a single RuvC catalytic domain, suggesting that it may act as stand-alone effector. It binds and cleaves single-stranded DNA as Cas13. Nonetheless, Cas14-directed cleavage is more specialized than other CRISPR/Cas systems. Moreover, Cas14 does not require PAM sequences to recognize targeted DNA [270], and it can be effectively exploited in TNBC pathogenesis and TME manipulation.

#### 4.2.4 CRISPR/Cas13

The newly discovered Type-VI CRISPR/Cas13 endonuclease can only cleave single-stranded RNA with the help of 28 nucleotides long crRNA at the protospacer flanking site (PFS) [39,273]. Further, this system is similar to Type-V for crRNA processing and maturation but has unique higher eukaryotes and prokaryotes nucleotide-binding (HEPN) domains that are exclusively related to RNase not detected in the rest of the CRISPR/Cas system. After specific site recognization and binding, Cas13 activates cleavage activity to the untarget RNAs. Nonetheless, Cas13 collateral cleavage action has not been fully established except it cleaves target sequence in multiple uracil residue sites [273]. Hitherto, this tool has been utilized in some biomedical engineering applications but has much more potential to detect tumour circulating RNA in cancer patients, viral-based pathogenesis [273] in tumour, and manipulation of tumour RNA such as microRNA, lncRNA and mRNA.

Several endonucleases have been designed to enhance efficacy and decrease off-target impacts, such as Cas9 nickase, dead Cas9 (dCas9) and dead Cas12a (dCas12a), whose nickase domain is synthetically point-mutated. Despite prompt breakage, they activate or silence gene expression and maintain the integrity of binding of CRISPR/Cas complex [32,273].

## 5 Engineering system for CRISPR-based targeting in TNBC

Recently, CRISPR/Cas system has been synthesized by plasmid, ribonucleoprotein (RNP), and mRNA-based methods to screen, treat, and engineer *in vitro* and *in vivo* TNBC genome at their desired location, as shown in Fig. 4.

### 5.1 Plasmid-based genome editing

The plasmid-based CRISPR/Cas9 system, including pX333 [4], pX335 or pX330, pX459 [37,143,153,234], pcDNA-3.1 [13,89,274], pLKO-based plasmid [162,237], and dual vector bearing-plasmid system [83,110] is a modest and lucrative alternative approach for RNP, which signifies tractability in scheme owing to the affluence of DNA sequences integration into plasmids retaining unpretentious molecular cloning systems [94]. Nevertheless, the efficacy of gene engineering is frequently limited by gene expression proficiency and nuclear distribution, which is required to improve the critical gene therapy-dependent multiplexes. This coordination demonstrates advanced steadiness in contrast sgRNA/Cas9 mRNA complex [31]. Plasmid high replication capacity inside the cell makes nuclear ingress easeful *via* eruption of the nuclear membrane. Nonetheless, the transportation in post-mitotic cells to the nucleus typically befalls through nuclear pores that can be meek by the binding sequences of the transcription factor in the plasmid that intermingle with cellular importins [14]. Generally, in systems cases, comprising a CRISPR tracrRNA, sgRNA-guided *Streptococcus pyogenes* Cas9 (spCas9), U6promoter, and restriction sites are packed in the same plasmid [275]. Even though this system has great potential, its larger plasmid DNA (pDNA) genome size hinders efficient supply, thereby decreasing biodistribution, efficacy, and excessive on-set deferment in genetic manipulation of targeted gene/genes [34]. Additionally, the high off-target impact is the extra crucial disadvantage of the plasmid-based CRISPR delivery (Table 5) [264].

**Table 5:** CRISPR/Cas system for triple-negative breast cancer.

Type	Size	Charge	Advantages	Limitations
Cas/sgRNA Plasmid	4.2–10.0 ​kb	Negative	Good stability and enzymatically tolerant, low-cost, and both sgRNA plus Cas mRNA can express together in the nucleus	Integration risk or high off-target impact, low efficacy, and biodistribution [232,233,276]
Cas sgRNA and Ribonucleoprotein (RNP)	160 ​kDa	Positive	Low off-target impact, rapid genome engineering, and low systemic toxicities	High-cost, no mass production, and low delivery efficacy [30,46,164]
Cas sg RNA and mRNA	4500 ​nt	Negative	Low off-target impacts, transient expression, no risk of integration in the host genome and both sgRNA plus Cas mRNA can be co-delivered and translated in to the cytoplasm	Poor stability, autoimmune issues [277,278]

### 5.2 Cas9-RNA ribonucleoprotein-based genome editing

The Cas9-RNP delivery system into the targeted tumour cells is the hotspot and pragmatic approach [268]. The RNP complex has enough simplified genome engineering stratagem attributable to the fact that translation/transcription consequently promoter assortment, and codon improvement are not prerequisites [43]. This may conceivably increase the proficiency of genome-engineering in hard-to-transfect cells or post-mitotic, in which limitations to translational/transcriptional capability of the cell can lead to worse genome-engineering efficiency when compared to plasmid mRNA/DNA [34]. Together, this scheme also declines the cell type quality since transcriptional directing cannot be engaged [14]. It also signifies a fleeting practical genome-engineering method that can cause lesser off-target impact, immuno stimulation, and cytotoxicity (Table 5) [264]. Nevertheless, some staid challenges inclusively reduce delivery efficacy due to the enlarged size of Cas9 endonucleases protein, which still relics to be cracked [47]. Additional inadequacy of the RNP complex methodology is a considerable endotoxin impurity, which can be created together by bacterial activity and high cost with no mass production [268].

### 5.3 mRNA-based genome editing

The mRNA-based scheme is projected to decrease genome excision deferral wherein mRNA indoctrination sgRNA and Cas9 have been delivered to target tumour cells [43]. Besides, Cas9 endonuclease transitory manifestation in this system might be a two-edged sword, but it can then donate for plummeting the off-target impact, while excessively less expression time resulting in reduced competence [34,277]. The mRNA instability remains one of the main problems for this delivery approach (Table 5) [47]. However, this method has been frequently utilized in embryo engineering and zygote genome editing.

## 6 Delivery vehicles for CRISPR/cas-based targeting in TNBC

**Fig. 5:**
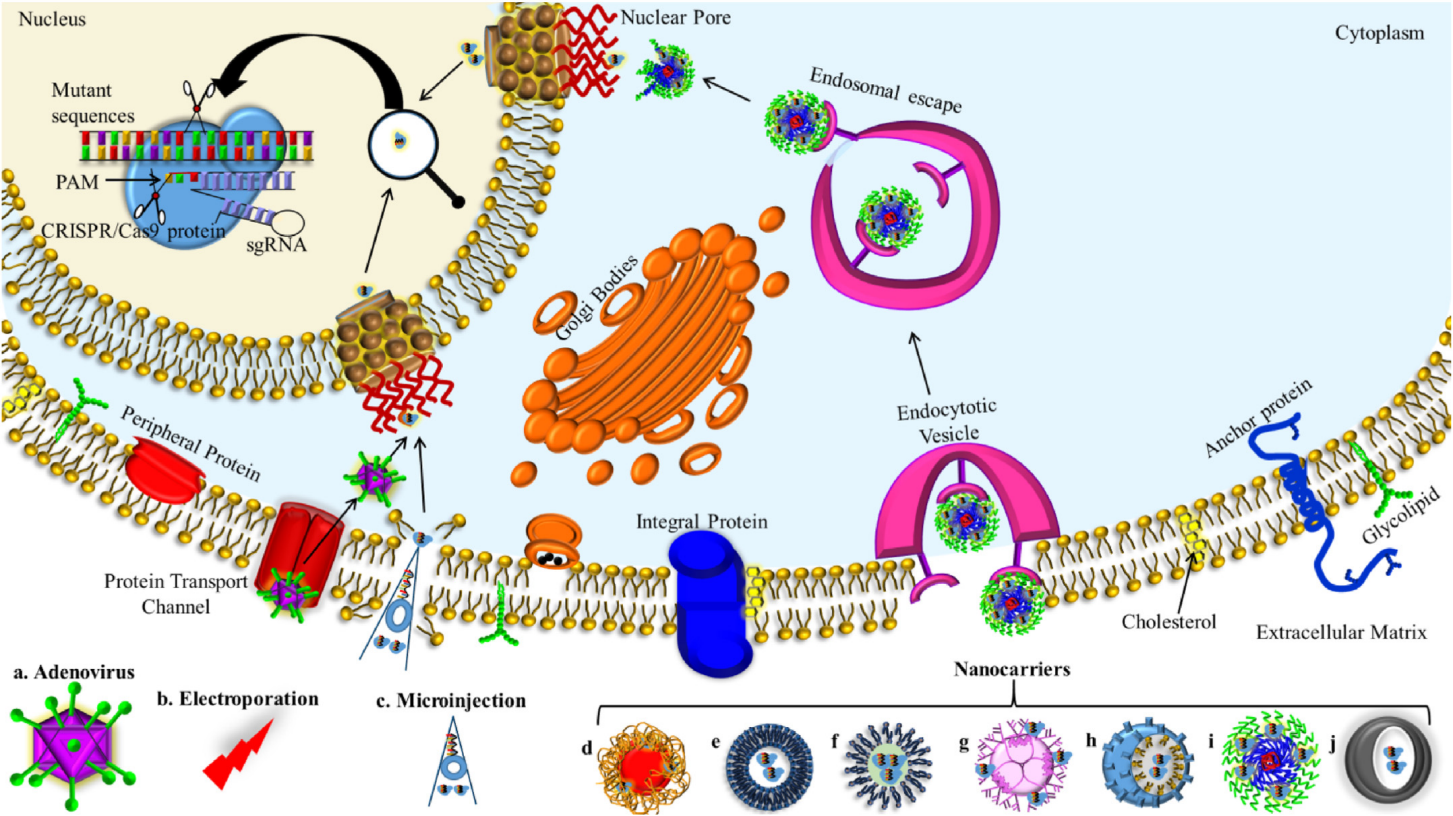
Indication of delivery vehicles for CRISPR/Cas9-based genome editing. (a) Demonstrate the virus-based transfection that delivers CRISPR/Cas9 cargo into the tumor cell's nucleus. (b) Electroporation induces physical stress on the plasma membrane of tumor cell resulting in payload transportation inside the cell. (c) Microinjection causes mechanical destruction within the tumor cell's phospholipids bilayer and injected the CRISPR/Cas9 and sgRNA complex for mutant gene editing. The most advanced CRISPR/Cas9 delivery vehicles are shown from d to j where (d) albumin nanocarrier, (e) nanoliposomes, (f) nanolipogel, (g) dendrimer, (h) polymersomes, (i) polymer micelle, (j) iron-based nanocarrier deliver efficiently into the nucleus of tumor cells *via* endolysosomal escape.

The CRISPR-mediated genome editing can be achieved *via* three distinct methods such as (i) physical method for transporting CRISPR/Cas9 system, (ii) lentiviral/adenoviral-based delivery system, and (iii) nanocarriers-based delivery, which is summarised in Table 6 and demonstrated in Fig. 5.

**Table 6:** CRISPR/Cas gene manipulation in triple-negative breast cancer and its sgRNA sequences.

Gene	Perform editing	sgRNA sequence (5'˗3')	Vector/System
*AURKB*	Knockout	GCTCCTTGTAGAGCTCCCCG	LentiCas9 [95]
*ZAK*	Knockout	CCTTGGTTGGAACTTTCCCA	LentiCas9 [95]
*FOXC1*	Knockout	GTCCATCTCTGGTATATCTC	Plasmid px333 [4]
*MET*	Knockout	ATGTGGCTGTCAGCATAAGT	Plasmid px333 [4]
SE *ANLN*	Knockout	GTGTTGACAGTGGATGACTG	Plasmid px333 [4]
*TNC*	Knockout	CCCGGAGCTCATACTGCCCT	LentiCRISPR (Pxpr_001) [274]
*BAMBI*	Knockout screening	(TSS-1) GCGTCCCTAGAGTCGAGCG;	pLKO plasmid [237]
		(TSS-2) AGCAACTTGTCGCGACCTG;	
		(enh_1) CCTATATGTGAATCCACCT;	
		(enh_2) GTAATCCCAACTACTCCGG;	
		(enh_3) AGTCAGTATACCAACACTG;	
		(enh_4) GAACCTGGACATCCTCCAC;	
		(enh_5) AGACCGGGTTTCAGCACGT;	
		(enh_6) ATGTAACACATACCCACTG;	
		(enh_7) CCCCACGTAGCATCACCCA;	
		(enh_8) GTCTAATGTGTGATAACTG	
*WDR59*	Knockout	ATATCCGCACATCGCCGTCA	LentiCRISPR v2 [59]
*RICTOR*	Knockout	CCATCTGAATAACTTTACTA	LentiCRISPR v2 [59]
*SAVI*	Knockout	GGAGGTGGTTGATCATACCG	LentiCRISPR v2 [59]
*FRMD6*	Knockout	CTTCCGTGTGCAGTACTATG	LentiCRISPR v2 [59]
*WDR59*	Activate	AGGCGCGGTGTAGCAATTGG	LentiCRISPR v2 [59]
*RICTOR*	Activate	CATTTGGACGACGGCTTCCG	LentiCRISPR v2 [59]
*SAVI*	Activate	(gRNA-1) CCTGCCGACTGAGAAGATGA;	LentiCRISPR v2 [59]
		(gRNA-2) CTTCTCGCTGAGGATGAGTG;	
		(gRNA-3) GACTCGGGTGCCGGCGCTCT;	
*FRMD6*	Activate	(gRNA-1) GGAGCTGCGCGCTGAGCTCG	LentiCRISPR v2 [59]
		(gRNA-2) GGAGGGGTGCGGCCACTTGG	
		(gRNA-3) GGACCCAACCAAGCGTCCCG	
*SESN3*	Activate	(gRNA-1) ACAACAACCCTGGTTTCCTT;	LentiCRISPR v2 [59]
		(gRNA-2) TTCGTAGAATGAAATCTATG	
*TMEPAI*	Knockout	TTTCTTGGTTTATATATCTTGTGGAAAGGACGAAACACCG	Plasmid #41824 [241]
*PARP1*	Knockout	(gRNA-1) GGTCCAAGATCTGCAGCCAGTGG	Cas9-T2A-EGFP [240]
		(gRNA-2) GGCAGAGCCTGTTGAAGTTGTGG	
		(gRNA-3) GGTAAGCACAGGGCTACCAGGGG	
*BAX*	Knockout	(gRNA-1) GTGACCTATGAACTCAGGAGTC	Lenti-Retroviral [242]
		CTTTAGTGTGCGGTGGATGC	
		(gRNA-2) GTGACCTATGAACTCAGGAGTC	
		GGCACTGGTTCTCCTCTCTC	
*BAK*	Knockout	(gRNA-1) GTGACCTATGAACTCAGGAGTC	Lenti-Retroviral [242]
		CTATGGGATGCTCTGCCCAC	
		(gRNA-2) GTGACCTATGAACTCAGGAGTC	
		CTATGGGATGCTCTGCCCAC	
*BIM*	Knockout	(gRNA-1) GTGACCTATGAACTCAGGAGTC	Lenti-Retroviral [242]
		TGTTTTGTTCTGATGCAGCTTCC	
		(gRNA-2) GTGACCTATGAACTCAGGAGTC	
		GACCAAATGGCAAAGCAACC	
*BID*	Knockout	(gRNA-1) GTGACCTATGAACTCAGGAGTC	Lenti-Retroviral [242]
		CAAGAAGGTGGCCAGTCACA	
		(gRNA-2) GTGACCTATGAACTCAGGAGTC GAGTCTGCTCTGTCTCTGCC	
*NOXA*	Knockout	(gRNA-1) GTGACCTATGAACTCAGGAGTC	Lenti-Retroviral [242]
		GGGCGTATTAGGTTTTGCTGG	
		(gRNA-2) GTGACCTATGAACTCAGGAGTC	
		GGGCGTATTAGGTTTTGCTGG	
*BAD*	Knockout	(gRNA-1) GTGACCTATGAACTCAGGAGTC	Lenti-Retroviral [242]
		GAGTCGCCACAGCTCCTAC	
		(gRNA-2) GTGACCTATGAACTCAGGAGTC	
		CCTACCCACTGACCCTCTGC	
*BMF*	Knockout	(gRNA-1) GTGACCTATGAACTCAGGAGTC	Lenti-Retroviral [242]
		TACCCAGACTCTCAGCCCAG	
		(gRNA-2) GTGACCTATGAACTCAGGAGTC	
		GAGGTTGGAGCAGTTGTGGA	
*BOK*	Knockout	(gRNA-1) GTGACCTATGAACTCAGGAGTC	Lenti-Retroviral [242]
		CCGAGATCATGGACGCCTT	
		(gRNA-2) GTGACCTATGAACTCAGGAGTC	
		CAGCCCCTTCCTTAAGTGCT	
*BIK*	Knockout	(gRNA-1) GTGACCTATGAACTCAGGAGTC	Lenti-Retroviral [242]
		(gRNA-2) TGCTCCTGCAGTAATGGCTT	
		(gRNA-3) GTGACCTATGAACTCAGGAGTC	
		(gRNA-4) TGCTCCTGCAGTAATGGCTT	
*HRK*	Knockout	(gRNA-1) GTGACCTATGAACTCAGGAGTC	Lenti-Retroviral [242]
		GAGGCCAGCGGTCATGTG	
		(gRNA-2) GTGACCTATGAACTCAGGAGTC	
		ACAAGGAGAAACTTGGTGTCCA	
*OCT4*	Knockout	(gRNA-1) GGAAAACCGGGAGACACAAC	pLenti-U6-sgRNA-PGK-Neo [229]
		(gRNA-2) GGATGTTTGCCTAATGGTGG	
*KLF4*	Knockout	(gRNA-1) CTCTTTCCGCCTGTTCCCGG	pLenti-U6-sgRNA-PGK-Neo [229]
		(gRNA-2) CAGTTCACGCTGCACAGTGC	
*MYC*	Knockout	(gRNA-1) AGCTAGAGTGCTCGGCTGCC	pLenti-U6-sgRNA-PGK-Neo [229]
		(gRNA-2) GAACCCGGGAGGGGCGCTTA	
*SOX2*	Knockout	(gRNA-1) AAACAGCACTAAGACTACGT	pLenti-U6-sgRNA-PGK-Neo [229]
		(gRNA-2) GCCCCCTTTCATGCAAAACC	
*ATG9A*	Knockout	TGCCCTTCCGTATTGCACG	pSpCas9 [279]
*CXCR4*	Knockout	GTTTCAGCACATCATGGTTG	Double Lentiviral vector LV-GFP [83]
*CXCR7*	Knockout	CATGATTGCCAACTCCGTGG	Double Lentiviral vector LV-GFP [83]
Spear-ATAC	Knockout	GCCACTTTTTCAAGTTGATAACGGACTAGCC TTATTTAAACTTGCTATGCTGTTTCCAGCTT	pSP618 [249]
		AGCTCTTAAAC	
*ITGA9*	Knockout	(gRNA-1) GGTGCTGGCGCTGGTGGTCGCGG	pX-459 [103]
		(gRNA-2) CCTCGACCCGCAGCGCCCCGTGC	
*EWSR1*	Knockout	GGTTGCACAGTAAGTGGCGGGG	pLVSpCas9 [275]
*FLI1*	Knockout	CCCTTGTCGCAGTGTGGCCCACTC	pLVSpCas9 [275]
*RNF208*	Knockout	(gRNA-1) CCTGCTTCCCGCCTGCTCCCCGG	pX-459 [234]
		(gRNA-2) TTGTGAATCAGTACGTGATTCGG	
		(gRNA-3) CGGGGGCTTGACCAGGACCCAGG	
*CDK11B*	G579S Knockout	(gRNA-1) CTTCCCGATCACGTCGCTGA	LentiV_Cas9_puro [251]
		(gRNA-2) TGATCGATTTCTGACTTCCC	
		(gRNA-3) GGTGACTTCGGGCTGGCGC	
		(gRNA-4) CGTTGCAGGTGGGTGACTTC	
		(gRNA-5) TCGTTGCAGGTGGGTGACTT	
*TP53*	Knockout	AGATGGCCATGGCGCGGACG	LentiV_Cas9_puro [251]
*AAVS1*	Knockout	(gRNA-1) ACTGTTGACGGCGGCGATGT	LentiV_Cas9_puro [251]
		(gRNA-2) GCTGATACCGTCGGCGTTGG	
*AURKA*	Knockout	(gRNA-1) CGACCTTCAATCATTTCAGG	LentiV_Cas9_puro [251]
		(gRNA-2) GGTAGACTCTGGTAGCATCA	
*BRAF*	Knockout	(gRNA-1) TTGAAGGCTTGTAACTGCTG	LentiV_Cas9_puro [251]
		(gRNA-2) ATGGAGATGGTGATACAAGC	
*CASP3*	Knockout	(gRNA-1) TACCCGGGTTAACCGAAAGG	LentiV_Cas9_puro [251]
		(gRNA-2) GAAGCGAATCAATGGACTC	
*MAPK14*	Knockout	(gRNA-1) CGATCCTGATGATGAACCAG	LentiV_Cas9_puro [251]
		(gRNA-2) CACAAAAACGGGGTTACGTG	
		(gRNA-3) TGGACGTTTTTACACCTGCA	
		(gRNA-4) AGACAGGTTCTGGTAACGCT	
		(gRNA-5) TGATGAAATGACAGGCTACG	
*MEK1*	Knockout	(gRNA-1) TATGGTGCGTTCTACAGCG	LentiV_Cas9_puro [251]
		(gRNA-2) AACATCCTAGTCAACTCCCG	
*PAK4*	Knockout	GCGATGCACACGATGCCCG	LentiV_Cas9_puro [251]
*PBK*	Knockout	AAGACACAGACTGCCATCAT	LentiV_Cas9_puro [251]
*PCNA*	Knockout	(gRNA-1) CTACCGCTGCGACCGCAACC	LentiV_Cas9_puro [251]
		(gRNA-2) GAGTATAAAATTGCGGATAT	
*PIM*	Knockout	CTGGAGTCGCAGTACCAGGT	LentiV_Cas9_puro [251]
*Rosa26*	Knockout	(gRNA-1) ACAGCAAGTTGTCTAACCCG	LentiV_Cas9_puro [251]
		(gRNA-2) CCGAAAGATTGGACACCCC	
*RPA3*	Knockout	(gRNA-1) CCCAGGTCGCGCATCAACGC	LentiV_Cas9_puro [251]
		(gRNA-2) GGTTGGAAGAGTAACCGCCA	
*BAG3*	Knockout screening	(1f) AAACCACTGTTTATCTGGCTGAGTC	pSpCas9(BB)-2A-puro (pX459) [37]
		(1r) CACCGACTCAGCCAGATAAACAGTG	
		(2f) AAACCAGAGGTCCCAGTCACCTCTC	
		(2r) CACCGAGAGGTGACTGGGACCTCTG	
		(9f) AAACCAGTTCGGAATCGCTGCATC	
		(9r) CACCGATGCAGCGATTCCGAACTG	
*TROJAN*	Knockout	(gRNA-1) CTACCTCATTCAGACACCAT	LentiCRISPR v2 [162]
		(gRNA-2) GTCAGACTGTAAGAGTGTCC	
*FUT8*	Knockout	(gRNA-1) ACCGGGGATGAAGACTGTCTACAA	LentiCRISPR v2 [13]
		(gRNA-2) ACCGACAGCCAAGGGTAAATATGG	
		(gRNA-3) ACCGTGAAGCAGTAGACCACATGA	
		(gRNA-4) CACCGAATTGGCGCTATGCTACTGG	
		(gRNA- 5) CACCGCTTACCTGACCAGTGTCCAG	
*ADSL*	Knockout	(gRNA-1) TGTGCTTCGTGTTTAGCGAC	LentiCRISPR v2 and pLKO plasmid [89]
		(gRNA-2) ACAGGTATAAATTCCGGACA	
*Ctrl*	Knockout	GCGAGGTATTCGGCTCCGCG	LentiCRISPR v2 and pLKO plasmid [89]
*EglN2*	Knockout	(gRNA-1) AGAGGTGGCTGTGGCTCTGG	LentiCRISPR v2 and pLKO plasmid [89]
		(gRNA-2) GCAGCGCCTTCACTCTGCAG	
*RB1*	Knockout	(gRNA-1) TCCTGAGGAGGACCCAGAGC	LentiCRISPR v2 and pCMV-VSV-G [93]
		(gRNA-2) CGGTGGCGGCCGTTTTTCGG	
		(gRNA-3) GGACAGGGTTGTGTCGAAAT	
*ITGA5*	Knockout	(gRNA-1) GGGGCCCCGAGAGTACTGCTGGG	pX459 [143]
		(gRNA-2) GGGGCAACAGTTCGAGCCCATGG	
*CD47*	Knockout	CACCGTAAATATAGATCCGGTGGTA	LentiCRISPR v2 [38]
*Cdk5*	Knockout	(gRNA-1) CCGGGAAACTCATGAGATTG	pX330/PBAE copolymer [26]
		(gRNA-2) CAGGCTGGATGATGACGATG	
		(gRNA-3) GGTGTGCCAAGTTCAGCCCTC	
		(gRNA-4) CAACGTGCTACATAGGGACC	
*FPN*	Knockout	GGGAGATCGGATGTGGCACTTTGCGGTG	Fe3O2 magnetic nanoparticle [280]
*LCN2*	Knockout	AGTTCACGCTGGGCAACATTAAGAGTTA	Fe3O2 magnetic nanoparticle [280]
*PLK-1*	Knockout	(sgPLK-1a) TCACCGAAGCTCTAGAGCCTG	Cas9/sg*PLK-1* plasmid/PLNP nanoparticle [232]
		(sgPLK-1b) ACTTCGTGTTCGTGGTGT	
		(sgPLK-1c) CTATGATGGATGCCGTTT	
*LCN2*	Knockout	(gRNA Plasmid-1) AACGAGTTACCTCGTCCGAG	Plasmid/tNLG nanoparticle [233]
		(gRNA Plasmid-2) AACGAGTTACCTCGTCCGAG	
		(gRNA Plasmid-3) CGGCCCTCACCTAAACAGGA	
*LRRC31*	Activate	(gRNA-1) GACTTCGAGACTTTGCAGCAT	Plasmid/ABTT nanoparticle [209]
		(gRNA-2) GTGCTCCCTCACGTCAGAAGA	
		(gRNA-3) GCAGTGCTTGAACATCGTCAG	

*AAVS1*, adeno-associated virus strain 1; ABTT, autocatalytic brain tumour-targeted nanoparticle (60% polymer-based nanoparticle); *ADSL*, adenylosuccinate lyase; *ATG9A*, autophagy-related protein 9A; *AURKA*, aurora kinase A; *AURKB*, aurora kinase B; *BAD*, BCL2-associated agonist of cell death; *BAG3*, BAG cochaperone 3 or BCL2-associated athanogene 3; *BAK*, BCL2 antagonist/killer; *BAMBI*, BMP and activin membrane-bound inhibitor; *BAX*, BCL2-associated X protein; *BID*, BH3 interacting domain death; *BIK*, BCL2 interacting killer; *BIM*, BCL2-like 11; *BMF*, BCL2 modifying factor; *BOK*, BCL2-related ovarian killer; *BRAF*, B-raf kinase; BSA, bovine serum albumin; *CASP3*, caspase 3; *CD47*, cluster of differentiation 47; *CDK11B*, cyclin-dependent kinase 11B; *Cdk5*, cyclin-dependent kinase 5; *Ctrl*, chymotrypsin like; *CXCR4*, cytokine-cytokine receptor 4; *CXCR7*, cytokine-cytokine receptor 7; *EglN2*, prolyl hydroxylase EGLN2; *EWSR1*, ewing's sarcoma breakpoint region 1; *FLI1*, friend leukaemia virus integration site 1; *FOXC1*, forkhead box C1; *FPN*, ferroportin gene; *FRMD6*, FERM domain containing 6; *FUT8*, α-1,6-fucosyltransferase; *HRK*, hara-kiri bcl2 interacting protein; *ITGA5*, integrin alpha 5; *ITGA9*, integrin alpha 9; *KLF4*, kruppel like factor 4; *LCN2*, lipocalin 2; *LRRC31*, leucine-rich-repeat-containing protein 31; *MAPK14*, mitogen-activated protein kinase 14; *MEK1*, MAPK/extracellular-signal-regulated kinase 1; *MET*, receptor tyrosine kinase proto-oncogene, receptor tyrosine kinase gene; *MYC*, myelocytomatosis protein; *NOXA*, NADPH oxidase activator 1; *OCT4*, organic cation/carnitine transporter 4; *PAK4*, p21-activating kinase 4; *PARP1*, poly (ADP-ribose) polymerase 1; PBAE, poly (β-amino ester) copolymer; *PBK*, PDZ binding kinase; *PCNA*, proliferating cell nuclear antigen; *PIM*, proto-oncogene serine/threonine kinase; PLNP, polyethylene glycol phospholipid-modified cationic lipid nanoparticles; *PLK*-*1*, polo-like kinase 1; *RB1*, retinoblastoma tumour suppressor 1; *RICTOR*, rapamycin-insensitive companion of mTOR; *RNF208*, ring finger protein 208; *RPA3*, replication protein A3; *SAVI*, STING-associated vasculopathy of infantile-onset; *SE ANLN*, super-enhancer of anillin actin binding protein; *SESN3*, sestrin 3; *SOX2*, SRY-Box transcription factor 2; Spear-ATAC, technique based on ATAC sequences to evaluate the impact of perturbing regulatory factors expression with respect to genes; *TMEPAI*, transmembrane prostate androgen-induced protein; *TNC*, Tenascin-C; tNLG tumour-targeted nanolipogel system; *TP53*, tumour suppressor protein 53; TROJAN, the AK124454 sequence named and function as lncRNA; *WDR59*, WD repeat domain 59; *ZAK*, leucine-zipper and sterile-α-motif kinase.

### 6.1 Virus-based delivery of CRISPR/Cas9

Lentivirus, adenovirus (Ad), adeno-associated virus (AAV) and retrovirus-based CRISPR delivery are commonly used strategies to tackle redox, pH, enzymes, and other multiple stimuli-responsive elements/environments [25,46,281]. For example, the lentivirus-CRISPR complex can screen clonogenic growth, impairment of tumour spheroid, and related oncogenic characteristic of TNBC cells [162]. Additionally, an array of culprit TNBC genes/transcriptional factors such as *FOXC1* [4], *EgLN2* [89], *FOXO3a* [133], miRNAs [143], the critical function of RING finger protein 208 (RNF208) [234], and super-enhancers [237] was significantly knocked in and knocked-out. Regardless of several recompenses of viral systems as excellent proficiency in genome engineering, some essential restrictions are also present, such as prospective insertional mutagenesis for innumerable viral vectors like lentiCRISPR v2 [38,59], constraint in desired sequence enclosement, time-consuming and challenging large-scale method/synthesis, and immune response stimulation that edge therapeutic solicitations [282].

### 6.2 Physical methods for delivering CRISPR/Cas9

The physical control for spatiotemporal expression of the CRISPR/Cas9 complex has become popular recently [283]. Its high precision and non-invasive strategies like optical, temperature, magnetic field, pH, ultrasound-responsive elements, including electroporation, and microinjection to deliver CRISPR along with space dimension and time-dependent release [25] are also noticeable. Thus, the high transfection competence can be offered through the physical method, but many serious apprehensions have been articulated together with inaptness for *in vivo* drives, but rather a poor cell viability and specificity [284]. Conversely, the hydrodynamic mode of delivering CRISPR/Cas can be offered as *in vivo* experiments [263]. However, distressing physiognomies may cause numerous physical or biological complications, for instance, dysfunction of the heart, high blood pressure, and liver enlargement (hepatomegaly). During the last couple of years, a great revolution has originated in the form of nanoparticle-based CRISPR/Cas9 delivery system [285]. Several of the previously-mentioned obstacles have not been experiential in this technique. Notably, a nonviral-based delivery vehicle proposes many benefits over virus-based delivery, for example, high DNA-wrapping competence, lessen immune-generated stimulation, easeful fabrication, and flexible strategy that are being specialized and targeted the sites in the living tissues or cells with negligible cytotoxicity for healthy cells [192,286]. The strategic inadequacy of these systems is the poor delivery efficacy regarding genes of interest compared with viral delivery systems [285]. Nevertheless, the gene conveyance efficiency and the expression profile of the *trans*-allele can be augmented ominously through the combination of diverse self-assemble innovative nanocarriers. In the ongoing era, advent of inimitable multifunctional carrier vehicles affords a vigorous auspicious delivery stratagem for TNBC therapy [14].

### 6.3 Nanocarriers-based delivery for improving CRISPR/Cas targeting in TNBC

The effectual and nontoxic transfer of dynamic DNA, RNA or mRNA, and proteome at the targeted cells or tissues, unsolicited genetic aberrations, inadequate packaging size, and immunogenicity are the principal doubts in translational gene therapeutics and genome editing [263,287]. In this context, nanoparticles (NPs) are getting considerable attention in delivering CRISPR/Cas cargo to the targeted sites due to their dynamic small sizes, excellent loading capacity, easeful penetration through the phospholipid bilayer, and other intracellular barriers that help in amplified pervasion and delivery [288]. In addition, CRISPR/Cas9 stability, biodegradation, and biocompatibility can be meritoriously accomplished by engaging NPs in clinical translation [265,289]. Nanocarriers like cargo-encumbered organic (exosome, dendrimers, micelles, nucleic acid, and liposomes) and inorganic (generally metal and magnetic-based) NPs own unique features that convey the competency to transport the captured CRISPR cargo/complexes into the cell or even within the nucleus's targeted sits [269,283,290]. The shape, size, and surface chemistry of NPs are the crucial factors that regulate nuclear or cellular uptake, biodistribution, and rapid clearance [291,292]. However, various types of NPs are available even for the detection of solid carcinoma, but very few studies have been conducted so far to deliver the NPs-based CRISPR/Cas cargo in TNBC cells that are detailed in the following sections.

#### 6.3.1 Organic nanocarriers

##### 6.3.1.1 Protein-based nanocarriers

**Fig. 6:**
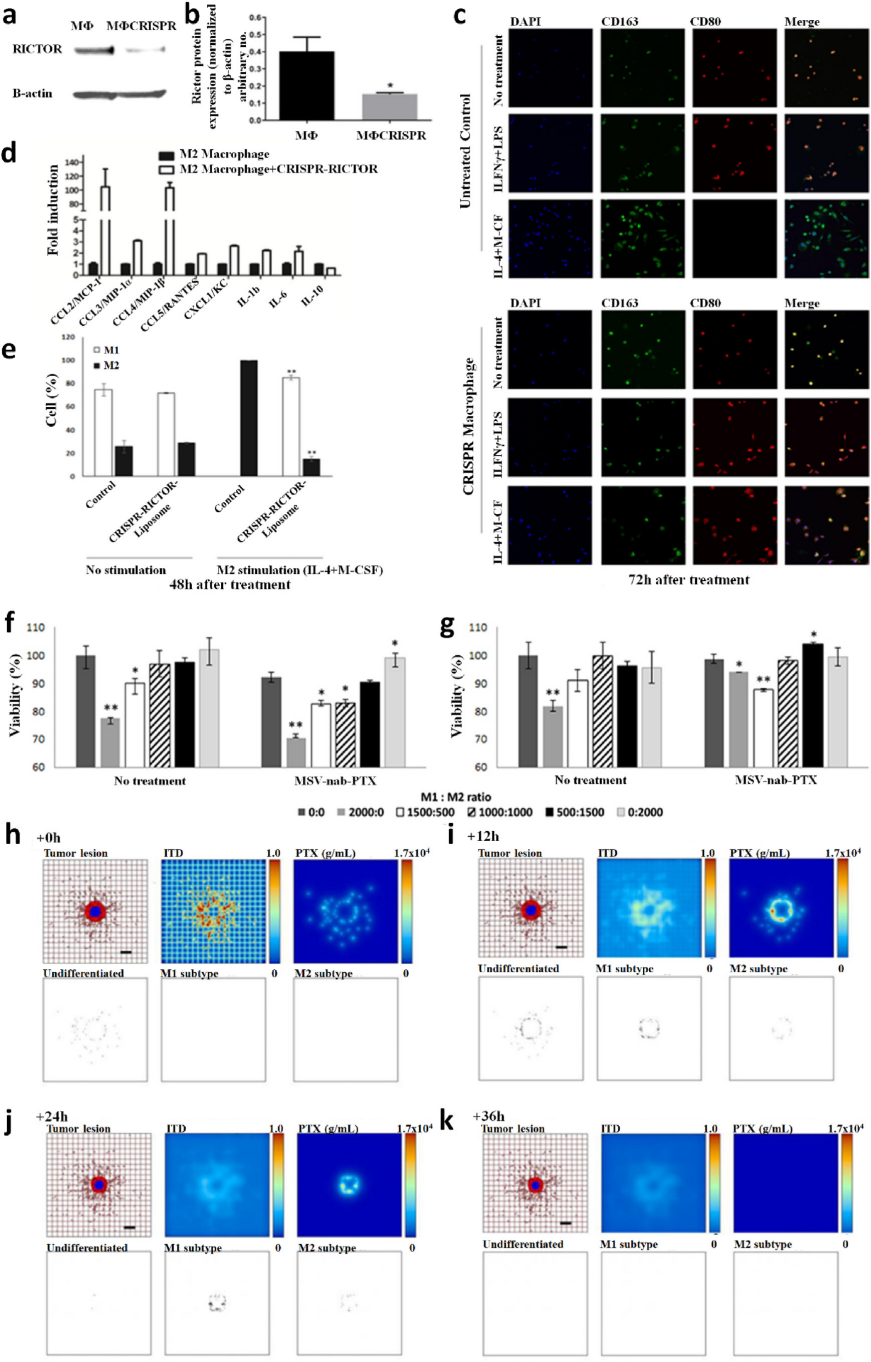
Influence of CRISPR/Cas12a sgRNA-RICTOR nanocomplex on polarized *M*2 macrophages *in vitro* and 4T1 murine model. (a) Immunoblot of RICTOR protein expression in control (MΦ) and mouse isolated macrophages treated with NP@CRISPR (MΦ CRISPR). (b) Densitometry assay for analysing band intensities of RICTOR that was normalized by β-actin (n ​= ​3). (c) Comparative analysis of control and NP@CRISPR treated macrophages, which targeted RICTOR coupled with *M*1 (IFNγ ​+ ​LPS) or *M*2 (IL-4 ​+ ​M-CSF) triggered the differentiation of macrophages. The red colour indicated the *M*1 marker, stained with CD80, and the green colour indicated *M*2 marker, stained with CD163. (d) Gene expression profile of with and without NP@CRISPR targeted RICTOR from *M*2 macrophages (n ​= ​4). (e) Quantitative stimulation assay with IL-4 and M-CSF generally prompted *M*2 differentiation. Untreated control with IL-4 and M-CSF showed bias differentiation towards the *M1* type (n ​= ​5). Scale bar ​= ​100 ​μm. (f–g) Effect of MSV-nab-PTX on *M*1:*M*2 rate of differentiation or polarization. The breast carcinoma cell's development within the tumour spheroid represents a stable *M*1:*M*2 ratio and macrophage phenotype. The NP@CRISPR targeted *RICTOR* was differentiated into *M*1 macrophages with *IFN*-γ/LPS, though *M*2 macrophages were differentiated with IL-4/M-CSF *in vitro*. The viability is represented as ratios of *M*1:*M*2 macrophages at no treatment and with MSV-nab-PTX treatment for 48 (f) and 72 ​h ​(g) with n ​= ​5. Invasion arrest assay validated macrophage polarization over 36 ​h from h-k. (h–k) Images represented tumour mass lesion after contact with an agent distressing macrophage polarization to an intermediate strength (n ​= ​280), mimicking an immune changing the *M*1:*M*2 ratio to 3.0:1, combined with MSV-nab-PTX drug-loaded nanoparticles injection. Scale bar ​= ​200 ​μm. ITD, immunotherapy drug. Reproduced by the permission of Springer Nature [243]. (For interpretation of the references to colour in this figure legend, the reader is referred to the Web version of this article.)

Albumin is a kind of protein that is vastly utilized as nanoparticles that attain innovative perspective in a new era *via* offering diverse benefits, including safety, biocompatibility, and suitable surface adjustment owing to the existence of amino and carboxylic groups [293,294]. It is the most copious protein in the blood of *Homo sapiens*, possessing almost around 67 ​kDa molecular weight and circulated 19 days as its half-life [293,295]. Additionally, it serves as a carrier for several compounds, such as copper and zinc metal ions and bilirubin, which aids in the transportation and solubilization of long hydrophobic fatty acid tail [220]. Furthermore, the albumin NPs have several binding sites that ease the integration of hydrophobic and hydrophilic drugs with the particle medium [296]. For example, dual-functional bovine serum albumin (BSA) bound with Paclitaxel-loaded mesoporous particles (MSV-nab-PTX), synthesized by hydration-extrusion method innermost crust possessed liposomes layered by CRISPR/Cas12a sgRNA-RICTOR. This complex was delivered in the 3D co-culture TME model of 4T1 mouse breast cancer cells and Balb/c 6–8 weeks old female mouse-derived tumour associated-macrophages for invasion arrest investigation that successfully switched-off the expression of RICTOR (Fig. 6a–c) resulting 75–85% macrophages polarization towards pro-inflammatory/growth-inhibition during breast carcinoma liver metastasis (Fig. 6d and e) [243]. Importantly, breast cancer liver metastasis leads to vibrant alteration in the TME of cells, especially tumour-related macrophages can be polarized into anti-inflammatory types based on TEM-mediated stimuli. Moreover, the CRISPR-treated MSV-nab-PTX complex was stabled and polarized into *M1* anti-tumour macrophage phenotype within the tumour spheroids of breast carcinoma cells (Fig. 6f and g). During the tumour mass progression, hypoxic carcinoma cells release chemoattractants, which recruit macrophages that are further separated into *M1* and *M2* subclasses, where *M1* macrophages hinder tumour growth by producing cytotoxins such as nitric oxide that became the cause of tumour mass shrinkage (Fig. 6h). However, MSV-nab-PTX uptake by the *M1* cells enhanced the cytotoxic effect on carcinoma cells without necrosis (Fig. 6i). In addition, the *M2* macrophages aided cancer progression by releasing growth factor TGF-β that elevated overall hypoxia tissues proliferation (Fig. 6j), while MSV-nab-PTX-encapsulated payload treatment decreased the tumour cell survival and increased tumour cells shrinkage (Fig. 6k), thus proving the effectiveness of protein-based nanocarrier in the CRISPR/Cas TNBC therapy. Also, this approach has overcome the clearance from circulation and transport barriers *in vivo* and *in vitro* by phagocytic uptake that will be helpful, potentially, in immunotherapy and post-chemotherapy.

##### 6.3.1.2 Lipid-based nanocarriers

The lipid-based CRISPR/Cas delivery vehicles have been conjectured since 2017 [232] to offer improved cargo carriage into the targeting tumour cells, combat antagonistic impact, and aid in the problem of multidrug confrontation [276]. The lipid molecules are devouring amphiphilic characteristics along with the hydrophobic tail and polar head that are allied between the two domains, systematically categorized into ionizable, cationic, and neutral lipids. The ionizable lipids are mostly easily protonated at the least pH value but remain amphipathic or impartial at biological pH. Further, the pH-responsive character of ionizable lipids aids favourable sgRNA delivery *in vivo*, since neutral lipids rarely interact with the negatively charged membranes of blood cells, thus increasing the biological compatibility of lipid nanoparticles (LNPs) [291,297]. Conversely, cationic lipids possessed a permanent positive charge on the head group [277]. Nonetheless, lipid or lipid-like NPs can be fabricated through diverse materials that provide an extensive diagnostic and therapeutic application for aggressive carcinoma [276] as TNBC therapy and its research. It has excellent targeting capability, biocompatibility, and biodegradability, especially as a nanocarrier, as well as a self-assembly nano/micro emulsified delivery system for CRISPR/Cas [289]. Also, many lipids or lipid-like delivery vehicles, such as nanolipogels (NLGs) and nanoliposomes (NLPs), have been aimed for mRNA, oligonucleotides, and plasmid-derived DNA delivery because of the substantial lessening in immune reaction stimulations, stumpy genotoxicity, the safety of CRISPR/Cas9-sgRNA complex from endonucleases, and rapid renal evacuation. Thus, synthetic lipid NPs believe to be superlative nanocarriers for CRISPR/Cas9-based gene editing into the TNBC cells [233].

The NLPs and NPs are the two foremost vehicles for CRISPR cargo delivery used to regulate effective cargo-loaded circulation and release [283]. This dual structures-based system can be engineered using indistinguishable morphology and surface charges, despite the characteristic transformations existing between their particle elasticity and architecture [192]. NPs retain amorphous or crystalline solid-structured inorganic materials or polymers [298], while NLPs are composed of an aqueous core encapsulated by phospholipid bilayer [291]. However, both NPs and NLPs have size-reliant possessions, prominently distinct from the characteristic of the majority of substances. In addition, somatic signals are well-known for administering cell and NP communications [289]. The particle size of about 100 ​nm competently escapes the nuclear phagocytosis machinery, thereby extending blood circulation. Also, the surface chemistry of NPs distresses transmission lifetime and biological conveyance [298]. The cationic system displayed considerably non-specific cellular uptake and advanced serum protein captivation compared to the anionic or impartial particle system [287].

**Fig. 7:**
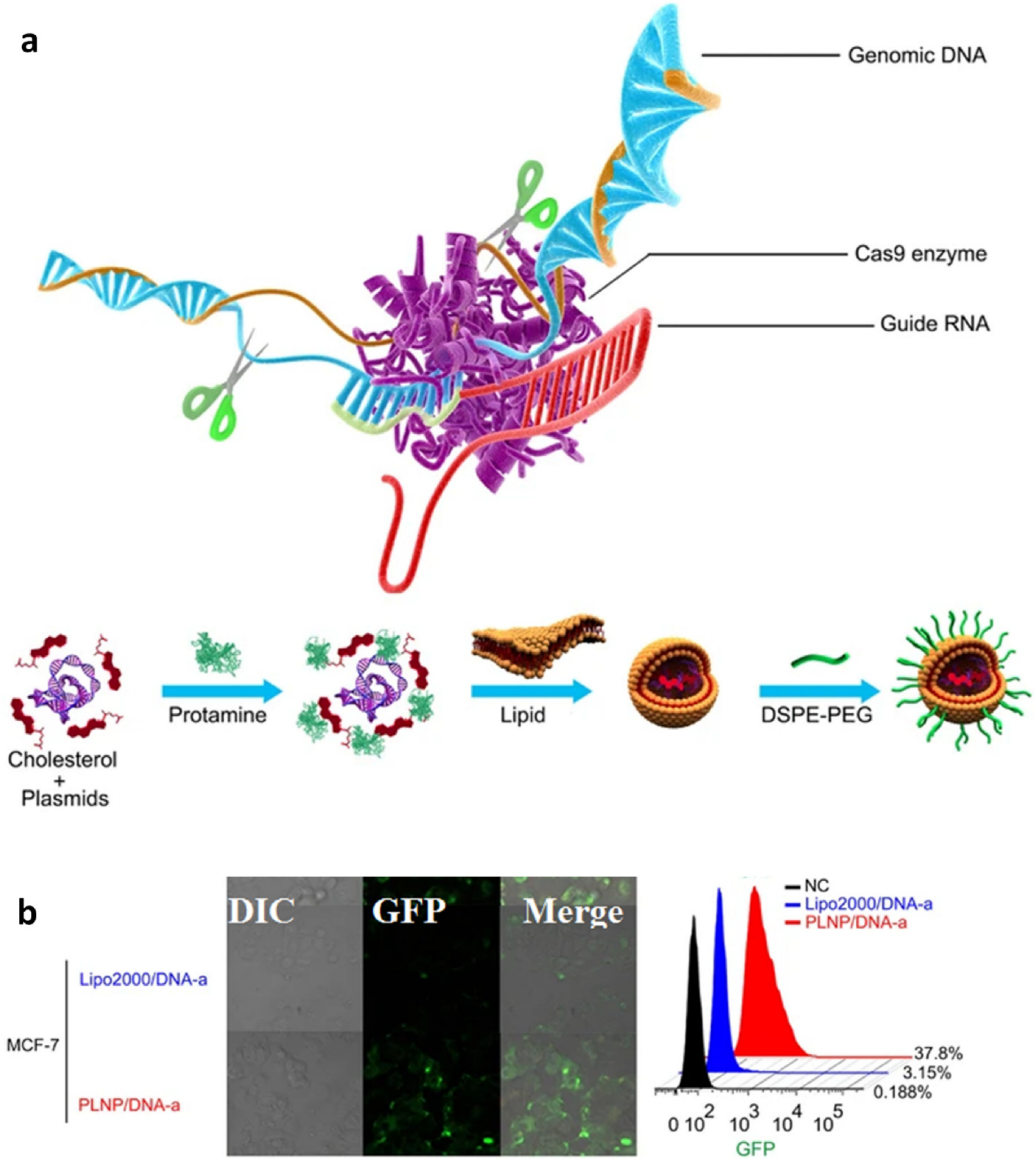
Schematic illustration of CRISPR/Cas9 system delivered to edit Polo-like kinase 1 (*PLK-1*) gene in MCF-7 ​cell lines. (a) The packing and encapsulation method of the CRISPR/Cas9-sgRNA plasmid through protamine, chondroitin sulphate, dioleoylphosphatidylethanolamine (DOPE), 1,2-distearoyl-sn-glycero-3-phosphoethanolamine-N-methoxy polyethylene glycol-2000 (DSPE-PEG), and 1,2-dioleoyl-3-trimethylammoniumpropane (DOTAP). (b) The confocal laser scanning microscopy images and flow cytometry showed CRISPR-@NPs complex transfection efficiency *in vitro* at 48 ​h. The TNBC cell line (MCF-7) was treated with Cas9-sgPLK-1a (NC), Lipo2000/DNA-a (Lipofectamine 2000 encapsulated Cas9-sg *PLK1* plasmid-free DNA), and PLNP/DNA-a (Cas9-sg *PLK1* plasmid DNA encapsulated in cationic lipid shell, modified by polyethylene glycol phospholipid). Reproduced by the permission of Springer Nature [232].

Apart from NLPs-NPs, NLGs-NPs hybrid system consisting of alginate hydrogel encapsulated with phospholipid bilayer or liposome offer tuneable characteristics and surface charge proved as an idyllic system to investigate both *in vivo* and *in vitro* tumour accumulation and cellular uptake as particle elasticity alter and induce capability to bind cellular surface receptors and squeeze through plasma membrane located channels [299]. Numerous liposome-derived carriers, for example, Myocet®, Doxil®, and AmBisome®, have been commercially manufactured [263]. Since CRISPR/Cas-associated sgRNA is highly anionic, for efficient component delivery, the superficial layer of NLPs or NLGs ought to be cationic to enhance the absorption ratio of charged particles. Despite the encapsulation of Cas9 endonucleases in the LNPs, charge neutralization between Cas9 and negatively charged protein is essential. For that synergy, diverse kinds of agents have been assessed, including polylysine, polyethyleneimine, chitosan, polyethylene terephthalate, powder bed fusion, TAT-peptide, transIT®-LT1, FuGENE® 6, and nuclear localization signal peptide together with gold, magnetic, carbon NPs with variable surface advancement. Among them, Cas9-sgRNA along with polo-like kinase-1 (*PLK-1*) encapsulated within an innovative cationic polyethylene glycol (PEG) phospholipids NPs delivery system ascertained to be better than the incredulous CRISPR's insufficient transfection efficacy (Fig. 7a). However, *in vitro* transfection efficiency was about 37.8% in the MCF-7 breast cancer cell lines when compared to the commercial transfection agent as lipofectamine2000, which showed 3.15% transfection (Fig. 7b) [232].

**Fig. 8:**
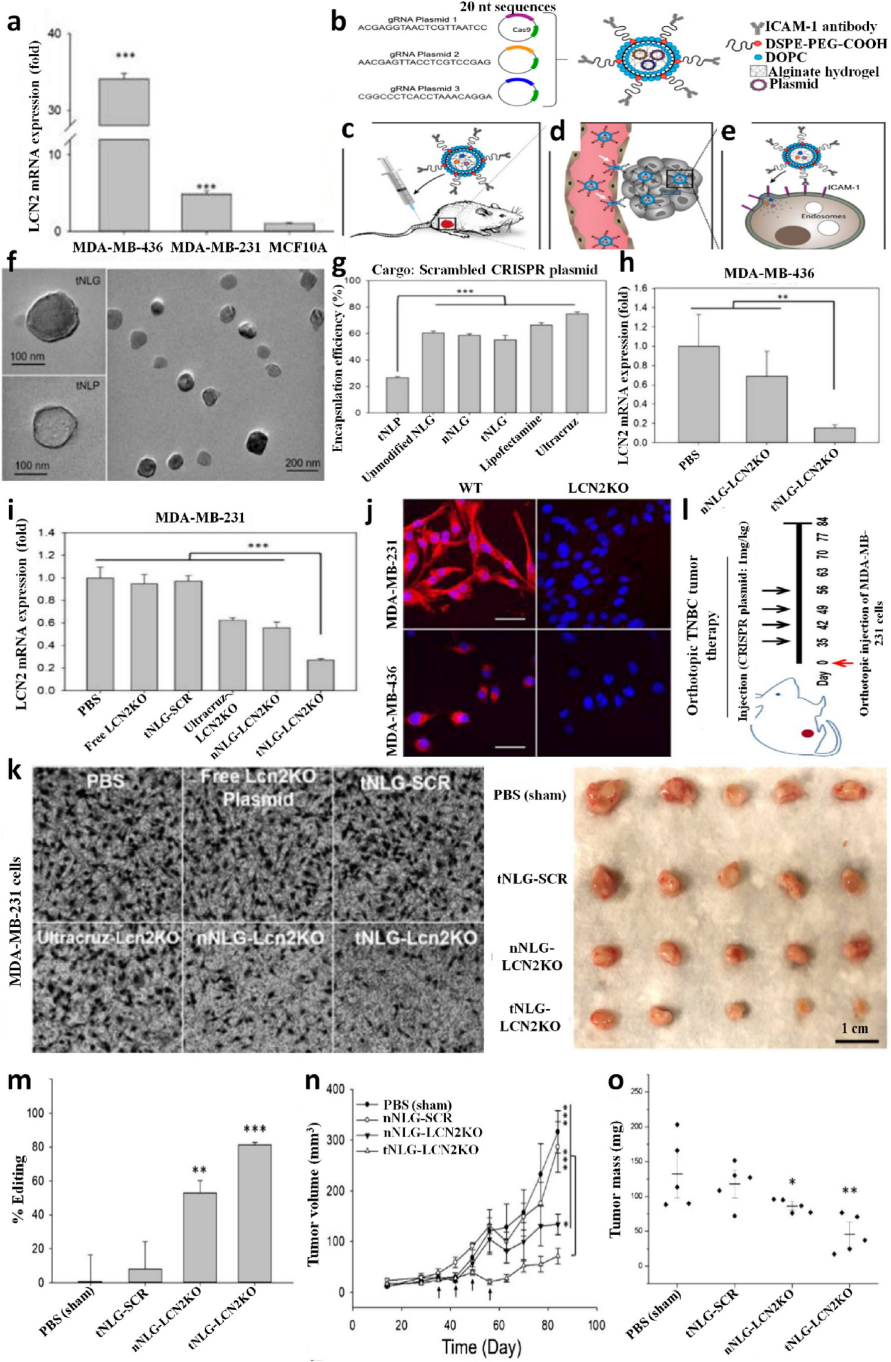
The CRISPR encapsulated in nanolipogel (tNLG)-targeted silencing of lipocalin 2 (*LCN2*) gene significantly attenuated TNBC tumour growth. (a) Overexpression of *LCN2* oncogene in human TNBC cells. (b) Schematic representation of 20 nucleotides (nt) sequences of sgRNA-bearing plasmids along with surface modification and targeting components. (c) NP@CRISPR intervenors injection can be effectively engineered the TNBC genome *in vivo*. (d) Proposed tNLG complex can cross the endothelial barrier. (e) The CRISPR-bearing plasmids were directly delivered into the TNBC cells cytosol through ICAM1-direacted plasma membrane pathway. (f) TEM images of tNLG and tNLP (nanoliposome without a hydrogel core). (g) Encapsulation efficacy of prepared lipid-based nanoparticles and commercial control coupled with CRISPR plasmids. (h–i) *in vitro LCN2* silencing efficacy was shown in distinct TNBC cell lines. (j) Protein expression profile before and after *LCN2*-CRISPR knockout was detected by IF staining. (k) Images of transwell migration analysis of MDA-MB-231 ​cells together with NP@CRISPR targeted *LCN2* knockout. (l) Image of excised tumour at 48 days from female nude mice (n ​= ​5) treated with PBS (phosphate-buffered saline), tNLG-SCR (tumour-targeted nanolipogel encapsulated scramble CRISPR plasmid), nNLG-*LCN2* KO (IgG conjugated nonspecific nanolipogel-lipocalin 2 knockout), and tNLG-*LCN2* KO (ICAM1 antibody-conjugated tumour-targeted nanolipogel-lipocalin 2 knockout) at various time with 1 ​mg plasmid/kg MDA-MB-231 ​cells bearing mice. (m) tNLG-based genome engineering efficacy was measured by qRT-PCR. (n) Weekly tumour progression measured as the volume of tumour. (o) Weight of tumour mass at 84 days. Reproduced by the permission of PNAS [233].

In another example, CRISPR/Cas9 direct interference in the Lipocalin 2 (*LCN2*), also recognized as gelatinase-associated lipocalin, a key secretory protein of N2 neutrophils, a mediator of metabolic pathway, and intestinal system [300], exhibited a significant decline in TNBC cells growth. The *LCN2* expression is positively related to basal-like immunoactivated and immunosuppressed type, which are 50–88% of all TNBC types and significantly expressed in patient's urine and tissues (Fig. 8a). Also, ICAM-1 antibody-guided, anionic, deformable, core-shell tumour-targeting NLG (a mixture of 1, 2-dioleoyl-sn-glycero-3-phosphocholine and 1, 2-dioleoyl-sn-glycero-3-phosphoethanolamine-N-(carboxy (polyethylene glycol)-2000)) based NPs was applied as a potent carrier system for three siRNA-Cas9 plasmids (Fig. 8b and c) because cationic nano-devices suffer significantly in clinical translation. The cationic LNPs form electrostatic complexes with an anionic CRISPR/Cas9 system that ubiquitously sabotages plasma membrane and grounds unadorned cytotoxicity (Fig. 8d and e). Moreover, engineered tNLG were 110 ​nm in diameter in size and exhibited very least polydispersity (0.2) (Fig. 8f). Also, tNLG encapsulation efficiency was almost 80% as compared to commercially available liposomes (20%) (Fig. 8g).

The *in vitro* tNLG-CRISPR/Cas9 (tNLG-*LCN2*KO) complex-mediated 80% silencing of *LCN2* oncogene in MDA-MB-436 and MDA-MB-231 TNBC cell lines verified the site-specific efficacy and least off-targeting knockout impact (Fig. 8h and i). Moreover, without nanovehicle (Free *LCN2*KO), CRISPR/Cas9 inhibited *LCN2* mRNA expression only 30–50% (Fig. 8i). Further, the tNLG-CRISPR/Cas9-based silencing reduced Lcn2 protein synthesis (Fig. 8j) and transmigration of TNBC cells (Fig. 8k). While MDA-MB-231 tumour-bearing female mice model confirmed 81% inhibition of TNBC progression, 77% tumour volume, and 69% mass in comparison with untreated one (Fig. 8l-o) [233]. Furthermore, tNLG-CRISPR/Cas9 complex exhibited excellent capability to escape efficiently tumour endothelial obstruction (67%) by its high deformability design and selection of alternative pathways such as receptor-based membrane fusion path. Therefore, the CRISPR/Cas9-tNLGs-based system is safe, effective, and precise for TNBC targeted gene therapy. Meanwhile, LNPs have poorer immunogenicity than virus-based vectors; thus, it can assist as a possible substitute for *in vivo* genomics. In summary, LNPs or lipid-like NPs can be measured as an auspicious delivery vehicle to accelerate the transfection capability of CRISPR/Cas9 gene engineering and successively therapeutic impact in TNBC metastasis. It is prominent that previous research utilized large-size CRISPR pDNA to accomplish gene silencing. Despite the cited benefits, the foremost matter related to plasmid DNA is extraordinary intensities of unintentional gene edits, poor endosomal discharge, and delivery efficacy that relatively limited *in vivo* application of this approach.

##### 6.3.1.3 Polymer-based nanocarriers

Polymer-based NPs are nanosized materials produced by synthetic or natural biocompatible and decomposable monomers or polymers [287]. They can be synthesized by solvent displacement or diffusion emulsification [301], ionic gelation [302], nanoprecipitation [26], and microfluidics [303]. Based on polymer characteristics and structure, diverse types of multiple, dual, and single CRISPR systems can be engineered inside the NPs core, chemically linked or conjugated, entrapped in the matrix of polymer, or NPs-bound surface polymer that can be delivered and released payload *via* bulk or superficial erosion, diffusion and inflammation [192]. Polymer-based polymeric NPs, micelles, and dendrimers have been extensively utilized for pDNA, mRNA, oligonucleotides, and nucleic acid delivery and impressively dealt with a distinctive improvement of biological compatibility. It signifies prodigious packaging capability and noteworthy synthetic properties in alleviating desired gene encoding Cas9-sgRNA complex counter to serum-tempted accumulation in TNBC [304]. Moreover, stability, composition, responsivity, superficial charge, kinetics, and loading efficiencies can be accurately restrained [305].

The general forms of polymeric NPs include nanospheres (solid matrix), and nanocapsules (cavities around polymeric shell), which are further divided according to the exterior appearance as in dendrimers, polymersomes, and micelles [287]. The polyamidoamine (PAMAM) or dendrimer is highly branched and high density by possessing primary amine on its periphery, which facilitates a tight junction between nucleic acid and polymer. At the same time, the functional group on its exterior recruits contrast agents or biomolecules over the dendrimer surface [289,306]. However, CRISPR cargo can be encumbered on its interior. Dendrimers have dynamic tuneable 3D architecture like shape, size, mass, and superficial chemistry such as charged polymer polyethylenimine (PEI) [291]. Therefore, numerous dendrimer-centered products are presently running in clinical translation as gene therapy delivery vehicles, contrast and theranostics agents, and topical gels [235,307,308]. Additionally, the charged dendrimer can produce non-dendrimer polymer such as polyelectrolytes having a reiterating electrolyte group, which define their charge variability with pH that improves polyelectrolyte's mucosal transport [309] and bioavailability [310]. Polymersomes are synthetic vesicles usually produced by the amphiphilic block of copolymers that are similar to liposomes and frequent locally receptive but evidenced to have better permanency and payload-retaining efficacy [311], thus making them efficient means of transport for CRISPR/Cas9 delivery in the nucleus at the targeted site. Some applicable polymersomes include hydrophilic blocks of poly (ethylene glycol), and poly (2-methyloxazoline), and hydrophobic blocks of poly (dimethylsiloxane), poly (caprolactone), poly (lactide), and poly (methyl methacrylate). The polymeric micelles are typically self-assemble copolymers of the responsive block, composed of amphiphilic nanospheres core layered by hydrophobic shell through Van der Waals forces, serving to guard CRISPR-sgRNA cargo along with prolonging circulation time, that precludes rapid evacuation under the guidance of reticuloendothelial system (RES) [192]. Moreover, its partial-solid nonpolar core is devised by a biologically demeaned polymer likes polylactic-*co*-glycolic acid, poly (β-caprolactone), and poly (l-lactide). In addition, micelle polymer measures around 10 ​nm–100 ​nm in diameter, perfect for reinforcing various hydra-insoluble gene engineering.

**Fig. 9:**
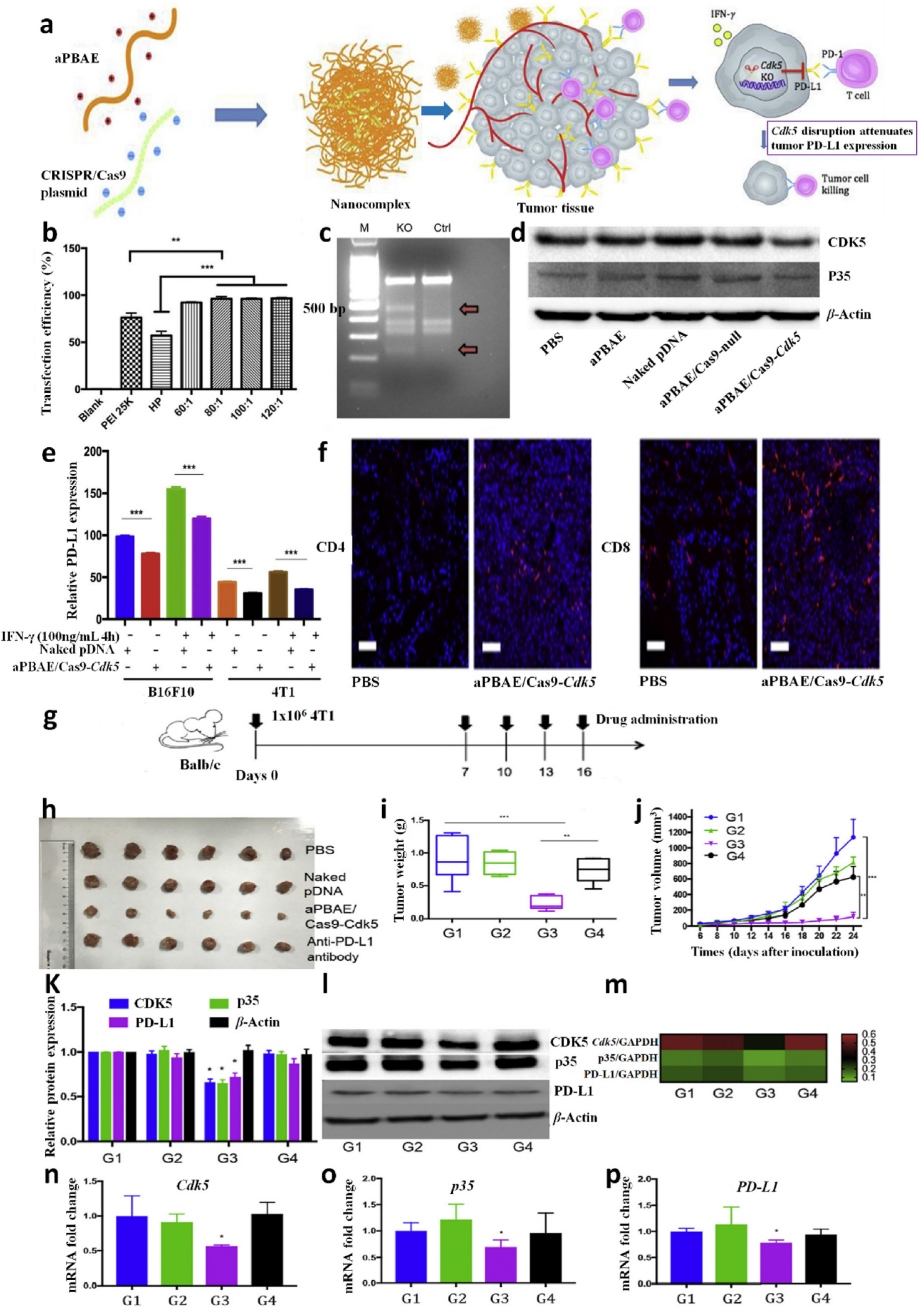
The TNBC tumour growth inhibition by aPBAE@Cas9-*Cdk5* KO *in vitro* and 4T1 murine model. (a) Schematic representation of biodegradable cationic polymer, aPBAE@Cas9-*Cdk5* knockout-based TNBC therapy. (b) Transfection efficacy of aPBAE/pMax-GFP (green fluoresce proteins) in TNBC cells. (c) The T7E1 cleavage assay was directed to detect aPBAE@Cas9-based silencing of *Cdk5* gene in B16F10 murine cells. M, KO and Ctrl stand for DNA leader, knock out, and control, respectively. (d) The CDK5 and co-activator p35 proteins expression profile after distinct treatment by western blot. (e) Relative expression of *PD-L1* was measured after the transfection of IFN-g, naked plasmid DNA (pDNA) and aPBAE/Cas9-mediated *Cdk5* knockout in the 4T1 cells (n ​= ​4). (f) Immunofluorescences image exhibited aPBAE@Cas9-*Cdk5*KO-mediated PD-L1 reduction triggered CD4^+^ and CD8^+^ T-cell infiltration. Scale bar at 100 ​μm. (g) Treatment timeline of CRISPR/Cas9-dependent PD-L1 attenuation by intratumoral administration of aPBAE@Cas9-*Cdk5* at 7, 10, 13 and 16 days in 4T1 murine model. (h) Tumour excised from Balb/C female mouse (n ​= ​6) treated with PBS (phosphate-buffered saline), naked pDNA (plasmid DNA), aPBAE/Cas9-*Cdk5* (cationic copolymer AMP-modified poly-beta amino ester -CRISPR/Cas9-cyclin-dependent kinase 5), and anti-PD-L1 antibody. (i) Tumour weight reduction in grams after G1 (PBS), G2 (naked pDNA), G3 (aPBAE/Cas9-*Cdk5*), and G4 (anti-PD-L1 antibody) treatment (n ​= ​6). (j) Tumour growth reduction after various treatments in mm^3^ (n ​= ​6). k | CDK5, P35, PD-L1, and *β*–Actin proteins expression quantification and, (l) qualification by western blotting that were derived from phosphate buffer saline (PBS) (G1), pDNA (G2), aPBAE@Cas9-*Cdk5* (G3) and anti-PD-L1 antibody (G4) treated breast carcinoma bearing mice (n ​= ​3). (m) Heat map of mRNA expression of tested genes compared to GAPDH house-keeping gene was generated by qRT-PCR. (n) Alteration of *Cdk5* mRNA expression level, (o) p35 mRNA expression level, and (p) *PD-L1* mRNA expression level from PBS (G1), pDNA (G2), aPBAE@Cas9-*Cdk5* (G3) and anti-PD-L1 antibody (G4) treated breast carcinoma bearing mice (n ​= ​3). Reproduced by the permission of ELSEVIER [26]. (For interpretation of the references to colour in this figure legend, the reader is referred to the Web version of this article.)

Inclusively, polymer NPs are absolute aspirants for CRISPR-based gene editing due to their biomimetic, biocompatible, biodegradability, hydra soluble, and shelf-stable properties [271]. In addition, the surface modification is very easy for targeted delivery that make them beneficial in TNBC translation. However, the detriments of the polymer include an augmented hazard of particle accumulation and related noxiousness. Very few polymers are approved by FDA for medical usage, although these nanocarriers are presently experiencing challenges in scientific trials for TNBC. For example, *PD-L1*, a crucial immune checkpoint cell surface receptor direct blockage *via* efficient and novel CRISPR/Cas9 system delivered through cationic copolymer AMP-modified poly-beta amino ester (aPBAE) (Fig. 9a) with 95% transfection efficacy in 4T1 carcinoma cells at 80 ​wt ratio. Also, the cationic copolymer showed higher transfection as compared with conventional vectors even when weight ratio beyond 80, thus proved as versatile gene delivery vector to edit genome (Fig. 9b). The targeting efficacy of cationic copolymer/CRISPR system was as higher because it produced only two bands, one is 200 ​kDa and other is 400 ​kDa (Fig. 9c). Further, cyclin-dependent kinase 5 (*Cdk5*), and p35 proteins expression (Fig. 9d) and PD-L1 relative expression was significantly decreased after aPBAE conjugated Cas9-Cdk5 knockout in the TNBC cells (Fig. 9e). Notably, aPBAE conjugated Cas9-*Cdk5* based NPs has provoked strong T cell-facilitated immune reactions in the cancer microenvironment, where the CD8^+^ T cells population was considerably improved on the other hand governing T cells population was declin ed, provide the favourable stratagem for the TNBC antitumour clinical treatment and gene therapy (Fig. 9f). Moreover, *in vivo* antitumour effect of *Cdk5* gene manipulation by intravenous injection silent 95% genetic expression of *Cdk5*, which decreased 75.7% tumour weight, 89.6% tumour volume, protein expression profile, and messenger RNA expression, than anti-PD-L1 antibody and PBS control group (Fig. 9g-p). Further, aPBAE conjugated Cas9-*Cdk5*-NPs induce the expression of granzyme B and *IFN-γ* in 4T1 mice, which showed elevation of cytotoxic T-lymphocytes (CTL) retort in TME, leading to effective TNBC immunotherapy that efficiently modulates immunosuppression in TME [26].

#### 6.3.2 Inorganic nanocarriers

Several types of rigid nano-sized inorganic materials, including black phosphorus, carbon (C) nanotubes, calcium carbonate (CaCO_3_), gold (Au), graphene, silica (SiO_2_), and iron oxide (Fe_2_O_3_) NPs are used for nanoformulation due to their strong potential for CRISPR/Cas safe delivery, drug distribution and imaging implication. These inorganic NPs are specifically engineered to have varied types of the nanostructure, surface morphology, sizes, and geometries [312]. Thus far, gold NPs have fascinated significant consideration to emerging pioneering CRISPR/Cas9 carrier structures for targeting tumours in the murine model with 97% encapsulation efficacy and 79.4% release efficiency [276]. They are the utmost well-researched nanostructures that are exquisitely exploited in innumerable forms, including nanostars, nanospheres, nanocages, nanorods, and nanoshells [313], and widely used as effective breast therapy [265]. In addition, inorganic nanocarriers have inimitable magnetic, physical, optical, and electrical characteristics because of their intrinsic core quality of the material [314].

Evidently, gold nanocarriers own free electrons at their outermost shell that constantly oscillate in a frequency-dependent manner according to the shape, size, and magnitude, benevolent the photothermal character [222]. Further, easeful functionalization concedes extra delivery abilities and surface modification properties [313]. For instance, gold NPs are significantly incorporated in advancement of cell-free DNA-based breast carcinoma diagnosis that was suffered due to effective site-specialized primer design and false-positive signals limitations, prominently overcome *via* CRISPR/Cas-dependent fluorescent biosensing system and CRISPR/Cas12a@Au nucleic acid-based amplification system (free of fluorescence-based). The fluorescence-free system is superseded over other and mainly based on metal-enhanced fluorescence generated by plasmonic nanoparticles. Upon triggering, CRISPR/Cas12a degrade the cfDNA into single-stranded cfDNA that feasibly monitor through colour-changed chemistry (purple to red) of gold NPs. Also, the CRISPR/Cas12a@Au system can successfully exploit to detect *BRCA-1* gene mutations within 30 ​min [53].

**Fig. 10:**
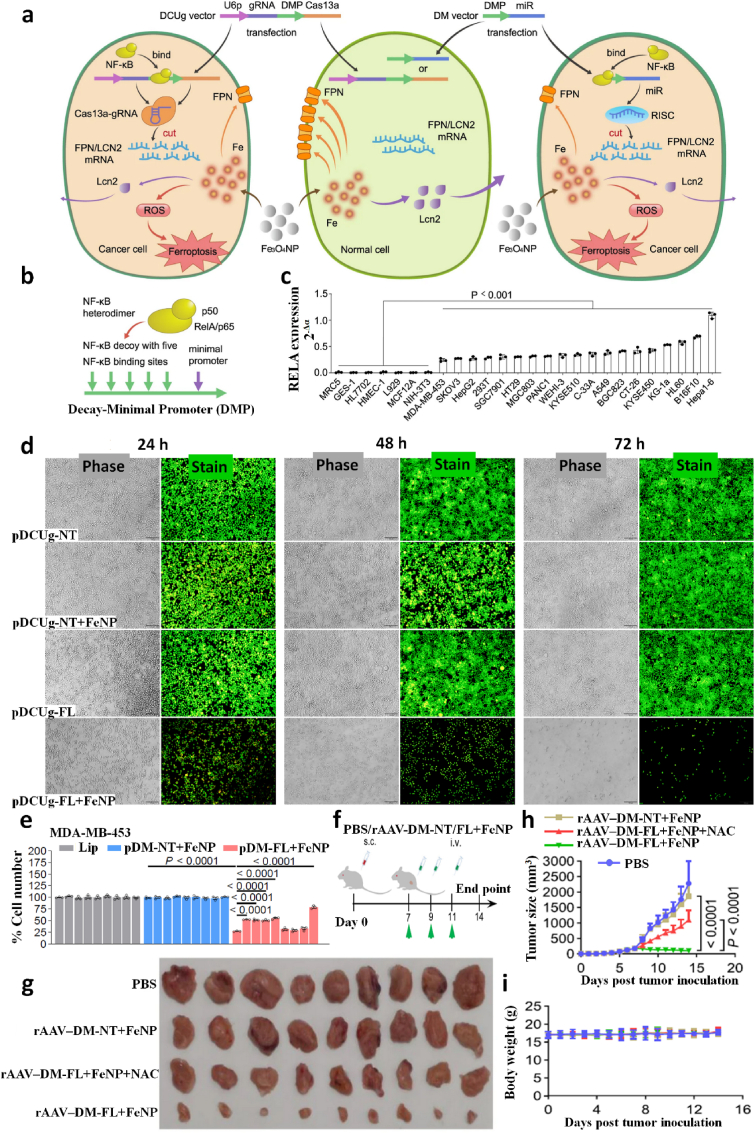
The CRISPR/Cas13a and miRNA-dependent GIFT together with magnetite (Fe_3_O_4_) nanoparticle-based dual silencing of Lipocalin 2 (*LCN2*) and Ferroportin-1 (*FPN*) genes. (a) Schematic illustration of CRISPR/Cas13a-GIFT principle and *NF-κβ* gene expression in cells. (b) Decay minima promotor (DMP) and *NF-κβ* gene complex. (c) *NF-κβ* RELA/P65 expression was detected in various cancer cell lines by qPCR (n ​= ​3). (d) Fluorescence images of *in vitro* GIFT facilitated TNBC tumour cells killing. The MDA-MD-453 ​cells were treated with several plasmids for 24 ​h, then 50 ​μg/mL of FeNPs were supplied for another 72 ​h and stained with ethidium bromide and acridine orange. Scale bars ​= ​100 ​μm. (e) TNBC cell viability assay investigated *via* Cell-Titer-Glo 2.0. The MDA-MB-453 ​cells were treated with lipofectamine (Lip), pDM-NT, and pDM-FL for 24 ​h. The transfected cells were coincubated with inhibitors and FeNP for 48 ​h (n ​= ​3). (f) Scheme of *in vivo* NP@CRISPR-based knockout in mice by i.v. injection at a distinct timeline. (g) *In vivo* GIFT impact in mice at 7, 9, 11, and 14 days. Image showed tumour mass growth with PBS (phosphate-buffered saline), rAAV-DM-NT-FeNP (recombinant adenovirus associated virus-DMP miRNA-target no transcript-DMSA-coated Fe_3_O_4_ nanoparticles), rAAV-DM-FL ​+ ​FeNP ​+ ​NAC (recombinant adenovirus associated virus-DMP miRNA-target *FNP* and *LCN2* ​+ ​DMSA-coated Fe_3_O_4_ nanoparticles ​+ ​*N*-acetylcysteine), and rAAV-DM-FL ​+ ​FeNP (recombinant adenovirus associated virus- DMP miRNA-target *FNP* and *LCN2*-DMSA-coated Fe_3_O_4_ nanoparticles) treatments in tumour mass. (h) Mice tumour mass and (i) Average body weight after PBS, rAAV-DM-NT ​+ ​FeNP, rAAV-DM-FL ​+ ​FeNP ​+ ​NAC, and rAAV-DM-FL ​+ ​FeNP treatments. DCUg, DMP-Cas13a-U6-gRNA, DM, DMP-miRNA; FeNP, DMSA-coated Fe_3_O_4_ NPs; FL, dual knock out of *FNP* and *LCN2* genes; gRNA, guide RNA; NT, target no transcript; pDCUg, Cas13a sgRNA coupled with U6 promoter; pDM, a plasmid with DMP; U6p, U6 promoter; DMP, NF-κB decoy-minimal promoter. Reproduced by the permission of Springer Nature [280]. (For interpretation of the references to colour in this figure legend, the reader is referred to the Web version of this article.)

Another inorganic nanocarrier, such as carbon, generating noticeable attention about the expansion of improved carrier delivery systems due to the governable topographies such as wider surface area concerning volume ratio, simple surface alteration to upsurge uptake, tuneable size, immunologically inactive surface, and excellent stability in an internal biological setting [315]. Similarly, iron oxide is a very prevalent examined material for inorganic nanocarrier production, and the majority of them are FDA-permitted nanomedicines or vehicles for gene delivery [269,316,317]. Also, magnetic iron-based gene-editing tools are compounds of maghemite (Fe_2_O_3_) or magnetite (Fe_3_O_4_) that retain superparamagnetic characteristics with confined structures or sizes and have shown triumph as gene delivery systems and contrast agents in thermal-based cancer therapy [292]. For example, two tumour-inducing genes such as ferroportin (*FPN*) and lipocalin2 (*LCN2*) were knockdown by Cas13a/U6-gRNA with a *NF-ĸβ* expression controlled “minimal decay promoter” (DMP) (Fig. 10a and b), coated with magnetite nanocarrier that significantly enhanced the ferroptosis in MDA-MB-453 ​cells line (Fig. 10c). Hence, reducing tumour growth for a longer period in both *in vitro* and murine-based *in vivo* model, proving as an idyllic nonviral delivery vehicle for direct CRISPR-based gene engineering [280].

Mechanistically, gene interfering vector (GIV) and iron nanoparticle (FeNPs) were grafted with DMP and downstream effectors genes such as *FPN* and *LCN2* to export Fe ions that induced ferroptosis (iron-dependent non-apoptotic cell death), which significantly killed MDA-MD-453 ​cells *in vitro*. On the other hand, negative control plasmid (pDCUg-NT), iron nanoparticles alone (pDCUg-NT ​+ ​FeNP), and silenced genes without iron nanoparticles (pDCUg-FL) did not affect tumour cell viability (Fig. 10d). Moreover, GIFT-induced ferroptosis was persistently effective even in the presence of *N*-acetylcysteine (analog of cysteine), bafilomycin A1 (autophagy inhibitor), deferoxamine (iron chelator), ferrostatin-1, and liproxstatin-1 (ferroptosis inhibitors), necrostatin-1s (necrosis inhibitor), and ZVAD-FMK (apoptosis inhibitor) (Fig. 10e). Conversely, the TNBC cell viability can be significantly rescued through coinduction of deferoxamine, ferrostatin-1 and *N*-acetylcysteine (Fig. 10e). Similarly, GIV and FeNPs-based GIFT applications not only decreased the size and growth of tumour mass (Fig. 10f–h), but also did not cause changes in body weight and toxicity in major organs of mice (Fig. 10i). Additionally, rAAV-DM-FL ​+ ​FeNP intravenous injection positively improved and lengthen the overall survival period of mice.

Other common nanocarriers, including mesoporous silica, and calcium phosphate (Ca_3_ (PO_4_)_2_), both have been utilized efficaciously for safe gene and drug transfer into the target site [318]. Moreover, quantum dots, characteristically prepared by semiconducting nanomaterials such as silicon (Si), are exceptional NPs used principally for *in vitro* imaging implementation. However, they also have the potential for *in vivo* therapeutic trials [319]. Currently, silver, cadmium, indium, silicon, and carbon quantum dots are widely applied in cancer genomics due to their higher degree of core cytotoxicity, variable luminescent characteristics, and diverse subcellular fate [320].

In general, struggles to practice nonviral gene editing and transfection strategies have been rigorously restricted by the low delivery efficiency through the plasma membrane of the cell. Correspondingly with inorganic nanocarrier, the efficacy of genome editing is improved when compared with lipid-based formulations and native Cas9 systems. Furthermore, plasmonic or radioactive, photothermal, and magnetic characteristics of inorganic nanocarriers make them ideal for imaging, diagnostics, and photothermal application in cancer genetics. Most inorganic NPs have virtuous stability and biocompatibility and, therefore, block niche applications that need inaccessible properties through organic nanocarriers. Nonetheless, they are inadequate in clinical translation due to the toxicity, and poor solubility disquiets, and specifically heavy metal-based formulations [269,321].

## 7 Design strategies for effective CRISPR-nano engineering in TNBC

The plasmid-based approach is evident in TNBC pre-clinical applications due to stability, ease of synthesis, low cost and multiple targeting options. However, efficient packaging into the single delivery vehicle and transportation are tremendous tasks [233]. Mechanically, the Cas9 endonuclease and sgRNA-bearing plasmid can target gene/genes by cellular internalization, and upon release, the Cas9 plasmid enters the nucleus for further processing of sgRNA. After transcription, sgRNA and mRNA are delivered to the cytoplasm for translation and then translated RNP (Cas9 protein and sgRNA) redelivered into the nucleus for CRISPR-assisted gene engineering [43]. Importantly, lingering plasmid DNA in the host genome may cause undetectable mutagenesis or off-target impact, leading host immune retort issues.

Systematically, nonviral-mediated plasmid delivery has much more benefits than virus-mediated delivery but suffers due to low transfection efficacy. In this respect, cationic lipid nanocarriers may aid low toxicity and easeful endolysosomal escape [232]. In addition to delivery, it has a complicated process, including transcription, translation and re-translocation in the nucleus, delay or decrease gene engineering precision. Furthermore, using the HDR mechanism by pCas9 plasmid is far from practical, especially *in vivo* trials [39]. Also, double-stranded Cas9 plasmid can integrate at any site of the host genome and induce constant expression of Cas9 that may elevate off-target impacts. It is suggested to induce silent hindering mutation either in the sg RNA target sequences or PAM sequences that prevent Cas9 plasmid from inducing undesirable mutation repeatedly within the target site. Moreover, before *in vivo* and *ex vivo* studies, Sanger sequencing and tracking of indels by decomposition (TIDE or TIDER) can be used to identify design inaccuracies in TNBC *in vitro* model [231].

The mRNA-based system is greatly easeful, less time-consuming and precise where Cas9 mRNA directly delivers into the cytoplasm after successful endolysosomal escape for functional protein translation and then RNP complex transfer in the nucleus for subsequent gene engineering. This system has a shorter half-life, lesser off-target impact, and faster expression. Recently, approval of the Cas9 mRNA-based vaccine will significantly enhance its importance in clinical application. However, very few studies have utilized this system due to the rapid enzymatic degradation of mRNA. Further, Cas9 mRNA is typically complicated due to heterogeneous action mechanisms. Its mRNA with sgRNA uses the NHEJ mechanism for gene editing, while donor DNA with sgRNA implies HDR mechanism, making its intracellular transportation intricate. Its considerable variations in administration track hamper the synthesis/production for customized delivery. In this case, nanomaterials-mediated delivery is preferential due to nano protection against nucleases-based degradation [39]. However, a more sophisticated method such as systemic administration is better than others, where the least chances of biological hindrance are foreseen. Despite the delivery obstacle, Cas9 mRNA can be better used as a vaccine but can activate autoimmune retort and various recognition receptors in animal cells [278]. This chemical alteration, like pseudouridine or 5-methylcytidine, will be utilized to control immuno retort without distressing the translation of Cas9 mRNA [322].

Unlike Cas9 plasmid and Cas9 mRNA systems, Cas9-RNP is superseded by skipping transcription and translation, resulting in fast genome engineering followed by lower toxicities [40]. Further, protein-based systems are clinically approved and safe. It is directly introduced in the nucleus via vehicle and can be an idealistic choice for TNBC *in vivo* and *ex vivo* gene editing. Nonetheless, Cas9-RNPs encapsulation and delivery are the biggest challenges for the geneticist and biomedical engineers due to the large size of RNP. Moreover, negatively dense Cas9 RNPs neither pass through the plasma membrane nor have resistance against enzymatic degradation. Conversely, low charge dense RNP is unstable in forming a complex with delivery vehicles. In this main, modification of Cas9/sgRNA in RNP is mandatory that can be achieved *via* recombinant protein technology and conjugation biochemistry. Further, modification in spacer length or its sequence is another thought-provoking strategy to decrease off-targets, immune retorts and heighten RNPs delivery efficiency [39]. Recently, lipid-based, polymeric and inorganic nanovehicles have been widely utilized to deal with these issues. Additionally, some small molecules exhibit crRNA stabilizing properties [27,53,269,272] or enlarging nuclear pores to validate CRISPR/Cas9 cargo delivery [53]. Also, NHEJ-based silencing is more reliable than HDR, which shows only 10% delivery efficacy *in vitro* and non-specific mutation in DNA bases [39]. Thus, these discoveries must be considered while designing the next-generation RNP delivery complex.

Additionally, the successful transfection of Cas protein together with sgRNA at the desired target site is required for fruitful *in vivo* CRISPR-mediated gene editing. For this purpose, the delivery vehicles should have excellent engineering efficacy coupled with low immunogenicity and high capacity of targeted delivery. In this regard, the previously discussed Cas9 plasmid approach is efficient in the mice model, but delivery, editing specificity and control over the expression of Cas9 are limited. Thus, various virus-based delivery methods have been adopted but suffered due to size, safety and packaging limitations. In the last couple of decades, various nanovehicles have been developed in which cationic lipid-based nanovehicles show superior performance and low safety concerns but have synthesis complications and poor colloidal stability in *in vivo* systemic administration [219]. Therefore, the prime focus of the ongoing research programs is to improve and develop effective delivery carriers for CRISPR/Cas gene engineering because low engineering precision and toxicity of existing carriers are the foremost constraints in cancer therapeutic. So far, intracellular transportation of Cas9 remains challenging *in vivo* and as well as in clinical trials.

However, each discussed nanovehicles has its pros and cons due to various biological barriers *in vivo*. The first obstacle is efficient CRISPR/Cas complex encapsulation in the nanocarrier. Cas9 plasmid has a relatively large size about 5–10 ​kDa followed by 1.74 ​× ​10^4^ negative charges, while RNP has a huge size (160 ​kDa) and negatively charged gRNA, enhancing difficulty and multiple delivery issues [232]. For example, CRISPR-nano complex firstly faces the blood-mediated enzymatic degradation of the CRISPR/Cas component, active clearance either via macrophages or mononuclear phagocyte, irregular accumulation of nanovehicles through the opsonisation process, and lastly filtration by glomerulus. Further, CRISPR-nano complex ought to pass through breast tumour-created barriers before reaching targeted sites such as leaky blood vessels, extracellular matrix, high interstitial pressure, hypoxia, densely acidic condition and tumour microenvironment. Furthermore, transcellular phospholipids bilayer, endosome and nuclear pore pose an additional barrier in the delivery of CRISPR/Cas complex [41]. Before transcription or translation of Cas9 sgRNA, nanovehicles have to overawe all mentioned obstacles.

Recently, animals and preclinical studies have revealed promising transfection capability and ease of targeting of nonviral nanovehicles. Further, the organic and inorganic nanovehicles showed excellent colloidal stability coupled with a series of physical and chemical alteration opportunities. For instance, lipids can be engineered as ionic in lipid-based nanocarriers in which CRISPR is efficiently protected and released under acidic environmental conditions [233]. Further, the dynamic surface modification of lipids-based nanocarriers exhibits the superior capability to escape lysosome and RES clearance and targeted transfection [297]. Likewise, cationic lipid NPs efficiently encapsulate and deliver a large cargo of negatively charged CRISPR/Cas sgRNA system than other like viral, plasmid-based and physical transfection methods [232]. On the other hand, cationic lipid nanocarriers induced *in vivo* immune retorts and showed adjuvant immuno retort; thus, lipids-based nanovehicles should be wisely exploited in gene editing. As lipid-based nanocarriers, cationic polymers utilize electrostatic interaction to form cargo complexes with CRISPR/Cas sgRNA. They offer an intelligent programmable delivery vehicle system to overwhelm *in vivo* biological obstructions [307].

Aside from encapsulation, safe and longer circulation in the blood vessels of breast enhance the chances of CRISPR/Cas entry in the tumour cells. A lucrative and even more effective approach is to modify the surface of nanovehicles by diverse kind of FDA approved non-toxic polymer like polyethylene glycol (PEG) [232], chitosan [316], pluronic™ F 127 (PF127) [224,323] and pluronic™ F 68 (PF68). Furthermore, inorganic nanovehicles aid precise targeted delivery of CRISPR/Cas cargo for *in vivo* therapy. The next-generation single-chain antibodies [23,233], antigen-binding fragments [23], aptamers (single-stranded oligonucleotides) [235], AS1411 (G rich DNA oligonucleotide) [324], and peptides [55] can be easefully conjugated on the polymer-coated surface of nanovehicles, which will promote targeting in TNBC. After successful internalization of CRISPR-nano complex via endocytosis, the complex face acidic environment and multiple catalytic enzymes of endosome that can inactive CRISPR/Cas components. In this circumstance, pH-responsive or pH-buffering induce nanovehicles can be better option as some cationic polymer exhibit strong proton sponge impact by their tertiary amine groups and resist against acidic environment of endosome thereby activating Cl¯ and H^+^ antiporter channel. The high amount of Cl¯ and H^+^ ions and water flow create osmotic pressure that eventually disrupted the membrane of endosome and effectively release CRISPR/Cas-nano complex into the cytosol of tumour cell [41,284]. Nonetheless, ridge cargo design formation enhance higher polyelectrolytes that cause dissociation or accumulation of polymer/CRISPR complex in the host body [39].

Another strategy to reduce engulf of the CRISPR-nano complex by endosome is the direct entry into the cytoplasm via receptor-based endocytosis or caveolae-dependent endocytosis [235]. This direct entry of CRISPR/Cas cargo may have more chance to transport into the Golgi complex or endoplasm reticulum that assisted their immediate transcription in the nucleus [41]. Moreover, tumour microenvironment does play a critical role in CRISPR-nano complex delivery. In this connection, inorganic nanovehicles can be exploited because they provide ample control over time-dependent cargo release upon external or internal stimuli such as light [136,283], photothermal [224,323], pH [311], ultrasound [218], thermal, hypoxia [296], ATP and redox-reagent like glutathione-triggered nanovehicles [296]. For instance, gold and silica-based nanocarriers can provide the advantage of PTT and ultrasound-depended transfection via layer-by-layer assembly method. However, both nanocarriers are susceptible to rendering CRISPR/Cas enzyme or release earlier in the bloodstream [40,222,265]. Emerging CRISPR/Cas delivery systems such as magnetic nanocarriers have appealing characteristics that may enhance delivery efficacy of Cas9 and sgRNA. For example, iron oxide have tuneable size, assessable alteration sites, and MR imaging properties [323]. Comprehensively, new emerging delivery approaches are still in infancy, and required deeper insight of their mechanism of action and related limitation, specifically in terms of biosafety and scale-up production need to be explored in detailed for successful clinical translation of CRISPR in TNBC.

## 8 Future perspectives and conclusions

The complete elimination of triple-negative breast carcinoma mass and distinct organ metastasis through clinical trials has not been achieved thus far. Since early diagnosis, treatment, and clinical translation of TNBC are more challenging due to tumour microenvironment, heterogeneity of multiple cellular and oncogenic complexities obfuscate the therapy selection, and monotherapy does not give optimum success. Recently, the CRISPR/Cas cutting-edge gene technology has revolutionized cancer therapeutics; but its delivery is troublesome, off-target impact and genotoxicity due to virus-based transfection threat the biotechnology. The serious concerns regarding CRISPR are error-prone targeting and prolonged aggregation of payload in the genome, and related immunogenicity makes clinical trials in TNBC patients still limited. Undeniably, there are some ethical disquiets regarding genome-engineering technology. First, genome-engineering induces permanent alteration and may cause severe health-related issues in the CRISPR-nano-treated patients if any mistakes occur during the engineering process. Secondly, CRISPR/Cas-nano system is not yet fully established for genetic-engineering, and could not be pragmatized in breast and ovarian carcinoma-bearing patients until more innovative systems become obtainable. Also, it creates enormous inequities and social conflicts if abused or overused, especially in modulating ovarian-based cancer. Further, heritable mutations such as *BRCA*-based genome-edited embryos are not allowed to transfer to the person's uterus. Therefore, before planning CRISPR-nano-mediated engineering in breast/ovarian tumour, one should consider the abovementioned concerns. Additionally, CRISPR-based gene therapy is only utilized for lung cancer and blood cancers such as lymphoma and leukaemia in clinics. Thus, several years have been needed by common society to adapt and accept gene-engineering-based alteration.

Apart from ethical concerns, transcription-activator like effector (TALENs) and zing-finger nucleases (ZFNs)-based gene editing are more expensive, generate permanent numerical and structural mutations, time-consuming and even not practical for mass production. whereas, CRISPR/Cas technology is rapid, low-cost, relatively more accurate and efficient than the other gene manipulation technologies. Thus, CRISPR/Cas gene engineering must be established through a wide range of clinical trials, which will restore the condition of TNBC than presently accessible treatment tactics. Moreover, several biological molecules, targeting agents, organic and inorganic nanocarriers are developed, which enhance the transfection efficacy, aid targeting specificity, and silencing the gene or mRNA expression either directly interrelating with driver genes or through changing several signalling molecules and pathways accountable for TNBC progression.

Nonetheless, innumerable challenges discoursed so far — the therapeutics tactic for TNBC ought to emphasize the effective abolition of carcinoma, related tumour subpopulation, and allied blood vessels via synergistic advanced gene technologies. The treatment objectives have implemented the perception of personalized gene therapy for each patient or subpopulation of tumour cells. The elementary consideration of genomics exploring the stimulus of the cellular signalling cascade in tumour propagation and resistance will expose a novel arena for TNBC therapy. Moreover, tumour microenvironment is the most important arena of oncology research. In this main, CRISPR/Cas pro-codon library aid the analysis of tumour microenvironment by silencing a series of genes in a mouse model and revealing how each gene influences tumor growth, immune composition and histopathology [325]. Its robust knockout and knock-in technology resolved the central dogma of immunosuppression and showed a relation between loss of function genes and tumour microenvironment. Also, an individual's immune responses play a fundamental part in defending from carcinogenesis. Recently, the successful application of CAR T-cell and noncoding RNAs-based immuno-genetic engineering requires an exceptional methodological advancement in molecular genetics, pharmacogenomics, nanotechnology, and chemistry.

In addition, an optimal CRISPR/Cas9-sgRNA-centered genome editing should be comprehensively analysed for MHC-I, II, T-cells, and macrophages population's responses, chemically altered to increase pharmacodynamics, and pharmacokinetics, transfected under contemplation of active biodistribution, and clathrin or endolysosomal escape mechanistic, low off-target and high determination of target specificity, and be treated at an optimum level to get the desired impact. Noteworthy improvements in these fields have been made exclusively for CRISPR/Cas therapeutics; nonetheless, its efficacious transformation depends on additional interdisciplinary advancements to improve on-target impact and payload target delivery. More obviously, the stratagems mentioned above can be incorporated by nanomaterials-based photothermal therapy and imaging to securely locate and classify the biological distribution of the CRISPR@nano complex.

Conversely, high therapeutic cargo envisions within the nuclear spaces enhance the ultimate potential of pioneering technology that would fundamentally be based on the innovation and design of dynamic multi-structured nanomaterials systems. For instance, exotic intrinsic properties holding gold biosensors can be unlocked the ssDNase by CRISPR/Cas12a and examine telomerase activity in breast carcinoma subtypes [27,326]. Also, nucleic acid-based multiplex system [271], cell-penetrating peptides, and carbon nanotubes will potentially deliver CRISPR/Cas-sgRNA cargo efficiently. Importantly, plant-derived exosomes [327], and extracellular vesicles-based nanovehicle — a potent platform for intercellular crosstalk and communication, engineered vesicles can be loaded and safely delivered CRISPR/Cas9 complex at the targeting sites [[328], [329], [330]]]. Similarly, a tuneable magnetic system can be devised by a magnetic field that induces controllable delivery of CRISPR/Cas9 along with advanced imaging properties, mass production and precise transfection in TNBC cells will be more effective. These robust vehicles advance TNBC treatment outcomes that will help enhance the patient's lifespan. However, only a limited number of inorganic nanovehicle are FDA approved for clinical translation due to their apparent toxicities. Moreover, safe, simple, cost-effective, and eco-friendly synthesis of inorganic nanoparticles are unmet needs of material science.

Moreover, organic nanovehicles are immuno-friendly, biocompatible and biodegradable but suffer due to poor therapeutic/drug loading capacity and structural drawbacks. Mostly, their ultra-structures have been destroyed during packaging/loading process of the payload. Thus, advancement in the development of nanovehicles with superior biocompatibility and low immunogenicity, such as biomimetics [285] may be incorporated to have a better option for the delivery of CRISPR/Cas complex at the desired targeting site. Further, the transcriptomes, proteomes, driver genes, and extracellular matrix biomolecules are reported as resilient either in masking other addiction genes expression or inducing TNBC prognosis; however, the previous data lack the connecting links among molecules that have been explored as oncogenes. Moreover, a better understanding of the singling cascades, material interaction with organic molecules, and improvements in material technology will eventually develop a more idyllically targeting system in the future.

Furthermore, good manufacturing practice (GMP), ethical compliance, and low cost are among the most critical concerns in designing the CRISPR/Cas complex for its clinical translation. To tackle this, new strategies are required to develop in which clinically appropriate GMP-compliant material such as clinical grade Cas9 and sgRNA will be utilized [331]. Now, National Institute of Health (NIH) has invested more than billions of dollars to achieve the abovementioned issues. For this, several companies developed cGMP unit to produce desirable nuclease for research and diagnoses. However, these CRISPR/Cas nucleases exhibit leaching of divalent cations during purification, which can cause serious issues in drug production practices. Recently, several off-the-shelf techniques have been developed to scale up the previous CRISPR/Cas gene manipulation method. Whereas, some off-targets related unforeseen toxicities are also noticed, especially in the case of CRISPR-based T cell immunotherapies. Further, the off-target based toxicities will be dealt with by utilizing other host mechanisms than SpCas9 and *Streptococcus aureus* (SaCas9). Some improvements have been made, such as SpyFi™ Cas9 (Integrated DNA Technologies Inc.) that can replace single amino acid, resulting in prominent decreases in off-target effect compared to wild-type SpCas9, which is the main concern of gene therapy.

In summary, the CRISPR-nano complex brings foremost progress in the analysis and therapy of TNBC. The use of CRISPR/Cas9 in the cell culture study, gene manipulation, establishment of animal models, and patient-derived xenograft (PDX) models for TNBC therapy, rapidly augmented due to the system easiness of designing, targeting, recognizing and engineering multiplexed gene target, and cost-effectiveness. However, microbe-based and physical methods are common CRISPR/Cas intracellular delivery approaches. Nonetheless, recent inundation research disadvantages have been reported for transfection approaches, resulting in the development of effective and safe payload delivery experiencing serious challenges that are prerequisite to be tackled. In recent times, nanovehicle for CRISPR/Cas9 targeted delivery has been fascinated by remarkable courtesy. The copious advanced nanomaterial transfection systems for example; protein, lipids, nanolipogel, polymer, and inorganic materials, have been aimed. Among them, iron, gold, and cationic nanolipogel-based delivery systems have the most prospective in imminent practical solicitations. The CRISPR/Cas9 gene-editing technology has spotted a rebellion in chemo-immunotherapy. Furthermore, this powerful gene engineering technology can be overwhelmed numerous delivery complications and aid success in the forthcoming clinical translation. Consequently, it is believed that the synergy of CRISPR/Cas9 and nanomaterial-dependent transfection embraces the substantial potential for the prospect of TNBC therapy.

## Author contributions

JF and MZI conceptualized; JF initial drafted, structured, and written the manuscript; MZI, NSH, RZ, QZ and XDK critically analysed; MZI and NSH edited; MZI and XDK supervised the study; JF, MZI and XDK project funding acquisition.

## Declaration of competing interest

The authors declare that they have no known competing financial interests or personal relationships that could have appeared to influence the work reported in this paper.

## Data Availability

Data will be made available on request.
